# RC-Mamba: regime-conditioned selective state-space models for probabilistic time-series forecasting

**DOI:** 10.3389/frai.2026.1903497

**Published:** 2026-09-23

**Authors:** Waleed Soliman, Zhiyuan Chen, Colin Johnson, Sabrina Wong

**Affiliations:** 1 School of Computer Science, University of Nottingham Malaysia, Semenyih, Selangor, Malaysia; 2 School of Computer Science, University of Nottingham, Nottingham, United Kingdom; 3 Hy-Alpha Sdn Bhd, Selangor, Malaysia

**Keywords:** financial time series, hidden Markov model, Mamba, probabilistic forecasting, regime conditioning, state-space models

## Abstract

Selective state-space models (SSMs) such as Mamba achieve linear-time sequence modeling through input-dependent recurrence, yet they apply the same dynamics regardless of the latent regime generating the data—a structural mismatch for non-stationary signals whose statistics shift abruptly between regimes. We introduce *RC-Mamba*, a probabilistic forecaster whose selective-SSM backbone is dynamically conditioned on the posterior over a latent regime variable. Regime posteriors from a Student-*t* emission Hidden Markov Model with a sticky Dirichlet prior modulate the SSM discretisation step Δ_*t*_ together with the input and readout matrices **B**_*t*_ and **C**_*t*_, so the recurrence adapts its memory decay, input sensitivity, and output emphasis to the prevailing market state. An entropy-based scale-modulation component, the Regime Uncertainty Gate, is evaluated as a route from posterior sharpness to interval width, but matched controls do not identify a performance gain for it over posterior-independent scaling. A hierarchical Feature-wise Linear Modulation (FiLM) embedding captures asset-specific and sector-level structure without an explicit asset-class prediction objective. Evaluated on 18 US-listed ETFs spanning five asset classes under five calendar-aligned expanding-window walk-forward folds (test years 2021–2025), RC-Mamba achieves a mean Continuous Ranked Probability Score (CRPS) of 0.00532, improving on the Vanilla Temporal Fusion Transformer by 4.7 %, DeepAR by 8.9 %, S-Mamba by 15.2 %, and an HMM-conditioned DeepAR by 18.5 %. All models share robust-MAD preprocessing, a common ceiling of 11,450 optimizer updates with validation-based early stopping, and a 30-business-day origin embargo equal to the maximum forecast horizon, so neither preprocessing nor training budget confounds the architectural comparison. Significance is assessed on 1,110 synchronized forecast origins using a moving-block bootstrap that respects serial and cross-sectional dependence rather than treating overlapping horizons as independent; every comparison remains significant after Holm correction (*p*_Holm_ = 0.006). RC-Mamba also attains the best Winkler_80_ score (0.0364) with PICP_80_ = 0.781 and PINAW_80_ = 0.024, and recovers three interpretable regimes (Calm, Transition, Stress) that assign every one of the 25 observed April 2025 trading days to the Stress state. Matched single-fold ablations show that removing regime conditioning degrades CRPS by 2.05 % in the most volatile test year, although several component contrasts change sign across folds and are therefore reported as design trade-offs rather than identified causal effects. A one-day-lagged long-only backtest over 1,255 out-of-sample trading days returns 8.89 % annualized against 6.56 % for an equal-weight benchmark, but its annualized alpha of 1.18 % is not statistically significant (*p* = 0.506) and remains insignificant under 1–10 basis-point costs; the economic evidence is therefore suggestive rather than conclusive. Transfer to non-financial domains is left to future work.

## 1 Introduction

Sequence models that adapt their computation to the input have advanced rapidly, with selective state-space models (SSMs) such as Mamba (Gu and Dao, 2023) offering linear-time recurrence whose dynamics are modulated by the current input. Yet these architectures remain *regime-agnostic*: the same recurrence is applied whether the underlying data-generating process is in a calm, transitional, or stressed state. For non-stationary signals—where regime shifts change the joint distribution of the observed variables rather than merely adding noise—this is a structural limitation. We study the problem in financial markets, a canonical non-stationary, regime-switching domain: economy-wide shifts (monetary tightening, credit stress, geopolitical disruption) alter the joint distribution of prices, volatilities, and cross-asset correlations. We model ETF returns as observations generated under latent risk regimes inferred from macro-financial stress indicators, and condition a selective state-space model on these regime posteriors for probabilistic forecasting.

Current deep learning architectures for time-series forecasting—TFTs (Lim et al., 2021), DeepAR (Salinas et al., 2020), N-BEATS (Oreshkin et al., 2020)—receive no signal about whether the macro environment is calm, transitional, or stressed. Regime adaptation must emerge implicitly through learned weights, placing a heavy burden on model capacity and training data. Mamba (Gu and Dao, 2023) offers an efficient alternative to attention-based processing with input-dependent discretisation and linear-time complexity, yet it too lacks structural awareness of the prevailing market regime.

These two research traditions—regime-switching econometrics (Hamilton, 1989; Bulla et al., 2011) and deep temporal forecasting—have developed largely in isolation. Classical regime models produce interpretable state assignments but cannot scale to high-dimensional multi-asset forecasting; deep architectures handle rich feature sets but lack explicit regime awareness. RC-Mamba bridges this gap. The core novelty is mechanistic rather than a recombination of existing blocks: instead of using regime estimates as an auxiliary input or a *post-hoc* data partition, we concatenate the regime posterior to the SSM input *before* the parameter projections, so the recurrence's discretisation step Δ_*t*_, input matrix **B**_*t*_, and readout matrix **C**_*t*_ themselves become regime-adaptive at every time step—a structurally minimal change that leaves the linear-time scan unaltered. To our knowledge, no prior selective state space model conditions its recurrence parameters on latent-regime posteriors in this way. Matched single-fold ablations support the narrow form of this claim: conditioning the projections outperforms appending the same posterior as an ordinary input, which is the one component contrast bearing directly on the architectural claim. We claim novelty specifically for conditioning a selective SSM's (Δ, **B**, **C**) projections on HMM posterior probabilities, not for regime-aware neural forecasting in general, which has an established literature in switching dynamical systems, mixture-of-experts routing and FiLM-style conditioning. The mechanism is in principle applicable to any non-stationary sequence whose regimes can be estimated from observable covariates, though we evaluate it only in the financial setting studied here.

**Research questions**. We address three research questions:

**RQ1** Can conditioning the Mamba SSM parameters (Δ, **B**, **C**) on HMM regime posteriors improve probabilistic forecast accuracy over regime-agnostic alternatives?
**RQ2** Does a Student-*t* emission HMM with a sticky Dirichlet prior produce economically interpretable and temporally persistent regime assignments?
**RQ3** Does posterior-entropy-based scale modulation improve calibration beyond posterior-independent scale controls?

**Contributions**. We make six contributions:

1. **RC-Mamba Block**. A regime-conditioned Mamba SSM in which Δ, **B**, and **C** are projected from the concatenation of temporal features with HMM regime posteriors γ_*t*_ (Section 3.3), injecting macro-structural information directly into the SSM recurrence.
2. **Student-t** **HMM with Sticky Dirichlet Prior**. A log-space forward-filtering HMM with Student-*t* emissions for heavy-tailed financial observables and a sticky Dirichlet prior that encourages regime persistence (Section 3.2).
3. **Regime Uncertainty Gate (RUG), evaluated but not supported**. A three-feature gate—posterior entropy, peak probability, and transition uncertainty—that modulates predictive scale within [0.5, 2.0], intended to link epistemic regime uncertainty to aleatoric forecast uncertainty (Section 3.6). We report it as an evaluated auxiliary component rather than a validated one: in both matched ablation batches a posterior-independent learned global scale performs at least as well (Section 4.7).
4. **Hierarchical FiLM Embedding**. A feature-wise linear modulation layer combining sector- and ticker-level embeddings, enabling the model to learn shared factor exposures within asset classes while preserving asset-specific residuals—without an explicit asset-class prediction objective (Section 3.5).
5. **Regime-Conditioned Interpretability**. A dual-lens framework combining VSN selection weights with grouped permutation SHAP attribution, decomposed by regime state, which shows that regime conditioning reorders factor importance across market states rather than rescaling a single profile (Sections 3.8 and 4.12).
6. **Walk-Forward Evaluation**. An experimental protocol covering 18 ETFs across five asset classes under five calendar-aligned expanding-window walk-forward folds with a 30-business-day embargo equal to the maximum forecast horizon, origin-level moving-block bootstrap inference with Holm correction [a HAC-adjusted Diebold–Mariano test (Diebold and Mariano, 1995) as a secondary check], and calibration analysis via proper scoring rules (Gneiting and Raftery, 2007) (Section 3.10).

**Results preview**. Across 18 ETFs spanning equities, fixed income, commodities, and international markets, RC-Mamba achieves a mean CRPS of 0.00532 under five calendar-aligned walk-forward folds, with an origin-level moving-block bootstrap confirming statistically significant gains over all six comparators (*p*_Holm_ = 0.006; Section 4). Matched single-fold ablations show that removing regime conditioning entirely is the most damaging single change (+2.05% CRPS in the most volatile test year) and that conditioning the projections beats appending the same posterior as an ordinary input (+0.81%). The SiLU gate is retained as the pre-selected configuration rather than as a demonstrated improvement: the matched contrast marginally favors the gate-enabled variant. The learned HMM recovers three economically interpretable regimes, and *post-hoc* SHAP attribution shows regime-dependent factor structure—broad economic-uncertainty and monetary-policy features lead under Stress, Fed balance-sheet and labor-market features under Transition—rather than a single pattern rescaled across states.

## 2 Related work

RC-Mamba draws on five threads: deep learning for time series, structured state-space models, regime-switching econometrics, probabilistic forecast evaluation, and interpretable machine learning.

### 2.1 Deep learning for time series forecasting

LSTMs (Hochreiter and Schmidhuber, 1997) long dominated financial sequence modeling, and the Transformer (Vaswani et al., 2017) replaced recurrence with self-attention at quadratic cost, prompting efficient variants such as the Informer (Zhou et al., 2021) (ProbSparse attention) and Autoformer (Wu et al., 2021) (series decomposition with auto-correlation); PatchTST (Nie et al., 2023) segments series into patches in a channel-independent encoder. More recent models include TimesNet (Wu et al., 2023) (2D multi-period folding), iTransformer (Liu et al., 2024) (attention across variables), and TimeMixer (Wang S. et al., 2024) (multiscale decomposable mixing), while on the probabilistic side TimeGrad (Rasul et al., 2021) couples an autoregressive RNN with denoising diffusion and Lag-Llama (Rasul et al., 2023) pursues pre-trained foundation models. Two architectures are central for covariate handling: the Temporal Fusion Transformer (TFT) (Lim et al., 2021), with variable-selection and gated residual networks for interpretable multi-horizon prediction, and DeepAR (Salinas et al., 2020), an autoregressive LSTM emitting per-step distribution parameters; N-BEATS (Oreshkin et al., 2020) matches these on univariate benchmarks but admits no exogenous covariates. None of these models exposes a structured parameter-projection interface comparable to the (Δ_*t*_, **B**_*t*_, **C**_*t*_) projections RC-Mamba builds on, and all implicitly assume stationarity: regime adaptation must emerge from the training signal alone, which burdens capacity and requires each regime—including rare stress events—to be well represented in the training window.

### 2.2 Structured state space models

Structured state-space models (SSMs) offer a path between recurrence and attention. S4 (Gu et al., 2022) showed that a HiPPO-initialized linear state-space recurrence, trained through a convolutional dual view, captures long-range dependencies; earlier, deep state-space models (Rangapuram et al., 2018) combined classical linear dynamics with neural emissions for calibrated probabilistic forecasts, but without input-dependent selection. Mamba (Gu and Dao, 2023) makes the recurrence parameters *input-dependent*—the discretisation step Δ, input matrix **B**, and readout matrix **C** are projected from the current token—achieving linear-time complexity via a hardware-aware scan, and Mamba-2 (Dao and Gu, 2024) recasts the scan through a duality with linear attention. Follow-up time-series backbones — TimeMachine (Ahamed and Cheng, 2024), S-Mamba (Wang Z. et al., 2025), and SiMBA (Patro and Agneeswaran, 2024)—adopt Mamba's selection mechanism unchanged, projecting from the hidden state alone with no provision for external structural conditioning, and finance applications of SSMs remain nascent and do not explore regime conditioning. Because Δ, **B**, and **C** are already linear projections of the hidden state, *concatenating* an external regime signal before projection is a structurally minimal change that makes the SSM's memory decay, input sensitivity, and readout emphasis regime-adaptive without altering the scan algorithm—the architectural foundation of RC-Mamba.

### 2.3 Regime-switching models in econometrics, finance, and ETF prediction

Latent-state models have shaped financial econometrics since Hamilton's Markov-switching autoregression (Hamilton, 1989), which Kim (1994) generalized to a switching state-space form with standard filtering and smoothing. In volatility modeling, ARCH (Engle, 1982) and GARCH (Bollerslev, 1986) assume a single regime, whereas Hamilton and Susmel (1994) and the Markov-switching GARCH of Gray (1996) let variance dynamics differ across states. Such models underpin regime-aware asset allocation: Ang and Bekaert (2002) document cross-market correlations rising in bear states, and Guidolin and Timmermann (2007) show regimes dominate multivariate allocation at longer horizons. On specification, a simple two-state Gaussian HMM reproduces stylised facts of daily returns (Rydén et al., 1998), and Student-*t* emissions better capture excess kurtosis (Bulla et al., 2011). For ETFs, our prior work shows that macro indicators improve DeepAR forecasts (Soliman et al., 2023) and that FinBERT sentiment with fixed-seed sampling adds further gains (Soliman et al., 2025), but neither used regime switching; machine-learning asset pricing (Gu et al., 2020) likewise omits explicit regime conditioning. Existing regime-aware neural methods typically use the HMM as a *post-hoc* filter or to partition training data rather than injecting posteriors into temporal processing. Classical regime models are further limited by dimensionality—intractable for a diversified 18-ETF, 30-feature, fully distributional setting—whereas RC-Mamba treats the HMM as a lightweight regime-detection front end conditioning a deep backbone, retaining economic interpretability while scaling.

### 2.4 Probabilistic forecasting and proper scoring rules

Financial decision-making requires calibrated predictive distributions, evaluated with proper scoring rules. Matheson and Winkler (1976) introduced the continuous ranked probability score (CRPS), and Gneiting and Raftery (2007) formalized the principle that forecasts should be as sharp as possible subject to calibration; the Winkler score (Winkler, 1972) assesses prediction-interval quality. In deep learning, DeepAR (Salinas et al., 2020) emits parametric distributions and the TFT (Lim et al., 2021) quantiles, while conformal prediction (Romano et al., 2019) offers distribution-free coverage, though its extension to multi-step, regime-shifting series remains open. RC-Mamba emits Student-*t* parameters and is evaluated on CRPS, Winkler, PICP, and PINAW.

### 2.5 Interpretable machine learning in finance

Interpretability in financial forecasting serves both regulatory and scientific ends. SHAP (Lundberg and Lee, 2017), grounded in cooperative game theory, assigns additive feature attributions, and LIME (Ribeiro et al., 2016) fits local surrogate models; both are usually applied unconditionally, yielding a single importance ranking. Yet in regime-dependent markets feature importance is itself state-dependent—monetary-policy variables may matter little in calm periods but dominate under stress. The TFT's variable-selection weights (Lim et al., 2021) measure input *selection* rather than output *contribution*. RC-Mamba combines intrinsic VSN selection weights with *post-hoc* SHAP attributions, stratified by HMM regime state, yielding regime-conditioned importance profiles—a dual-lens, regime-aware framework not, to our knowledge, previously proposed.

### 2.6 Summary

Supplementary Table S12 situates RC-Mamba relative to the most closely related methods along six dimensions.

The table makes the gap concrete: no prior approach combines a structured state space backbone with explicit regime conditioning, exogenous covariate support, distributional outputs, multi-asset coverage, and walk-forward evaluation. RC-Mamba occupies this intersection.

## 3 Materials and methods

This section presents the complete architectural and mathematical formulation of the RC-Mamba model. Figure 1 provides a bird's-eye view of the six-tier dataflow, from raw market inputs through regime-conditioned state-space processing to probabilistic Student-*t* outputs.

**Figure 1:**
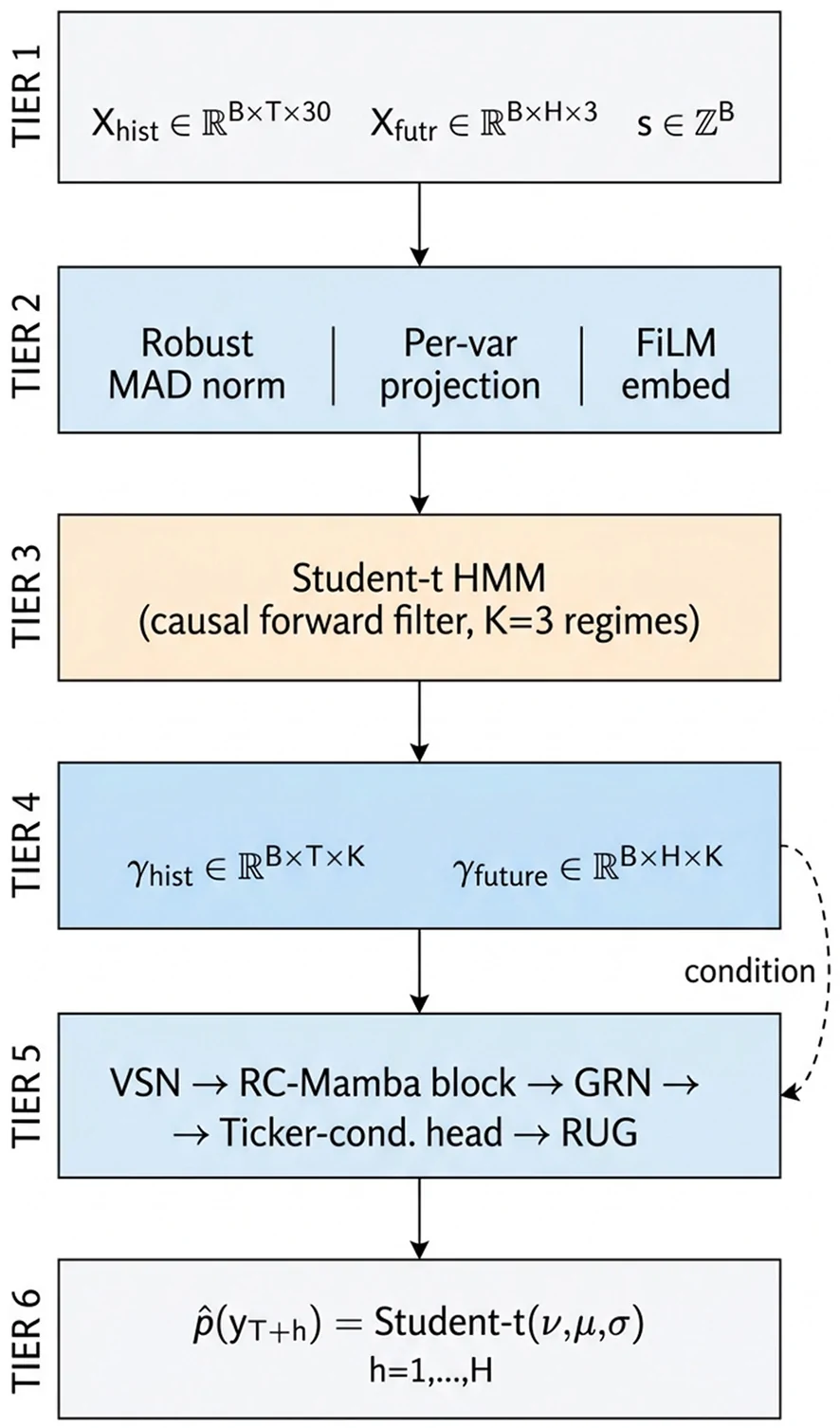
RC-Mamba system overview. Six processing tiers transform raw market inputs into probabilistic Student-*t* forecasts. Regime posteriors from the HMM (Tier 3) condition the backbone via concatenation at the SSM parameter projections.

We begin with the problem statement (Section 3.1), then detail the regime detection module (Section 3.2), the regime-conditioned Mamba block (Section 3.3), the variable selection and context construction mechanisms (Section 3.4), the hierarchical FiLM embedding (Section 3.5), the Regime Uncertainty Gate (Section 3.6), the Student-*t* distribution head (Section 3.7), and finally the four-stage training protocol (Section 3.9).

### 3.1 Problem formulation

We address the multi-asset, multi-step probabilistic forecasting problem. Let {*y*_*i,t*_} denote the daily log-return of asset *i* ∈ {1, …, *I*} at time *t*. The model receives three categories of inputs:

- *Historical covariates* ${\text{X}}_{\text{hist}}\in {\mathbb{R}}^{B\times T\times V_{h}}$, comprising *V*_*h*_ = 30 time-varying features: price-derived indicators (OHLCV, moving averages, volatility, buy/sell signals) and macroeconomic series (interest rates, monetary aggregates, inflation proxies, credit spreads, currency indices, equity market volatility sub-indices).
- *Future covariates* ${\text{X}}_{\text{futr}}\in {\mathbb{R}}^{B\times H\times V_{f}}$, with *V*_*f*_ = 3 calendar features known at forecast time (day-of-week, week-of-year, month), passed through without normalization.
- *Static identifiers* **s** ∈ ℤ^*B*^, encoding per-series ticker and GICS sector identifiers.

The model outputs, for each asset and each forecast horizon step *h* = 1, …, *H*, the parameters of a Student-*t* predictive distribution (Equation 1):


$$\begin{array}{l} p\left(y_{T+h}\right)=\text{Student-}t\left(\nu_{T+h},\mu_{T+h},\sigma_{T+h}\right), \end{array}$$ (1)

where ν > 3 denotes the degrees of freedom, μ the location, and σ > 0 the scale. The constraint ν > 3 guarantees finite variance (the standard Student-*t* moment conditions are ν > 2 for finite variance and ν > 4 for finite kurtosis), which is more than sufficient for the first-moment regularity required by the CRPS formulation used in Stage 4 training.

Supplementary Table S13 summarizes the key notation used throughout this section.

### 3.2 Regime detection module: Student-*t* log-space HMM

The regime detection module is a *K*-state Hidden Markov Model whose latent states encode distinct market regimes (e.g., calm, transition, stress). It operates on a triplet of macro-financial observables ${\text{o}}_{t}=\left[\text{VIXCLS},\text{BAMLH0A0HYM2},\text{DGS10}-\text{DGS2}\right]\in {\mathbb{R}}^{D}$ with *D* = 3, and produces causal regime posteriors $\gamma_{t}\in \Delta^{K-1}$ that condition the downstream forecasting backbone.

Supplementary Figure S2 illustrates the forward filtering process, showing the two-column temporal unrolling of predict–update–normalize operations across time steps.

#### 3.2.1 Student-*t* emission model

Each regime *k* ∈ {1, …, *K*} emits observations via a product of independent univariate Student-*t* distributions, one per observation dimension *d* ∈ {1, …, *D*} (Equation 2):


$$\begin{array}{l} \log p(o_{t}|z_{t}=k)\\ =\sum_{d=1}^{D}[\log\Gamma (\frac{\nu_{k}+1}{2})-\log\Gamma (\frac{\nu_{k}}{2})-\frac{1}{2}\log\text{​}(\nu_{k}\;\pi \;\sigma_{k,d}^{2})\\ \;-\frac{\nu_{k}+1}{2}\log\text{​}(1+\frac{{\left(o_{t,d}-\mu_{k,d}\right)}^{2}}{\nu_{k}\sigma_{k,d}^{2}})], \end{array}$$ (2)

where the per-regime emission parameters are constrained as Equations 3, 4:


$$\begin{array}{l} \sigma_{k,d}^{2}=\mathrm{softplus}\left(\theta_{k,d}^{\text{var}}\right)+\varepsilon_{\text{floor}},\;\varepsilon_{\text{floor}}=10^{-4}, \end{array}$$ (3)

$$\begin{array}{l} \nu_{k}=\mathrm{softplus}\left(\theta_{k}^{\text{df}}\right)+2. \end{array}$$ (4)

The degrees-of-freedom parameters $\theta_{k}^{\text{df}}$ are initialized at 1.0, yielding ν_*k*_ ≈ 3.31 at the start of training. The constraint ν_*k*_ > 2 guarantees finite emission variance. Means μ_*k,d*_ are initialized via linspace(−2, +2, *K*) per dimension, providing well-separated initial regimes.

The choice of Student-*t* over Gaussian emissions is motivated by the well-documented heavy tails of financial volatility and credit spread distributions (Bulla et al., 2011), which the Student-*t* likelihood captures through its additional degrees-of-freedom parameter.

#### 3.2.2 Log-space transition matrix

The row-stochastic transition matrix **A** ∈ ℝ^*K*×*K*^ is parameterised via an unconstrained matrix **A**^raw^ ∈ ℝ^*K*×*K*^ (Equation 5):


$$\begin{array}{l} \log A_{j,k}=\log\mathrm{softmax}{\left({\text{A}}_{j,:}^{\text{raw}}\right)}_{k}. \end{array}$$ (5)

When the sticky Dirichlet prior is active, **A**^raw^ is initialized to 2**I**_*K*_ (diagonal bias favoring self-transitions); otherwise all entries start at zero (uniform transitions).

#### 3.2.3 Causal forward filtering

For each time step *t* = 1, …, *T*, given the previous log-posterior $\log\gamma_{t-1}\in {\mathbb{R}}^{B\times K}$, the filter executes three operations (Equations 6–8):


$$\begin{array}{l} \text{Predict:}\log{\tilde{\gamma }}_{t,k}={\mathrm{logsumexp}}_{j}\left[\log\gamma_{t-1,j}+\log A_{j,k}\right], \end{array}$$ (6)

$$\begin{array}{l} \text{Update:}\log{\hat{\gamma }}_{t,k}=\log{\tilde{\gamma }}_{t,k}+\log p\left({\text{o}}_{t}|z_{t}=k\right), \end{array}$$ (7)

$$\begin{array}{l} \text{Normalize:}\log\gamma_{t,k}=\log{\hat{\gamma }}_{t,k}-{\mathrm{logsumexp}}_{k^{\prime }}\left[\log{\hat{\gamma }}_{t,k^{\prime }}\right]. \end{array}$$ (8)

The loop is strictly causal: γ_*t*_ depends only on **o**_1:*t*_ with no backward smoothing pass, ensuring zero look-ahead bias. The full historical posterior sequence is Equation 9:


$$\begin{array}{l} \gamma_{\text{hist}}=\left[\gamma_{1},\ldots ,\gamma_{T}\right]\in {\mathbb{R}}^{B\times T\times K}. \end{array}$$ (9)

#### 3.2.4 Future regime propagation

The terminal posterior γ_*T*_ is projected *H* steps forward via iterated transition-matrix multiplication with re-normalization (Equation 10):


$$\begin{array}{l} \gamma_{T+h}=\frac{\gamma_{T+h-1}\text{A}}{\left||\gamma_{T+h-1}\text{A}\right|{|}_{1}},\;h=1,\ldots ,H, \end{array}$$ (10)

yielding $\gamma_{\text{future}}\in {\mathbb{R}}^{B\times H\times K}$. Gradient flow through γ_future_ is enabled, so the forecast loss trains the transition dynamics end-to-end.

#### 3.2.5 Sticky Dirichlet prior

A Dirichlet prior on each transition row encourages regime persistence (Equation 11):


$$\begin{array}{l} L_{\text{Dir}}=\overset{K}{\underset{j=1}{\sum }}\left[\left|\log B\left(\alpha_{j}\right)\right|-\overset{K}{\underset{k=1}{\sum }}\left(\alpha_{j,k}-1\right)\log A_{j,k}\right], \end{array}$$ (11)

where α_*j,j*_ = α_diag_ (e.g., 2.0) and α_*j,k* ≠ *j*_ = α_off_ (e.g., 0.1), and $\log B\left(\alpha_{j}\right)=\underset{k}{\sum }\log\Gamma \left(\alpha_{j,k}\right)-\log\Gamma \left(\underset{k}{\sum }\alpha_{j,k}\right)$. Minimizing $L_{\text{Dir}}$ pulls transition mass toward the diagonal, promoting persistent regimes characteristic of financial markets.

#### 3.2.6 Transition entropy regularization

To prevent regime collapse to a single absorbing state, the row entropy of the transition matrix is maximized (Equation 12):


$$\begin{array}{l} L_{\text{entropy}}=-\frac{1}{K}\overset{K}{\underset{j=1}{\sum }}H\left({\text{A}}_{j,:}\right),\;H\left({\text{A}}_{j,:}\right)=-\overset{K}{\underset{k=1}{\sum }}A_{j,k}\log A_{j,k}. \end{array}$$ (12)

Minimizing $L_{\text{entropy}}$ (the negative mean row entropy) counterbalances the sticky prior, preventing transition rows from collapsing to one-hot vectors.

#### 3.2.7 Self-transition penalty

A complementary ReLU-style penalty caps diagonal entries (Equation 13):


$$\begin{array}{l} L_{\text{self}}=\frac{1}{K}\overset{K}{\underset{k=1}{\sum }}{\left[\max\left(0,A_{k,k}-\tau \right)\right]}^{2},\;\tau =0.80, \end{array}$$ (13)

preventing any single regime from becoming excessively persistent even when the Dirichlet prior is active. The three HMM regularization losses ($L_{\text{Dir}}$, $L_{\text{entropy}}$, $L_{\text{self}}$) jointly encourage a well-conditioned transition matrix with distinct, persistent, yet non-degenerate regimes.

### 3.3 Regime-conditioned Mamba block (RC-Mamba block)

The RC-Mamba block is the core temporal engine of the architecture. It replaces the multi-head attention mechanism of the Temporal Fusion Transformer (Lim et al., 2021) with a structured state space model [Mamba (Gu and Dao, 2023)] whose discretisation and recurrence parameters are dynamically conditioned on the HMM regime posteriors.

Figure 2 details the internal dataflow, from the pre-normalized input through regime-conditioned SSM parameter projections to the residual output.

**Figure 2:**
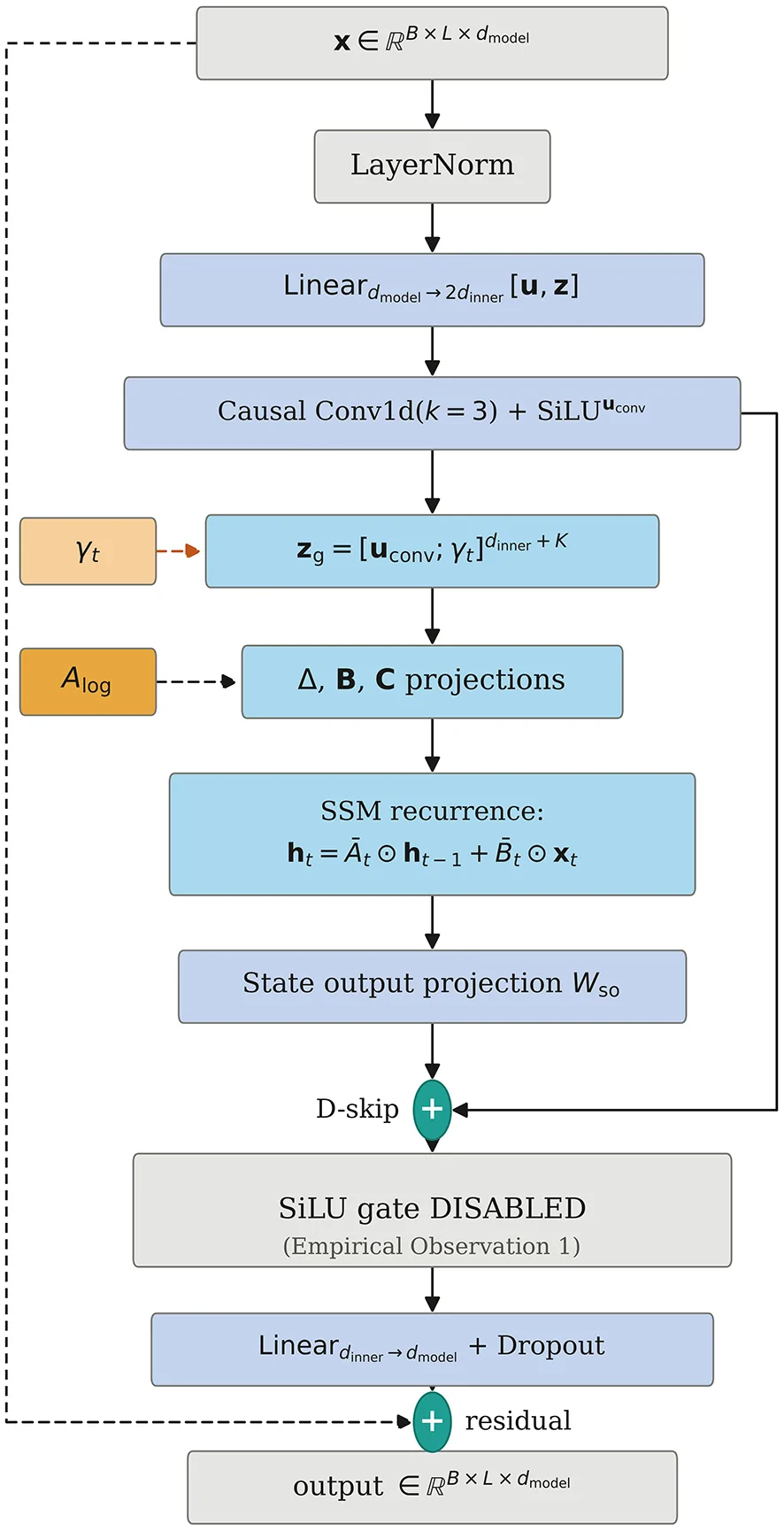
RC-Mamba block internal dataflow. Regime posteriors γ_*t*_ are concatenated with the convolved features before projecting to all three SSM parameters (Δ, **B**, **C**). The SiLU gate is disabled in the selected configuration (Empirical Observation 1); the D-skip provides a direct feedthrough path. A pre-normalization residual connection completes the block.

#### 3.3.1 Continuous-time state space model

The block is based on a *diagonal* state-space model. In continuous time (Equation 14):


$$\begin{array}{l} \overset{\cdot }{\text{h}}\left(\tau \right)=\text{A}\cdot \text{h}\left(\tau \right)+\text{B}\left(\tau \right)\cdot x\left(\tau \right),\;y\left(\tau \right)=\text{C}\left(\tau \right)\cdot \text{h}\left(\tau \right), \end{array}$$ (14)

where $\text{A}\in {\mathbb{R}}^{d_{\text{state}}}$ is a learned diagonal transition, and **B**(τ), **C**(τ) are input-dependent (selective), following the Mamba formulation (Gu and Dao, 2023). The diagonal structure reduces the state transition from $O\left(d_{\text{state}}^{2}\right)$ to *O*(*d*_state_) per step.

#### 3.3.2 Pre-normalization and input projection

The input sequence $\text{x}\in {\mathbb{R}}^{B\times L\times d_{\text{model}}}$ (where *L* = *T* + *H*) is first layer-normalized, then linearly projected and split into two paths (Equations 15, 16):


$$\begin{array}{l} \hat{\text{x}}=\mathrm{LayerNorm}\left(\text{x}\right), \end{array}$$ (15)

$$\begin{array}{l} \left[\text{u},\text{z}\right]={\mathrm{Linear}}_{d_{\text{model}}\to 2d_{\text{inner}}}\left(\hat{\text{x}}\right), \end{array}$$ (16)

where $\text{u}\in {\mathbb{R}}^{B\times L\times d_{\text{inner}}}$ serves as the main processing path and **z** is the gate path. In the selected configuration, the SiLU gate pathway **z** is **disabled** (see Section 3.3.9).

#### 3.3.3 Causal convolution

A depth-wise causal convolution with kernel size 3 provides local temporal mixing (Equation 17):


$$\begin{array}{l} {\text{u}}_{\text{conv}}=\mathrm{SiLU}\left({Conv1d}_{d_{\text{inner}},k=3}^{\text{depth}}\left(\text{u}\right)\right)\in {\mathbb{R}}^{B\times L\times d_{\text{inner}}}, \end{array}$$ (17)

where left-padding of 2 ensures strict causality (no access to future time steps).

#### 3.3.4 Regime-conditioned SSM parameter projections

This is the **key novelty** of the RC-Mamba block. After causal convolution, the convolved features are concatenated with the regime posterior *before* projecting to the SSM parameters Δ, **B**, **C** (Equation 18):


$$\begin{array}{l} {\text{z}}_{g,t}=\left[{\text{u}}_{\text{conv},t},\gamma_{t}\right]\in {\mathbb{R}}^{d_{\text{inner}}+K}, \end{array}$$ (18)

$$\begin{array}{l} \Delta_{t}=\mathrm{softplus}\left({\text{W}}_{\Delta }{\text{z}}_{g,t}+{\text{b}}_{\Delta }\right)+\varepsilon ,\;\varepsilon =10^{-4}, \end{array}$$ (19)

$$\begin{array}{l} {\text{B}}_{t}={\text{W}}_{B}{\text{z}}_{g,t}+{\text{b}}_{B}, \end{array}$$ (20)

$$\begin{array}{l} {\text{C}}_{t}={\text{W}}_{C}{\text{z}}_{g,t}+{\text{b}}_{C}, \end{array}$$ (21)

where ${\text{W}}_{\Delta },{\text{W}}_{B},{\text{W}}_{C}\in {\mathbb{R}}^{d_{\text{state}}\times \left(d_{\text{inner}}+K\right)}$ and *d*_inner_ + *K* = 32+3 = 35 in the selected configuration.

Under the full conditioning mode, all three projections (Equations 19–21) receive γ_*t*_. Consequently, the discretisation step size Δ_*t*_ (controlling temporal resolution), the input mixing matrix **B**_*t*_ (controlling what enters the state), and the output readout matrix **C**_*t*_ (controlling what is emitted) are all explicitly modulated by the current regime state.

#### 3.3.5 Stability of the state transition

The diagonal transition parameter is stored in log-space and negated through a softplus to guarantee non-positive values (Equation 22):


$$\begin{array}{l} \text{A}=-\mathrm{softplus}\left({\text{A}}_{\log}\right),\;{\text{A}}_{\log}\in {\mathbb{R}}^{d_{\text{state}}}\text{(learnable)}. \end{array}$$ (22)

Since softplus(*x*) = log(1 + *e*^*x*^) ≥ 0, we have **A** ≤ 0 always, and ${\bar{\text{A}}}_{t}=\exp\left(\Delta_{t}⊙\text{A}\right)\in \left(0,1\right]$, which guarantees that the recurrence cannot diverge.

#### 3.3.6 Zero-order hold discretisation

Discretisation via zero-order hold (ZOH) at the time-varying step size Δ_*t*_ yields (Equations 23, 24):


$$\begin{array}{l} {\bar{A}}_{t}=\exp(\Delta_{t}⊙A)\in {\left(0,1\right]}^{d_{\text{state}}}, \end{array}$$ (23)

$$\begin{array}{l} {\bar{\text{B}}}_{t}=\Delta_{t}⊙{\text{B}}_{t}\in {\mathbb{R}}^{d_{\text{state}}}. \end{array}$$ (24)

The regime-dependent Δ_*t*_ thus controls the effective “clock speed” of the SSM: in stress regimes, the model may learn shorter step sizes for finer temporal resolution; in calm periods, longer steps suffice for smoother dynamics.

#### 3.3.7 SSM recurrence (sequential scan)

With **h**_0_ = **0**, the sequential scan proceeds for *t* = 1, …, *L* (Equations 25, 26):


$$\begin{array}{l} {\text{h}}_{t}={\bar{\text{A}}}_{t}⊙{\text{h}}_{t-1}+{\bar{\text{B}}}_{t}⊙{\text{x}}_{\text{state},t}, \end{array}$$ (25)

$$\begin{array}{l} {\text{y}}_{\text{state},t}={\text{C}}_{t}⊙{\text{h}}_{t}, \end{array}$$ (26)

where **x**_state_ = **W**_*x*_ **u**_conv_ with ${\text{W}}_{x}\in {\mathbb{R}}^{d_{\text{state}}\times d_{\text{inner}}}$. All operations are element-wise (diagonal SSM), yielding *O*(*L* · *d*_state_) complexity per forward pass.

#### 3.3.8 D-skip, output projection, and residual connection

The state-space output is projected back to the expansion dimension and combined with a learnable direct-feedthrough skip (Equation 27):


$$\begin{array}{l} {\text{y}}_{\text{inner}}={\text{W}}_{\text{so}}{\text{y}}_{\text{state}}+\text{D}⊙{\text{u}}_{\text{conv}}, \end{array}$$ (27)

where $\text{D}\in {\mathbb{R}}^{d_{\text{inner}}}$ is a learnable parameter initialized to ones, and ${\text{W}}_{\text{so}}\in {\mathbb{R}}^{d_{\text{inner}}\times d_{\text{state}}}$.

The final block output follows the pre-normalization residual pattern (Equation 28):


$$\begin{array}{l} \text{output}=\text{x}+\mathrm{Dropout}\left({\text{W}}_{\text{out}}{\text{y}}_{\text{inner}}\right), \end{array}$$ (28)

where ${\text{W}}_{\text{out}}\in {\mathbb{R}}^{d_{\text{model}}\times d_{\text{inner}}}$ and the residual skip is from the *raw input* **x** (before LayerNorm).

#### 3.3.9 Gate ablation

In standard Mamba, a SiLU gate **z** modulates the SSM output via **y**_gated_ = **y**_inner_ ⊙ SiLU(**z**). In the selected configuration, this gate is **disabled**: **y**_gated_ = **y**_inner_.

*Empirical Observation* 1. When regime posteriors γ_*t*_ condition all three SSM parameter projections (Δ, **B**, **C**), the SiLU gate becomes a tunable knob in a calibration-vs.-accuracy trade-off rather than a strictly necessary component. In the matched ablation of Section 4.7, disabling the gate reduces the block parameter count, but the evidence does not support the stronger claim that it also improves accuracy or calibration: the gate-enabled variant attains marginally lower CRPS (by 0.35 %), coverage closer to the nominal level and a better Winkler_80_ score.

The gate-off configuration reported throughout this paper is the one pre-selected before the ablations were run. We retain it rather than re-selecting an architecture on the basis of ablation results, and we treat the gate as a parameter-efficiency choice whose accuracy direction is not established at this effect size. Because the RC-Mamba use case targets distributional accuracy and risk management rather than point alpha, the selected configuration is gate-off. We frame this finding as an *empirical observation* rather than a theorem: we do not prove gate redundancy in closed form, and the trade-off is contingent on the specific architectural configuration and the choice of objective (calibration sharpness vs. central-tendency error).

#### 3.3.10 Conditioning mode summary

Supplementary Table S14 summarizes the three available conditioning modes. The selected configuration uses full.

### 3.4 Variable selection and context construction

The Gated Residual Network (GRN) and Variable Selection Network (VSN) are adopted without modification from (Lim et al., 2021): the GRN applies a context-conditioned ELU projection followed by a gated linear unit and a layer-normalized residual connection, and the VSN combines *N* independently projected variables using learned softmax weights that are directly interpretable as variable-importance scores. For the historical VSN *N* = 30, and for the future VSN *N* = 3. Their full formulations are reproduced in Supplementary Section S5.2 and illustrated in Supplementary Figures S3a,b.

#### 3.4.1 Context vector construction

The context vector that conditions both VSNs concatenates the regime posteriors with the time-expanded static embedding (Equation 29):


$$\begin{array}{l} {\text{c}}_{t}=\left[\gamma_{t},\text{e}\right]\in {\mathbb{R}}^{K+d_{s}},\;t\in \left\{1,\ldots ,T\right\}\cup \left\{T+1,\ldots ,T+H\right\}, \end{array}$$ (29)

where $\text{e}\in {\mathbb{R}}^{d_{s}}$ is the static embedding (see Section 3.5). The regime posteriors inject a time-varying structural prior into the selection process, enabling the VSN to weight variables differently across market regimes.

#### 3.4.2 Temporal sequence assembly

The outputs of the historical and future VSNs are concatenated along the time axis to form the input to the RC-Mamba block (Equations 30, 31):


$$\begin{array}{l} \text{full\_seq}=\left[{\text{v}}_{\text{hist}},{\text{v}}_{\text{futr}}\right]\in {\mathbb{R}}^{B\times \left(T+H\right)\times d_{\text{model}}}, \end{array}$$ (30)

$$\begin{array}{l} \gamma_{\text{full}}=\left[\gamma_{\text{hist}},\gamma_{\text{future}}\right]\in {\mathbb{R}}^{B\times \left(T+H\right)\times K}. \end{array}$$ (31)

The concatenation preserves temporal ordering: historical steps 1, …, *T* precede future steps *T* + 1, …, *T* + *H*. The Mamba block's causal convolution and SSM recurrence process the combined sequence in strict left-to-right order.

### 3.5 Hierarchical FiLM embedding

Each time series *i* is identified by a ticker ID and a GICS sector ID. The static embedding is constructed hierarchically (Equations 32–34):


$$\begin{array}{l} {\text{e}}_{\text{sector}}={\mathrm{Embed}}_{\text{sector}}\left(\text{sector\_id}\right)\in {\mathbb{R}}^{d_{s}}, \end{array}$$ (32)

$$\begin{array}{l} {\text{e}}_{\text{ticker}}={\mathrm{Embed}}_{\text{ticker}}\left(\text{ticker\_id}\right)\in {\mathbb{R}}^{d_{s}},\text{ init }N\left(0,0.01^{2}\right), \end{array}$$ (33)

$$\begin{array}{l} \text{e}={\text{e}}_{\text{sector}}+{\text{e}}_{\text{ticker}}\in {\mathbb{R}}^{d_{s}},\;d_{s}=16. \end{array}$$ (34)

The sector embedding captures shared market-regime patterns across assets within the same sector, while the ticker residual (initialized near zero) learns asset-specific deviations.

The static embedding **e** is consumed at two points in the architecture:

1. As part of the context vector **c**_*t*_ = [γ_*t*_, **e**] that conditions both VSNs (see Equation 29).
2. In the Ticker-Conditioned Head, which applies FiLM modulation and a bottleneck adapter before the distribution head.

#### 3.5.1 Ticker-conditioned head (FiLM + adapter)

After the post-Mamba GRN processes the future-horizon slice ${\text{h}}^{\prime }\in {\mathbb{R}}^{B\times H\times d_{\text{model}}}$ and the regime projection $\text{r}={\text{W}}_{r}\gamma_{\text{future}}+{\text{b}}_{r}\in {\mathbb{R}}^{B\times H\times d_{\text{model}}}$ are concatenated into $\tilde{\text{h}}=\left[{\text{h}}^{\prime },\text{r}\right]\in {\mathbb{R}}^{B\times H\times 2d_{\text{model}}}$, the Ticker-Conditioned Head adapts the shared backbone features per-asset (Equations 35–37):

##### 3.5.1.1 FiLM modulation


$$\begin{array}{l} \gamma_{\text{FiLM}}={\text{W}}_{\gamma }\text{e},\;\beta_{\text{FiLM}}={\text{W}}_{\beta }\text{e}, \end{array}$$ (35)

$$\begin{array}{l} {\tilde{\text{h}}}^{\prime }=\gamma_{\text{FiLM}}⊙\tilde{\text{h}}+\beta_{\text{FiLM}}, \end{array}$$ (36)

where ${\text{W}}_{\gamma },{\text{W}}_{\beta }\in {\mathbb{R}}^{2d_{\text{model}}\times d_{s}}$ are initialized such that γ_FiLM_ ≈ **1** and **β**_FiLM_ ≈ **0**, so the layer begins as a passthrough.

##### 3.5.1.2 Bottleneck adapter


$$\begin{array}{l} {\tilde{\text{h}}}^{\prime \prime }={\tilde{\text{h}}}^{\prime }+{\text{W}}_{2}\mathrm{GELU}\left({\text{W}}_{1}\left[{\tilde{\text{h}}}^{\prime },\text{e}\right]+{\text{b}}_{1}\right), \end{array}$$ (37)

where the adapter bottleneck uses ratio 1/4, i.e., *d*_bn_ = ⌊2*d*_model_/4⌋. The residual structure ensures that the adapter can be learned incrementally without disrupting the shared representation.

### 3.6 Regime uncertainty gate

The Regime Uncertainty Gate (RUG) adaptively modulates the predictive scale parameter based on the model's confidence in the current regime assignment. When the posterior is uncertain (high entropy, transitioning between regimes), the gate widens the predictive distribution; when confident, it narrows it. The RUG architecture is shown in Supplementary Figure S3c.

#### 3.6.1 Uncertainty feature extraction

Three complementary diagnostics are extracted from the posterior γ ∈ ℝ^*B*×*H*×*K*^ (Equations 38–40):


$$\begin{array}{l} f_{1}=\frac{H\left(\gamma_{t}\right)}{\log K}=\frac{-\sum_{k}\gamma_{t,k}\log\gamma_{t,k}}{\log K}\in \left[0,1\right], \end{array}$$ (38)

$$\begin{array}{l} f_{2}=\underset{k}{\max}\gamma_{t,k}\in \left[1/K,1\right], \end{array}$$ (39)

$$\begin{array}{l} f_{3}=1-\cos\left(\gamma_{t},\gamma_{t-1}\right)\in \left[0,2\right], \end{array}$$ (40)

where *f*_1_ captures posterior *spread* (normalized entropy), *f*_2_ captures *peakedness* (max posterior probability), and *f*_3_ captures temporal *instability* of the regime assignment (cosine distance between consecutive posteriors, using γ_hist_ as reference for the transition uncertainty computation).

#### 3.6.2 Bounded gate network

The three features **f** = [*f*_1_, *f*_2_, *f*_3_] are mapped through a two-layer MLP to a bounded multiplicative scale factor (Equations 41, 42):


$$\begin{array}{l} \text{raw}={\text{W}}_{2}\mathrm{GELU}\left({\text{W}}_{1}\text{f}+{\text{b}}_{1}\right)+{\text{b}}_{2}, \end{array}$$ (41)

$$\begin{array}{l} s=s_{\min}+\left(s_{\max}-s_{\min}\right)\sigma \left(\text{raw}\right),\;s\in \left[0.5,2.0\right], \end{array}$$ (42)

where ${\text{W}}_{1}\in {\mathbb{R}}^{3\times 32}$, ${\text{W}}_{2}\in {\mathbb{R}}^{32\times 1}$, *s*_min_ = 0.5, and *s*_max_ = 2.0.

The modulated predictive scale is Equation 43:


$$\begin{array}{l} {\hat{\sigma }}_{\text{pred}}=s\cdot \sigma_{\text{pred}}. \end{array}$$ (43)

The bounded sigmoid activation constrains *s* to [*s*_min_, *s*_max_], preventing degenerate predictions while permitting up to 2 × interval widening during high-uncertainty regimes and 0.5 × tightening during confident single-regime periods.

### 3.7 Student-*t* distribution head

The final representation ${\tilde{\text{h}}}^{\prime \prime }\in {\mathbb{R}}^{B\times H\times 2d_{\text{model}}}$ from the Ticker-Conditioned Head is mapped to three Student-*t* distribution parameters via a single linear projection (Equation 44):


$$\begin{array}{l} \left[\hat{\nu },\hat{\mu },ŝ\right]={\text{W}}_{\text{head}}{\tilde{\text{h}}}^{\prime \prime }+{\text{b}}_{\text{head}},\;{\text{W}}_{\text{head}}\in {\mathbb{R}}^{3\times 2d_{\text{model}}}, \end{array}$$ (44)

with constrained activations ensuring valid distribution parameters (Equations 45–47):


$$\begin{array}{l} \nu =3+\mathrm{softplus}\left(\hat{\nu }\right), \end{array}$$ (45)

$$\begin{array}{l} \mu =\hat{\mu }, \end{array}$$ (46)

$$\begin{array}{l} \hat{\sigma }=\mathrm{softplus}\left(ŝ\right)+10^{-6}. \end{array}$$ (47)

The constraint ν > 3 ensures finite variance for the Student-*t* predictive distribution (the standard moment conditions are ν > 2 for finite variance and ν > 4 for finite kurtosis), which is more than sufficient for the first-moment regularity required by the CRPS formulation used in Stage 4 training. The Regime Uncertainty Gate then modulates the base scale (Equation 48):


$$\begin{array}{l} \sigma =\sigma_{u}\cdot \hat{\sigma },\;\sigma_{u}\in \left[0.5,2.0\right]. \end{array}$$ (48)

At inference time, *S* = 1000 Monte Carlo samples are drawn from Student-*t*(ν, μ, σ) to construct empirical quantile forecasts at levels {0.10, 0.50, 0.90}, yielding point forecasts (median) and 80% prediction intervals.

### 3.8 Regime-conditioned interpretability framework

A core contribution of RC-Mamba is its *regime-conditioned interpretability*: the same HMM posteriors γ that condition the Mamba recurrence also stratify *post-hoc* feature attributions, revealing how the model's reliance on individual covariates changes across market regimes. The framework combines three complementary pillars: intrinsic Variable Selection Network (VSN) weights, model-agnostic SHAP attributions (Lundberg and Lee, 2017), and HMM regime diagnostics (Supplementary Figure S4).

#### 3.8.1 Prediction function for grouped permutation attribution

We define a scalar prediction function for grouped permutation attribution by averaging the forecast median over the 30-step horizon. Concretely, we construct a wrapper that flattens the historical exogenous tensor ${\text{X}}_{\text{hist}}\in {\mathbb{R}}^{T\times V}$ into a single vector ${\text{x}}_{\text{flat}}\in {\mathbb{R}}^{TV}$, reshapes it back to [1, *T, V*], injects the stored ancillary inputs (future covariates, macro observables, ticker and sector identifiers), and returns a scalar prediction (Equation 49):


$$\begin{array}{l} f\left({\text{x}}_{\text{flat}}\right)=\frac{1}{H}\overset{H}{\underset{h=1}{\sum }}{\hat{q}}_{50,h}, \end{array}$$ (49)

where ${\hat{q}}_{50,h}$ is the median forecast (Student-*t* location parameter) at horizon *h*. Ancillary inputs are stored from a reference batch and broadcast-expanded to the current perturbation batch size, ensuring SHAP perturbations affect only the *V* = 30 historical features.

#### 3.8.2 Grouped permutation SHAP

Attributions are computed with grouped permutation SHAP. Each of the *V* = 30 historical features is treated as a single player carrying all *T* = 24 of its lags, so the coalition space is 2^*V*^ rather than 2^*TV*^ and the estimator is exact in expectation without a coalition-weighted regression. For each evaluation sample we draw *P* = 64 random feature orderings in antithetic pairs—every permutation is used together with its reverse, which cancels the leading-order sampling error—and accumulate the marginal contribution of each feature as it is revealed (Equation 50):


$$\begin{array}{l} \phi_{j}^{\left(n\right)}=\frac{1}{P}\overset{P}{\underset{p=1}{\sum }}\left[f\left({\text{x}}_{S_{p,j}\cup \left\{j\right\}}^{\left(n\right)}\right)-f\left({\text{x}}_{S_{p,j}}^{\left(n\right)}\right)\right], \end{array}$$ (50)

where $S_{p,j}$ is the set of features preceding *j* in permutation *p* and masked features are set to the baseline. The baseline is zero in the training-fold robust-MAD coordinates of Equation 63, that is the training-period median, so a masked feature contributes its typical in-sample level rather than an out-of-distribution value. Local accuracy $\underset{j}{\sum }\phi_{j}^{\left(n\right)}=f\left({\text{x}}^{\left(n\right)}\right)-f\left(0\right)$ is verified for every sample; the maximum violation over the reported analysis is 3.14 × 10^−9^.

The *global importance* of feature *j* (Equation 51) is then


$$\begin{array}{l} {\bar{S}}_{j}=\frac{1}{N}\overset{N}{\underset{n=1}{\sum }}\left|\phi_{j}^{\left(n\right)}\right|. \end{array}$$ (51)

Because a feature and all its lags form one player, the analysis does not resolve attribution across time within a feature; that is the price paid for an estimator whose local accuracy can be checked exactly at this sample size.

#### 3.8.3 Regime-grouped attribution

For each evaluation sample *n*, the *dominant regime* is assigned by averaging the HMM posterior over the lookback window and taking the argmax (Equation 52):


$$\begin{array}{l} k_{n}^{⋆}=\underset{j\in \left\{1,\ldots ,K\right\}}{\mathrm{argmax}}\frac{1}{T}\overset{T}{\underset{t=1}{\sum }}\gamma_{tj}^{\left(n\right)}. \end{array}$$ (52)

Let $R_{k}=\left\{n:k_{n}^{⋆}=k\right\}$ denote the index set of samples assigned to regime *k*. The *regime-conditioned mean absolute SHAP* for feature *i* under regime *k* (Equation 53) is


$$\begin{array}{l} {\bar{S}}_{i}^{\left(k\right)}=\frac{1}{\left|R_{k}\right|}\underset{n\in R_{k}}{\sum }\left|\phi_{i}^{\left(n\right)}\right|. \end{array}$$ (53)

Because each feature enters Equation 50 as a single grouped player carrying all *T* of its lags, $\phi_{i}^{\left(n\right)}$ is already one attribution per feature per sample and no average over *t* is taken here.

The *regime differential ratio* quantifies how much a feature's importance amplifies or attenuates under market stress compared with calm conditions (Equation 54):


$$\begin{array}{l} r_{i}=\frac{{\bar{S}}_{i}^{\left(\text{stress}\right)}}{{\bar{S}}_{i}^{\left(\text{calm}\right)}}, \end{array}$$ (54)

where *r*_*i*_ > 1 indicates stress amplification and *r*_*i*_ < 1 indicates attenuation. Features with ${\bar{S}}_{i}^{\left(\text{calm}\right)}<10^{-9}$ are excluded from ratio computation to avoid numerical artifacts.

#### 3.8.4 Ticker-grouped attribution

An analogous grouping by ticker identity yields asset-level profiles (Equation 55):


$$\begin{array}{l} {\bar{S}}_{i}^{\left(\tau \right)}=\frac{1}{\left|T_{\tau }\right|}\underset{n\in T_{\tau }}{\sum }\left|\phi_{i}^{\left(n\right)}\right|, \end{array}$$ (55)

where $T_{\tau }$ is the set of samples for ticker τ. This reveals whether distinct asset classes develop specialized factor dependencies without explicit class supervision.

#### 3.8.5 Intrinsic VSN importance

The Variable Selection Networks (Section 3.4) produce softmax attention weights *w*_*it*_ ∈ [0, 1] at each timestep. The global intrinsic importance of feature *i* (Equation 56) is


$$\begin{array}{l} G_{i}=\frac{1}{N}\overset{N}{\underset{n=1}{\sum }}{\bar{w}}_{in},\;{\bar{w}}_{in}=\frac{1}{T}\overset{T}{\underset{t=1}{\sum }}w_{it}^{\left(n\right)}. \end{array}$$ (56)

A regime-weighted variant uses the HMM posteriors as soft membership weights (Equation 57):


$$\begin{array}{l} G_{i}^{\left(k\right)}=\frac{\sum_{n=1}^{N}\gamma_{nk}{\bar{w}}_{in}}{\sum_{n=1}^{N}\gamma_{nk}+\varepsilon },\;\varepsilon =10^{-8}. \end{array}$$ (57)

### 3.9 Four-stage training protocol

Training proceeds through four stages with a deliberate *freeze–unfreeze–re-freeze* schedule for the HMM parameters. This curriculum ensures stable regime conditioning before end-to-end alignment, followed by calibration-focused fine-tuning. The total training budget is 11,450 gradient steps per fold.

Supplementary Figure S5 illustrates the four stages, showing which parameter groups are trainable at each stage and the loss functions driving optimisation.

#### 3.9.1 Stage 1: HMM pretrain (300 steps)

Only the HMM parameters {**μ**, **θ**^var^, **θ**^df^, **A**^raw^} are trainable; all forecasting network weights are frozen. The loss is Equation 58:


$$\begin{array}{l} L_{1}=L_{\text{NLL}}+\lambda_{\text{ent}}L_{\text{entropy}}+\lambda_{\text{st}}L_{\text{self}}+\lambda_{\text{sticky}}L_{\text{Dir}}, \end{array}$$ (58)

with λ_ent_ = 5, λ_st_ = 20, and target self-transition τ = 0.80. The optimizer is Adam with η = 10^−3^ and gradient clipping at 1.0.

A **collapse gate** monitors regime health: training is halted early if any regime exceeds 90% occupancy or 0.95 self-transition probability, preventing degenerate single-regime solutions.

*Rationale*. Isolating HMM training allows the regime detection module to learn meaningful state structures from macro observables without interference from forecast gradients. This provides the downstream Mamba block with informative regime conditioning signals from the very start of Stage 2.

#### 3.9.2 Stage 2: forecast network training (10,000 steps)

The HMM is *frozen* and the forecast network (Mamba block, VSNs, embeddings, GRNs, and distribution head) is trained on the negative log-likelihood of the Student-*t* output distribution (Equation 59):


$$\begin{array}{l} L_{2}=-\frac{1}{BH}\overset{B}{\underset{b=1}{\sum }}\overset{H}{\underset{h=1}{\sum }}\log p_{\text{Student-}t}\left(y_{b,h}|\nu_{b,h},\mu_{b,h},\sigma_{b,h}\right). \end{array}$$ (59)

The optimizer is AdamW (Loshchilov and Hutter, 2019) with η = 10^−3^, weight decay 10^−4^, and a cosine-annealing scheduler (50-step linear warm-up, minimum η = 10^−6^). Validation loss is evaluated every 100 steps with early stopping patience of 10.

*Rationale*. Freezing the HMM lets the forecasting network adapt to fixed regime signals, establishing a stable regime-conditioned representation before joint optimisation.

#### 3.9.3 Stage 3: joint fine-tuning (750 steps)

*All* parameters (both HMM and forecast network) are unfrozen for end-to-end alignment. The composite loss is Equation 60:


$$\begin{array}{l} L_{3}=L_{\text{forecast}}+\lambda_{\text{hmm}}L_{\text{NLL}}+\lambda_{\text{ent}}L_{\text{entropy}}\\ \;+\lambda_{\text{st}}L_{\text{self}}+\lambda_{\text{sticky}}L_{\text{Dir}}+\lambda_{L_{2}}L_{\text{residual}}, \end{array}$$ (60)

with λ_hmm_ = 0.1, λ_ent_ = 1, and λ_st_ = 5. The optimizer is AdamW with η ≈ 10^−4^ and weight decay 10^−4^.

A critical design choice is **separate gradient clipping**: HMM parameters use a tighter norm bound than the deep network, preventing gradient spikes from destabilizing the learned transition matrix. Early stopping is guided by the forecast loss only; the HMM regularization terms serve as training-only constraints.

*Rationale*. Joint fine-tuning allows the forecast loss to back-propagate through the regime posteriors into the HMM transition dynamics, enabling end-to-end alignment between regime detection and forecasting objectives.

#### 3.9.4 Stage 4: CRPS calibration fine-tuning (400 steps)

The HMM is *re-frozen* and the forecast head is fine-tuned with the Continuous Ranked Probability Score (CRPS; Equation 61), a strictly proper scoring rule (Gneiting and Raftery, 2007):


$$\begin{array}{l} \text{CRPS}\left(F,y\right)={𝔼}_{X\sim F}\left[\left|X-y\right|\right]-\frac{1}{2}{𝔼}_{X,X^{\prime }\sim F}\left[\left|X-X^{\prime }\right|\right], \end{array}$$ (61)

estimated via Monte Carlo sampling (Equation 62):


$$\begin{array}{l} \hat{\text{CRPS}}\approx \frac{1}{S}\overset{S}{\underset{i=1}{\sum }}|x_{i}-y|-\frac{1}{2S^{2}}\overset{S}{\underset{i=1}{\sum }}\overset{S}{\underset{j=1}{\sum }}|x_{i}-x_{j}|,\;S=200. \end{array}$$ (62)

The optimizer is AdamW with η ≈ 10^−5^, weight decay 10^−5^, and gradient clipping at 0.5.

An **RMSE guardrail** provides a safety net: if the post-finetune RMSE degrades by more than 15% relative to the pre-finetune checkpoint, all weights are rolled back.

*Rationale*. While the Student-*t* NLL (Stage 2) is a proper scoring rule, it can produce overly sharp predictions. CRPS directly optimizes the trade-off between sharpness and calibration, improving prediction interval quality. Re-freezing the HMM prevents CRPS gradients from destabilizing the learned regime structure.

#### 3.9.5 Training protocol summary

Supplementary Table S15 summarizes the hyperparameters of each stage.

The freeze–unfreeze–re-freeze cycle is central to the training design: it prevents severe interaction between the HMM's statistical estimation and the deep network's stochastic gradient dynamics, while still permitting end-to-end alignment during the joint fine-tuning stage.

### 3.10 Experimental setup and evaluation protocol

This section details the dataset, preprocessing pipeline, walk-forward validation protocol, baseline models, evaluation metrics, and implementation specifics used to evaluate RC-Mamba.

#### 3.10.1 Dataset

The evaluation universe comprises 18 US-listed Exchange-Traded Funds (ETFs) spanning five distinct asset classes, selected to provide broad coverage of global financial markets with heterogeneous return dynamics. Table 1 summarizes the full asset universe and sector assignments.

**Table 1:** ETF universe used for evaluation.

Asset class	Ticker	Description	Sector
US Equity	SPY	S&P 500 (large cap)	Equity
	VOO	S&P 500 (Vanguard)	Equity
	VIOO	S&P 600 (small cap)	Equity
	IVOO	S&P 400 (mid cap)	Equity
Fixed income	BND	US total bond market	Fixed income
	GOVT	US treasury bond	Fixed income
	IGOV	Intl government bond	Fixed income
	JNK	High-yield corporate	Fixed income
	VWOB	EM government bond	Fixed income
Commodity	GLD	Gold	Commodity
EM equity	EMXC	EM ex-China	Equity
	CEMB	EM corporate bond	Fixed income
Intl equity	EWG	Germany (MSCI)	Equity
	EWH	Hong Kong (MSCI)	Equity
	EWQ	France (MSCI)	Equity
	EWU	United Kingdom (MSCI)	Equity
	VGK	FTSE Europe	Equity
	FXI	China large cap	Equity

The 18 assets span five asset classes, ensuring that the model is tested on instruments with diverse return distributions, volatility regimes, and factor exposures.

The data span begins on 3 January 1994 and ends on 27 March 2026, covering 8,113 business days at the longest ETF coverage. Eight of the 18 ETFs (SPY, BND, GLD and the country/region trackers) have inception dates within that window and therefore contribute fewer windows to the early folds; the panel is unbalanced. Missing rows preceding an ETF's inception date are excluded at the data-loader stage by masking the loss to skip windows that do not have full *T* + *H* = 54 business days of historical context for that ticker. Within an asset's available history, no imputation is applied: the macro-financial and price series used as inputs are essentially complete after the publication-lag adjustment described in Section 3.10.3. The headline figure of 8,113 business days refers to the longest single-series calendar coverage in the raw data and macro-observable panel; it is distinct from the asset-level walk-forward training partitions in Supplementary Table S17, which are constructed after valid *T* + *H* = 54 window filtering across the ETF panel. It should therefore not be confused with the panel observation count *N* = 599, 400 of the revised test grid, which counts every per-(asset, fold, day, horizon) realization and which Section 4.2 reduces to 1, 110 synchronized forecast origins for statistical testing. The forecasting target is the daily log-return of closing prices: *y*_*t*_ = log(Close_*t*_/Close_*t*−1_). The model uses a lookback window of *T* = 24 business days and produces multi-step probabilistic forecasts over a horizon of *H* = 30 business days.

#### 3.10.2 Input features

The model ingests *V*_*h*_ = 30 historical exogenous features spanning price and volume statistics, technical indicators, macro-financial series and sentiment aggregates; Supplementary Table S16 lists all of them with their sources and publication lags, which are respected during walk-forward evaluation to prevent look-ahead bias. Three calendar features are used as known-future covariates (*V*_*f*_ = 3): day_of_week, week_of_year and month, supplied as raw integer encodings. The HMM operates on a separate observation vector ${\text{o}}_{t}\in {\mathbb{R}}^{3}$ comprising the CBOE Volatility Index (VIXCLS), the ICE BofA US High-Yield option-adjusted spread (BAMLH0A0HYM2) and the 10-year minus 2-year Treasury slope (DGS10−DGS2), chosen to capture equity risk, credit stress and term structure respectively. All three are published without lag. Supplementary Section S5.3 gives the full feature descriptions.

#### 3.10.3 Data preprocessing

Historical exogenous features are normalized using robust Median Absolute Deviation (MAD) scaling, fitted on the training partition of each fold only (Equation 63):


$$\begin{array}{l} \tilde{x}=\frac{x-\text{median}\left(x_{\text{train}}\right)}{1.4826\times \text{MAD}\left(x_{\text{train}}\right)}, \end{array}$$ (63)

where MAD(*x*) = median(|*x* − median(*x*)|) and the factor 1.4826 makes the estimator consistent for a Gaussian distribution. MAD scaling is preferred over *z*-score normalization because financial feature distributions are heavy-tailed, and MAD is robust to outliers (Rousseeuw and Croux, 1993). Future covariates (calendar features) are provided raw without normalization. Features with macro publication lags (Supplementary Table S16) are shifted forward by the appropriate number of business days before the walk-forward split, ensuring that no future macro data leaks into training or evaluation.

#### 3.10.4 Walk-forward validation protocol

We employ a 5-fold expanding-window walk-forward validation scheme following the purged time-series cross-validation framework of López de Prado López de Prado (2018), which replicates real-world deployment conditions where models are periodically retrained on all available historical data. Supplementary Table S17 summarizes the per-fold partition sizes.

The walk-forward protocol is governed by the following constraints:

- **Minimum training fraction:** 40% of total data (fold 0 is the smallest window).
- **Validation and test fractions:** 10% each per fold.
- **Common calendar cutoffs:** partition boundaries are defined on shared calendar dates rather than per-ticker positional indices, so that a single model trained across the panel cannot observe one asset's future while forecasting another asset's past. The five test blocks are the calendar years 2021–2025 and are verified disjoint.
- **Embargo:** 30 business days between training, validation and test partitions—equal to the maximum forecast horizon *H* = 30—with partition membership determined by target-end date rather than origin date. This removes the target-window overlap that a shorter gap permits, so no label observed in training can extend into a validation or test window. Fold-specific scaling statistics and the HMM are fitted on each fold's training period only.
- **Expanding window:** the training set grows monotonically with each successive fold; the validation and test windows slide forward in time.

Hyperparameter search is conducted exclusively on fold 0 (the smallest training window), with the best configuration applied identically across all five folds. This ensures that hyperparameter selection does not benefit from information in later folds.

#### 3.10.5 Hyperparameter selection

Hyperparameters are fixed a priori and are not tuned on the revised data: the values are those selected by a 9-configuration grid over learning rate, weight decay, HMM learning rate and HMM gradient clipping, run once on fold 0 and applied unchanged to every fold and every model family. No search is performed during the reported run, so no comparator is advantaged by fold-specific tuning. The grid and the selected values are given in Supplementary Table S18.

#### 3.10.6 Baseline models

RC-Mamba is compared against six baseline models spanning the spectrum of modern time-series forecasting architectures. All baselines share the same raw inputs, the same fold-local robust-MAD preprocessing, the same walk-forward splits, evaluation protocol and hardware, and the common optimisation ceiling of Section 3.10.13. What differs between them is the forecasting architecture and, where noted below, which of the available input pathways each reference design consumes.

#### 3.10.7 Vanilla TFT

The Vanilla Temporal Fusion Transformer (Lim et al., 2021) shares the same GRN/VSN variable selection, LSTM encoder–decoder, and ticker embedding architecture as RC-Mamba, but replaces the Mamba block with standard multi-head self-attention (*n*_*h*_ = 4 heads) and removes all regime conditioning components (HMM, RUG, regime posteriors). Architectural dimensions are identical: *d*_model_ = 64, *d*_var_ = 64, *d*_static_ = 16, dropout = 0.1. This baseline isolates the contribution of regime conditioning and the SSM temporal engine.

#### 3.10.8 DeepAR

DeepAR (Salinas et al., 2020) is an autoregressive LSTM-based model that generates probabilistic forecasts by teacher-forcing during training and median-feedback decoding at inference. The architecture uses a 2-layer LSTM with hidden dimension 128 and the same Student-*t* head as every other model, so its predictive distribution is directly comparable. Input features are the historical exogenous features and the HMM observables, concatenated at each time step; the future covariates and the ticker embedding are not part of the reference DeepAR interface and are not supplied to it.

#### 3.10.9 LSTM

A standard LSTM encoder-decoder baseline with 2 layers, hidden dimension 128, and dropout = 0.1. The encoder processes the concatenation of historical exogenous features and HMM observables; the final hidden state is projected through a linear layer to produce the full forecast horizon (*H* = 30), which is mapped to the shared Student-*t* head. Unlike DeepAR, no autoregressive decoding or teacher forcing is employed.

#### 3.10.10 N-BEATS

N-BEATS (Oreshkin et al., 2020) is a pure deep learning model based on fully-connected residual stacks with forward/backward basis expansions. We use three stacks of three blocks each (nine blocks total) with hidden dimension 256 and dropout = 0.1. The input is flattened from [*B, T, V*_*h*_] to [*B, T* × *V*_*h*_]. N-BEATS operates without exogenous future covariates by design, making it a test of whether pure endogenous modeling suffices under walk-forward distribution shift.

#### 3.10.11 S-Mamba

S-Mamba (Wang Z. et al., 2025) is included as a direct selective state-space competitor, so that regime conditioning can be assessed against an unconditioned selective-SSM backbone. It tokenises the variate dimension, applies bidirectional Mamba blocks across those variate tokens followed by a feed-forward temporal encoder, and projects from the pooled hidden state alone with no external conditioning pathway. We implement it as a probabilistic adaptation of that design: the reference tokenisation and bidirectional scan are preserved, and the same Student-*t* distribution head, the same fold-local robust-MAD preprocessing, the same folds and the same 11,450-update ceiling as RC-Mamba are attached to it. Following the reference architecture, it consumes the historical covariates and macro observables but not the future-covariate pathway or the ticker embedding, both of which are specific to the RC-Mamba and TFT input interface.

This comparison therefore benchmarks two complete architectures under matched preprocessing, output distribution, folds and optimisation ceiling. It does not isolate regime conditioning on its own: the two models also differ in tokenisation, scan direction, the balance of temporal against inter-variate processing, residual organization and the input pathways just noted. The matched within-backbone ablations of Section 4.7, which vary only the conditioning interface while holding the backbone fixed, provide the direct test of the selective-projection mechanism; S-Mamba establishes that the complete system is competitive against a published selective-SSM design.

#### 3.10.12 HMM-conditioned DeepAR

To separate the regime *signal* from the conditioning *interface*, we add a regime-aware DeepAR that receives exactly the same causal three-state HMM posterior γ_*t*_ used by RC-Mamba, concatenated to its input at every step, with the identical Student-*t* head and the same historical covariate and macro-observable inputs as the unconditioned DeepAR. The two models therefore see the same regime information and differ in how it enters the network: RC-Mamba conditions the selective projections (Δ_*t*_, **B**_*t*_, **C**_*t*_), whereas HMM-DeepAR conditions a recurrent probabilistic decoder through its inputs. As with the other recurrent comparators, the future covariates and ticker embedding are outside the reference DeepAR interface and are not supplied, so the contrast is between two regime-aware systems rather than a single isolated factor. It remains the closest available comparator for whether the selective-projection interface, rather than access to regime information as such, is what matters.

#### 3.10.13 Common training protocol

All neural models receive historical inputs transformed by the same fold-local robust-MAD scaler of Equation 63 and are trained under a common ceiling of 11,450 optimizer updates per fold. The shared optimizer settings are AdamW with weight decay 10^−4^, batch size 64 and gradient clipping at norm 1.0; the comparators use learning rate 10^−3^ and RC-Mamba uses 3 × 10^−3^ for its conditioned stages, decayed on the schedule of Section 3.9. Every model is fitted by minimizing the negative log-likelihood of the same Student-*t* predictive distribution, so the comparison is not confounded by a different training objective or a different output distribution.

Model-specific optimisation stages are retained only where they are structurally required, and they are allocations *within* the shared ceiling rather than extensions of it: RC-Mamba spends 300 updates on the HMM pretrain, 750 on joint fine-tuning and 400 on CRPS calibration, and HMM-DeepAR spends 300 on its own regime module. No architecture is constrained by a smaller optimisation ceiling than any other.

The realized training budget is determined exclusively by validation-based early stopping. Validation is evaluated at fixed intervals of 100 updates with a patience of 10 checks, and the checkpoint attaining the lowest validation loss is retained for evaluation. No model reaches the common ceiling: realized counts range from 1,100 to 7,750 updates. Realized update counts per fold are reported in Table 2 and validation-loss trajectories in Figure 3.

**Table 2:** Realized optimizer updates per fold under the common ceiling.

Model	Fold 0	Fold 1	Fold 2	Fold 3	Fold 4	Ceiling
RC-Mamba	5,550	6,550	5,450	6,950	7,750	11,450
VanillaTFT	1,300	5,600	1,400	1,300	7,100	11,450
DeepAR	2,400	5,400	1,700	2,800	1,600	11,450
S-Mamba	3,700	6,300	1,900	3,500	4,000	11,450
HMM-DeepAR	1,500	7,000	1,500	1,600	4,800	11,450
LSTM	4,300	4,700	1,800	5,800	5,100	11,450
N-BEATS	5,700	1,100	3,400	2,000	5,300	11,450

Every neural model is permitted the same 11,450-update budget; the realized number is determined by validation-based early stopping rather than by an asymmetric cap, which removes the training-budget confound raised in review.

**Figure 3:**
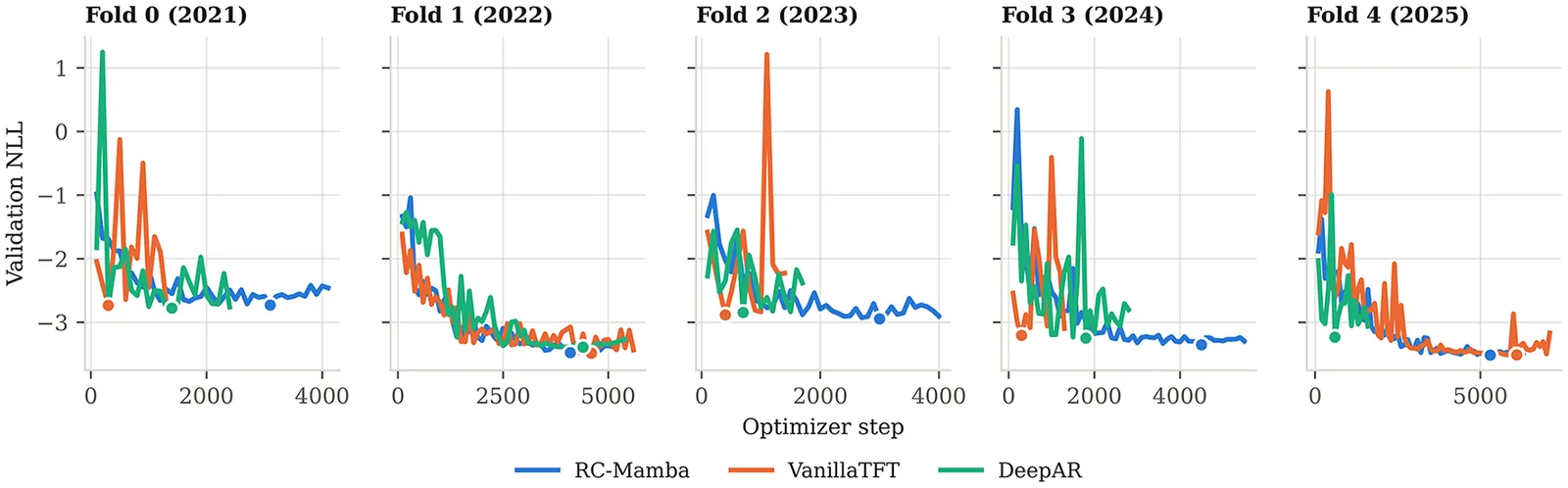
Validation negative log-likelihood against optimizer step for RC-Mamba, VanillaTFT, and DeepAR across all five folds under the common 11,450-update ceiling. Filled markers denote the selected checkpoint. Both baselines converge and plateau well before the ceiling, so their performance is not limited by the training budget.

#### 3.10.14 Evaluation metrics

All metrics are computed per asset and per fold, then aggregated as mean ± standard deviation across the five walk-forward folds. The primary metric is the Continuous Ranked Probability Score (CRPS), a strictly proper scoring rule that penalizes calibration error and lack of sharpness together (Gneiting and Raftery, 2007); for the Student-*t* predictive distribution it is estimated by Monte Carlo with *S* = 1, 000 draws at evaluation time. Point accuracy is reported as RMSE, MAE and directional accuracy, each computed from the median forecast. Interval quality is reported as PICP_80_ (empirical coverage), PINAW_80_ (normalized average width), and the Winkler_80_ score (Winkler, 1972), which penalizes under-coverage and width jointly and is therefore the interval metric we rank on; normalized conformal calibration (Romano et al., 2019) is applied per ticker and fold at the {50, 80, 90}% levels as an additional calibration check. Formal definitions of every metric are given in Supplementary Section S5.1.

#### 3.10.15 Statistical significance

Pairwise model comparisons are assessed at the level of the forecast origin rather than the individual prediction cell. Per-cell CRPS is first averaged over the 18 assets and the 30 horizon steps belonging to each origin, which reduces the 599, 400 overlapping cells of the revised test grid to 1, 110 synchronized fold–origin units and avoids treating horizons from one origin, or adjacent overlapping origins, as independent evidence. Confidence intervals and *p*-values are obtained from a moving-block bootstrap over each fold's ordered origin sequence (block length 30 origins, 2,000 replications), which preserves serial dependence within a fold and the contemporaneous cross-sectional dependence between assets on a shared date. We test *H*_0_: 𝔼[CRPS_*A*_ − CRPS_*B*_] = 0 at α = 0.05 and apply a Holm correction across the reported family of comparisons. A Diebold–Mariano test (Diebold and Mariano, 1995) with Newey–West HAC variance estimation at lag 29 is retained as a secondary check; where the two disagree we report the more conservative bootstrap result.

#### 3.10.16 Implementation and reproducibility

The selected RC-Mamba configuration is given in Supplementary Table S19 and the stage-wise training hyperparameters in Supplementary Table S15. All experiments run on a single NVIDIA GeForce RTX 3060 Laptop GPU (6 GB VRAM) in PyTorch, using a custom codebase with no external forecasting library; measured training and inference cost is reported in Section 4.9. Every random number generator is seeded with a fixed value of 42, the full configuration is serialized to YAML, and the fold splits, scaler statistics and HMM parameters are logged per fold, so the reported results reproduce exactly. Supplementary Section S5.4 gives the complete implementation and reproducibility detail.

## 4 Results

We evaluate RC-Mamba against six comparators under the walk-forward protocol of Section 3.10, reporting aggregate accuracy, statistical significance, per-fold stability, calibration, HMM regime validity, and ablation sensitivity.

### 4.1 Main results

Table 3 summarizes the 5-fold walk-forward performance of all seven models. RC-Mamba achieves the best score on CRPS (0.00532), MAE (0.00716), RMSE (0.01048), PINAW_80_ (0.024) and Winkler score (0.0364). HMM-DeepAR edges ahead on directional accuracy (51.3 % vs. 50.7 %), and LSTM's PICP_80_ is marginally closer to the nominal level (78.3 % vs. 78.1 %) while being far worse on every distributional and point metric; both margins are within sampling noise. Supplementary Figure S6 visualizes these results as dot-whisker plots, separating point-accuracy metrics (left) from calibration metrics (right) for clarity.

**Table 3:** Main leaderboard under the revised leakage-controlled protocol: mean ± standard deviation across five calendar-aligned walk-forward folds (test years 2021–2025).

Model	CRPS	MAE	RMSE	Dir %	PICP_80_	PINAW_80_	Winkler_80_
**RC-Mamba**	**0.00532** **±** **0.0013**	**0.00716** **±** **0.0018**	**0.01048** **±** **0.0024**	50.7	0.781 ± 0.119	**0.024**	**0.0364**
VanillaTFT	0.00558 ± 0.0011	0.00723 ± 0.0017	0.01052 ± 0.0024	49.1	0.869 ± 0.097	0.030	0.0402
DeepAR	0.00584 ± 0.0012	0.00761 ± 0.0018	0.01111 ± 0.0023	48.5	0.889 ± 0.032	0.033	0.0418
S-Mamba	0.00628 ± 0.0009	0.00816 ± 0.0014	0.01135 ± 0.0020	49.2	0.891 ± 0.077	0.036	0.0454
HMM-DeepAR	0.00653 ± 0.0015	0.00832 ± 0.0015	0.01170 ± 0.0018	**51.3**	0.828 ± 0.214	0.033	0.0491
LSTM	0.01260 ± 0.0146	0.00977 ± 0.0051	0.01398 ± 0.0069	49.3	**0.783** **±** **0.116**	0.104	0.1208
N-BEATS	0.10748 ± 0.1249	0.05765 ± 0.1000	0.10144 ± 0.1513	49.2	0.502 ± 0.317	0.736	1.1460

All models share robust-MAD preprocessing, a common ceiling of 11,450 optimizer updates with validation-based early stopping, and a 30-business-day origin embargo equal to the maximum forecast horizon. Bold indicates best per metric; PICP_80_ is judged by absolute distance from the nominal 80 % level.

Against the nearest competitor (VanillaTFT), RC-Mamba reduces CRPS by 4.7%, MAE by 1.0% and RMSE by 0.4%; the margin is widest on CRPS because that metric scores the whole predictive distribution rather than its center alone. The gap widens against DeepAR (8.9% CRPS, 5.7% RMSE) and LSTM (57.8% CRPS). Every percentage in this subsection is a reduction expressed relative to the comparator's own score, which is the convention used throughout the aggregate leaderboard; the relative column of Table 4 normalizes by RC-Mamba instead and is therefore numerically larger for the same underlying difference. Improvements of this magnitude are what the low signal-to-noise ratio of daily log-return prediction admits. They are measurable gains in statistical forecast quality; whether they translate into risk-adjusted economic value is a separate question, and the backtest of Section 4.11 does not establish a significant one.

**Table 4:** Dependence-aware pairwise comparison against RC-Mamba.

Comparison	Mean ΔCRPS	95 % CI	Rel. %	*p*_Holm_
RC-Mamba vs. VanillaTFT	−0.000262	[−0.000308, −0.000231]	4.93	0.006
RC-Mamba vs. DeepAR	−0.000518	[−0.000575, −0.000467]	9.74	0.006
RC-Mamba vs. S-Mamba	−0.000955	[−0.001021, −0.000901]	17.96	0.006
RC-Mamba vs. HMM-DeepAR	−0.001206	[−0.001343, −0.001037]	22.66	0.006
RC-Mamba vs. LSTM	−0.007275	[−0.008648, −0.006021]	136.71	0.006
RC-Mamba vs. N-BEATS	−0.102162	[−0.115918, −0.087271]	1919.77	0.006

Per-cell CRPS is first averaged over the 18 assets and 30 horizon steps within each forecast origin, giving 1,110 synchronized fold–origin observations rather than treating overlapping horizons as independent. Confidence intervals and *p*-values come from a moving-block bootstrap (block length 30 origins, 2,000 replications) that preserves serial and cross-sectional dependence. *p*-values are Holm-adjusted across the 6 comparisons reported here. Negative mean differences favor RC-Mamba. **Rel. %** is the comparator's excess over RC-Mamba, $\left({\bar{L}}_{\text{comp}}-{\bar{L}}_{\text{RC}}\right)/{\bar{L}}_{\text{RC}}$, computed as a ratio of means over the 1,110 origins; it is normalized by RC-Mamba and is therefore larger than the comparator-normalized reductions quoted in the abstract and in Section 4.1 for the same underlying difference. The adjusted *p*-values are identical across rows because every comparison attains the two-sided resolution floor of a 2,000-replication bootstrap, 2/(2,000 + 1): no replication reverses the sign of the mean difference, so the floor rather than the effect size determines the value, and Holm multiplies it by 6.

### 4.2 Statistical significance

To confirm that the observed improvements are not artifacts of fold sampling or of dependence between overlapping forecast windows, we compare models at the level of the forecast origin. Averaging per-cell CRPS within each origin over the 18 assets and 30 horizon steps yields 1, 110 synchronized fold–origin observations, and inference uses a moving-block bootstrap that respects both serial and cross-sectional dependence (Section 3.10.15). This is a substantially more conservative procedure than a pooled test that treats the 599, 400 individual forecast cells as independent, which would overstate the effective sample size. Table 4 reports every comparison against RC-Mamba, and Figure 4 presents the same estimates with their bootstrap intervals.

**Figure 4:**
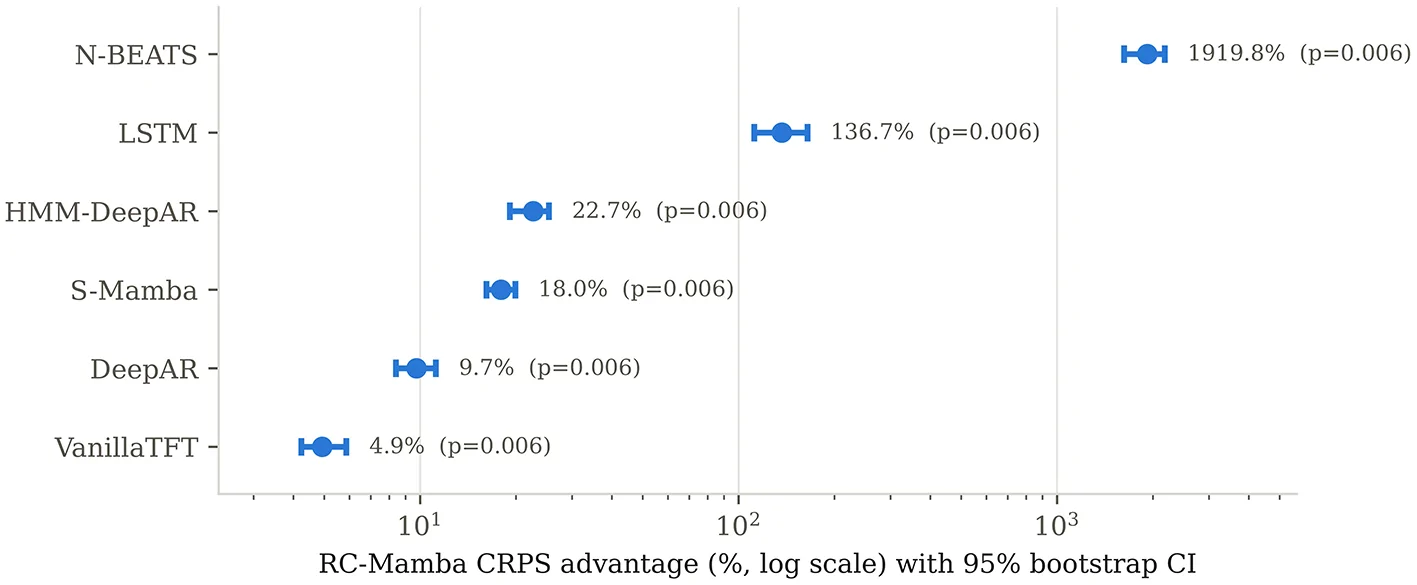
Dependence-aware pairwise comparison against RC-Mamba. Points show RC-Mamba's percentage CRPS advantage over each comparator on a logarithmic axis; bars are 95 % moving-block bootstrap intervals computed over 1,110 synchronized forecast origins, so overlapping horizons are never treated as independent. All six comparisons remain significant after Holm correction across the reported family.

All six comparisons remain significant after Holm correction over the retained family (*p*_Holm_ = 0.006), and the ranking on mean CRPS is strictly ordered: RC-Mamba ≻ VanillaTFT ≻ DeepAR ≻ S-Mamba ≻ HMM-DeepAR ≻ LSTM ≻ N-BEATS. The two comparisons that speak most directly to the mechanism are S-Mamba, an unconditioned selective-SSM backbone, and HMM-DeepAR, which receives the identical posterior through its inputs; RC-Mamba leads both. A secondary HAC-adjusted Diebold-Mariano test agrees with the bootstrap on every retained comparison (Supplementary Table S10, Supplementary Section S6.1). We note that the ordering is a ranking of complete systems: the matched no-regime control of Section 4.7 is not part of this family, and over five folds it is not separated from RC-Mamba at the origin level.

### 4.3 Per-fold stability analysis

Walk-forward evaluation partitions the data into five chronologically expanding folds, each testing on a distinct market period. Supplementary Table S20 reports CRPS and PICP_80_ per fold; Supplementary Figure S7 visualizes these trajectories, and Supplementary Tables S1–S5 give the complete per-fold metrics for all seven models.

Three stability findings emerge:

1. **RC-Mamba ranks first on CRPS in four of the five folds**. The exception is fold 1, the hardest window in the design, where the Vanilla TFT is ahead by 0.63% (0.00762 against 0.00767). RC-Mamba is the only model that is never worse than second in any fold. Its cross-fold CRPS standard deviation is 0.00133, which is *not* the lowest in the panel—S-Mamba (0.00093), the Vanilla TFT (0.00114) and DeepAR (0.00119) are all tighter, at uniformly worse mean CRPS. Low dispersion is therefore not by itself evidence of quality here, and we do not claim stability as an advantage over the competitive baselines.
2. **Fold 1 (test year 2022) is the hardest window**. Every one of the five competitive models records its worst CRPS in fold 1, at 1.3 to 1.6 times its mean over the remaining folds, and coverage falls furthest from nominal there (RC-Mamba 60.9%, HMM-DeepAR 44.6%). This is the window on which the matched ablations of Section 4.7 are run.
3. **The two weakest models fail in a different fold and for different reasons**. N-BEATS diverges in folds 1 and 2 (CRPS 0.2223 and 0.2633 against 0.00505 in its best fold, a 52× spread) with coverage collapsing to 14.8% in fold 1; as a pure basis-expansion model it cannot observe the macro covariates that signal those regime shifts. The LSTM fails in fold 2 instead (CRPS 0.0384, 8.2× its best fold), and does so by over-dispersing rather than under-covering: its PICP_80_ rises to 95.4% while PINAW_80_ reaches 0.416. The two failures therefore share no common signature and should not be treated as a single failure mode.

### 4.4 Calibration analysis

A well-calibrated probabilistic forecast should achieve empirical coverage close to the nominal level while maintaining *sharp* (narrow) intervals (Gneiting and Raftery, 2007). Table 5 compares calibration metrics, and Figure 5 visualizes the PICP–PINAW trade-off.

**Table 5:** Calibration at the 80 % nominal level under the revised protocol.

Model	PICP_80_ (%)	Dev.	PINAW_80_	Winkler_80_	Calib. err.
**RC-Mamba**	78.11	−1.89	**0.0235**	**0.0364**	**0.0400**
VanillaTFT	86.88	+6.88	0.0295	0.0402	0.0559
DeepAR	88.91	+8.91	0.0326	0.0418	0.0587
S-Mamba	89.06	+9.06	0.0361	0.0454	0.0724
HMM-DeepAR	82.81	+2.81	0.0333	0.0491	0.1084
LSTM	**78.30**	−1.70	0.1037	0.1208	0.0608
N-BEATS	50.20	−29.80	0.7360	1.1460	0.1362

PICP_80_ is the empirical coverage of the nominal 80 % interval and Dev. its signed deviation from 80 %; PINAW_80_ is the normalized interval width. Winkler_80_ is the proper interval score that penalizes width and miscoverage jointly, and is therefore the primary calibration criterion. Bold indicates the best value per metric, with PICP_80_ (and hence Dev.) judged by absolute distance from the nominal 80 % level; the bold row label marks the proposed model. Calib. err. is the normalized conformal calibration error of Section 3.10.14, averaged over the 50, 80, 90 % nominal levels.

**Figure 5:**
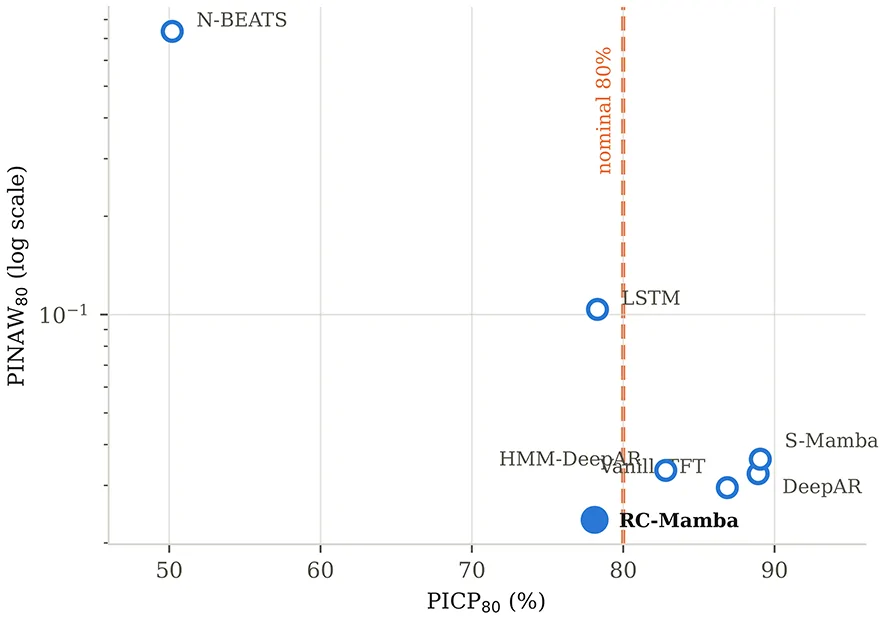
PICP_80_ vs. PINAW_80_ trade-off. The dashed orange line marks the nominal 80% target; models toward the lower right — high coverage with narrow intervals — are the best calibrated. DeepAR's PICP_80_ (0.889) exceeds the nominal target, achieved through 39% wider intervals (PINAW = 0.033 vs. 0.024) — i.e. DeepAR is *over-dispersed*. Under the proper scoring rule Winkler_80_, RC-Mamba (0.0364) attains the best calibration–sharpness trade-off among the seven models. N-BEATS is severely miscalibrated (PICP = 0.502, PINAW = 0.736).

RC-Mamba achieves the best Winkler score (0.0364), which jointly penalizes interval width and coverage violations and is the proper scoring rule for central-interval forecasts. Its PICP_80_ of 0.781 is the second closest to the nominal 80% target—LSTM's 0.783 is marginally closer (|0.783 − 0.80| = 0.017 vs. |0.781 − 0.80| = 0.019)—but LSTM is worse on every point and distributional metric, and its Winkler_80_ is more than three times larger. DeepAR instead over-covers at 0.889 using 39% wider intervals (PINAW = 0.033 vs. 0.024), which the Winkler score identifies as an over-dispersed trade. RC-Mamba therefore attains the best calibration–sharpness trade-off, leading DeepAR on Winkler by 12.9% (0.0364 vs. 0.0418).

We do not attribute this calibration to the Regime Uncertainty Gate. The gate is designed to widen intervals under high posterior entropy and narrow them when the regime is confident (Section 3.6), but the matched ablations of Section 4.7 do not identify that mechanism: a posterior-independent learned global scale and even a fixed unit scale both score better than the dynamic gate, in both ablation batches. What the regime-conditional comparison of Supplementary Table S11 (Supplementary Section S6.2) does establish is that the regime pathway as a whole widens predictive intervals by roughly 17% relative to a matched no-regime control, which moves coverage from 74.6% to 78.1% against a nominal 80%; the improvement is close to uniform across decoded market states rather than concentrated in stress, and it is approximately neutral under Winkler_80_.

### 4.5 Regime detection validation

The 3-state Student-*t* HMM is intended to capture distinct market regimes without explicit supervision. We validate the learned regimes along three dimensions: (i) economic interpretability, (ii) persistence, and (iii) alignment with known market episodes. Figure 6 presents the full regime analysis.

**Figure 6:**
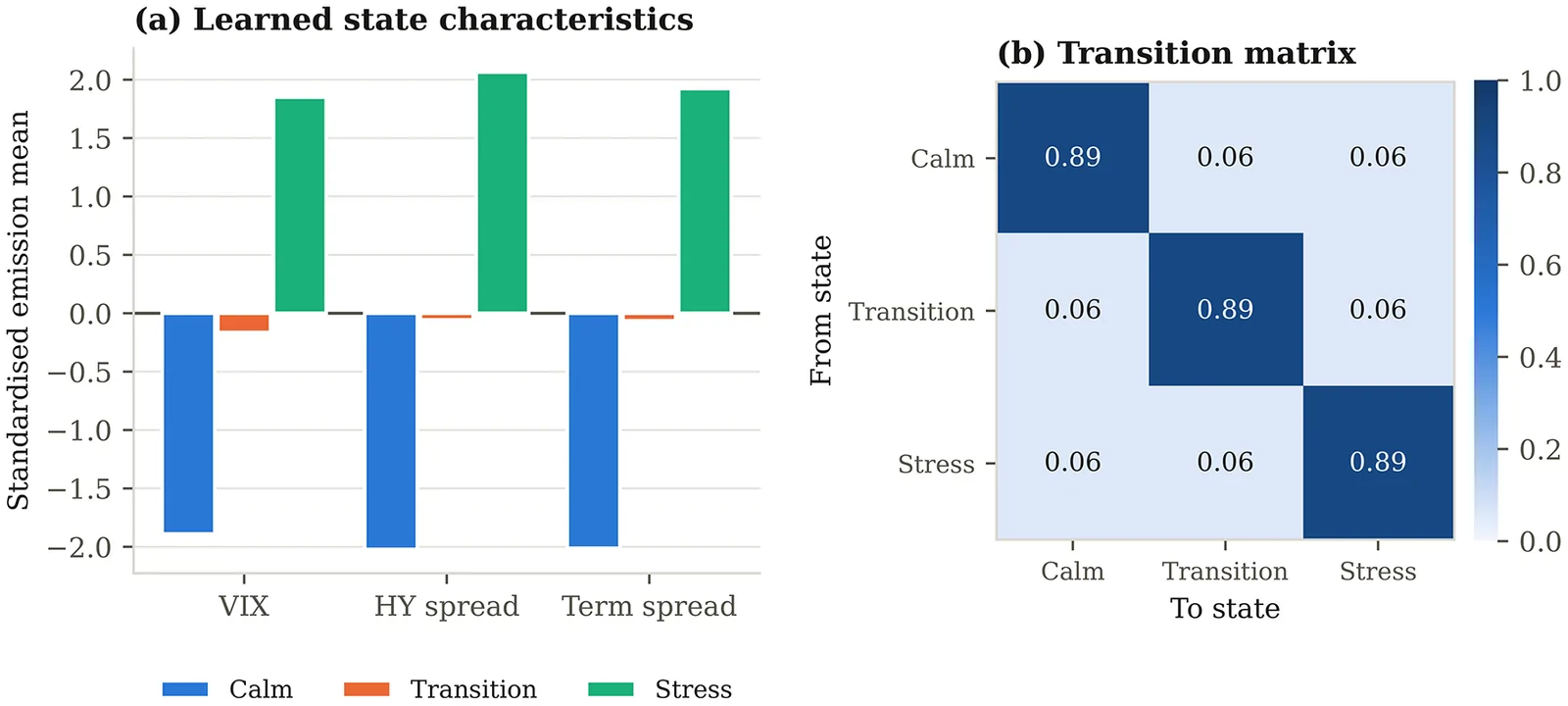
HMM regime analysis, pooled over the five fold-local fits. **(a)** Learned state characteristics in the standardized coordinates in which the HMM is fitted: Calm sits roughly two standard deviations below the training mean on implied volatility, credit spread and term spread simultaneously; Stress roughly two above on all three; Transition close to the mean on each. Raw levels are not shown because each fold standardizes on its own training window. **(b)** Transition matrix averaged across folds: the self-transition probability of 0.886 (range 0.874–0.905 by fold) reflects the sticky Dirichlet prior and implies a mean regime duration of 8–11 business days. Occupancy over the 1,110 forecast origins is strongly unbalanced—33.2% Calm, 2.5% Transition, 64.3% Stress—so the Transition state is a rare decode rather than a third of the sample. All 25 observed trading days of the April 2025 tariff episode are decoded as Stress.

The three states separate cleanly on all three observables. Emission means are reported in the standardized coordinates in which the HMM is fitted, averaged over the five fold-local fits; raw levels are not comparable across folds because each fold standardizes on its own training window.

- **State 0 (Calm):** VIX −1.89, high-yield spread −2.02, term spread −2.01—low volatility, tight credit and a flat-to-inverted curve relative to the fold's training mean.
- **State 1 (Transition):** VIX −0.16, HY spread −0.06, term spread −0.06—an intermediate state sitting close to the training mean on every observable.
- **State 2 (Stress):** VIX +1.85, HY spread +2.06, term spread +1.92—elevated implied volatility with simultaneously widening credit spreads.

Occupancy is strongly asymmetric across the test period. Over the 1, 110 forecast origins the decoded shares are 33.2% Calm, 2.5% Transition and 64.3% Stress; in the final fold alone they are 74.2%, 0.0% and 25.8%. Supplementary Table S6 reports the per-state occupancy, self-transition probability, expected duration and emission profile for that fold. The Transition state is therefore a genuinely rare decode rather than a third of the sample, and results conditioned on it—including the regime-conditional comparison in the Supplementary material and the Transition column of Table 6—rest on a small number of origins and should be read as indicative. Mean posterior confidence in the decoded state is 0.87 for Calm and 0.88 for Stress but only 0.55 for Transition, which is consistent with Transition acting as the boundary between the two dominant states rather than as a distinct third regime. Supplementary Figure S1 plots the causal posterior regime probabilities at all 1,110 evaluated forecast origins.

**Table 6:** Top-5 SHAP features per canonical regime, from *post-hoc* grouped permutation SHAP over 128 test samples drawn across all five final RC-Mamba checkpoints, all 18 ETFs and all three states (64 antithetic permutations per sample; maximum local-accuracy error 3.14 × 10^−9^).

Calm		Transition		Stress	
Feature	|ϕ| × 10^4^	Feature	|ϕ| × 10^4^	Feature	|ϕ| × 10^4^
EMVOVERALLEMV	1.444	WALCL	1.525	EMVOVERALLEMV	2.807
RBUSBIS	1.392	PAYEMS	1.138	EMVMACROBUS	1.621
GDPC1	1.167	EMVMACROBUS	0.997	FEDFUNDS	1.534
FEDFUNDS	0.991	RBUSBIS	0.984	RBUSBIS	1.407
WALCL	0.960	VIXCLS	0.791	PAYEMS	1.163

Values are mean |ϕ_*j*_| within each regime.

Self-transition probabilities are high and increase across the folds as more history becomes available, from 0.874 in fold 0 to 0.905 in fold 4 (mean 0.886), reflecting the sticky Dirichlet prior of Section 3.2. The implied mean regime duration 1/(1 − *a*_*kk*_) is 8 to 11 business days, so the prior delivers persistence on the scale of a fortnight rather than the multi-month regimes sometimes reported for lower-frequency macro models. That is long enough to prevent the high-frequency switching that would destabilize the SSM conditioning, which is the property the prior is there to provide.

### 4.6 Known-period validation

The April 2025 tariff-induced sell-off (2025-04-03 to 2025-05-08) falls inside the fold-4 test block and therefore has a decoded posterior at every forecast origin. The window spans 26 business days, 25 of which are trading days—18 April was a market holiday. The HMM assigns all 25 observed trading days in the pre-specified April 2025 stress episode to the Stress state. We report this as an assignment rather than a classification accuracy: the states are recovered without supervision and there is no labeled ground truth against which an accuracy could be scored.

No low-volatility counterpart is validated from 2026. Under the calendar-aligned protocol the test origins end on 2025-11-17, so 2026 dates lie outside the evaluated sample and cannot be checked without extending the walk-forward design. Restricted to what the protocol does decode, the HMM assigns the one well-documented stress episode inside the test period to the Stress state without supervision.

### 4.7 Ablation study

To quantify the contribution of each architectural component, we conduct ablation experiments using a single fold of the walk-forward protocol (Lim et al., 2021). Table 7 summarizes the key ablation variants; Figure 7 visualizes the change relative to the full model.

**Table 7:** Matched component ablations on fold 1 (test year 2022), the most volatile window in the design and therefore the most informative for a regime-conditioning mechanism.

Variant	CRPS	Δ %	PICP_80_ (%)	Winkler_80_	Calib. err.
RUG fixed to scale one	0.00759	−1.09	67.49	0.05197	0.0555
Learned global scale (posterior-independent)	0.00760	−0.92	66.28	0.05172	0.0649
Continuous macro conditioning	0.00763	−0.54	68.29	0.05198	0.0576
Full conditioning, gate on	0.00764	−0.35	62.06	0.05243	0.0792
Full conditioning, gate off (selected)	0.00767	—	60.94	0.05311	0.0829
Posterior appended as input	0.00773	+0.81	58.17	0.05451	0.0918
Condition Δ only	0.00776	+1.19	55.68	0.05492	0.1026
No regime conditioning	0.00783	+2.05	54.80	0.05624	0.1044

Every variant uses the same fold, seed, data, preprocessing and training schedule, so the rows form one internally comparable batch. ΔCRPS is relative to the selected gate-off configuration. Component effects of this magnitude are reported as design trade-offs rather than identified causal effects. Calib. err. is the normalized conformal calibration error of Section 3.10.14, averaged over the 50, 80, 90 % nominal levels.

**Figure 7:**
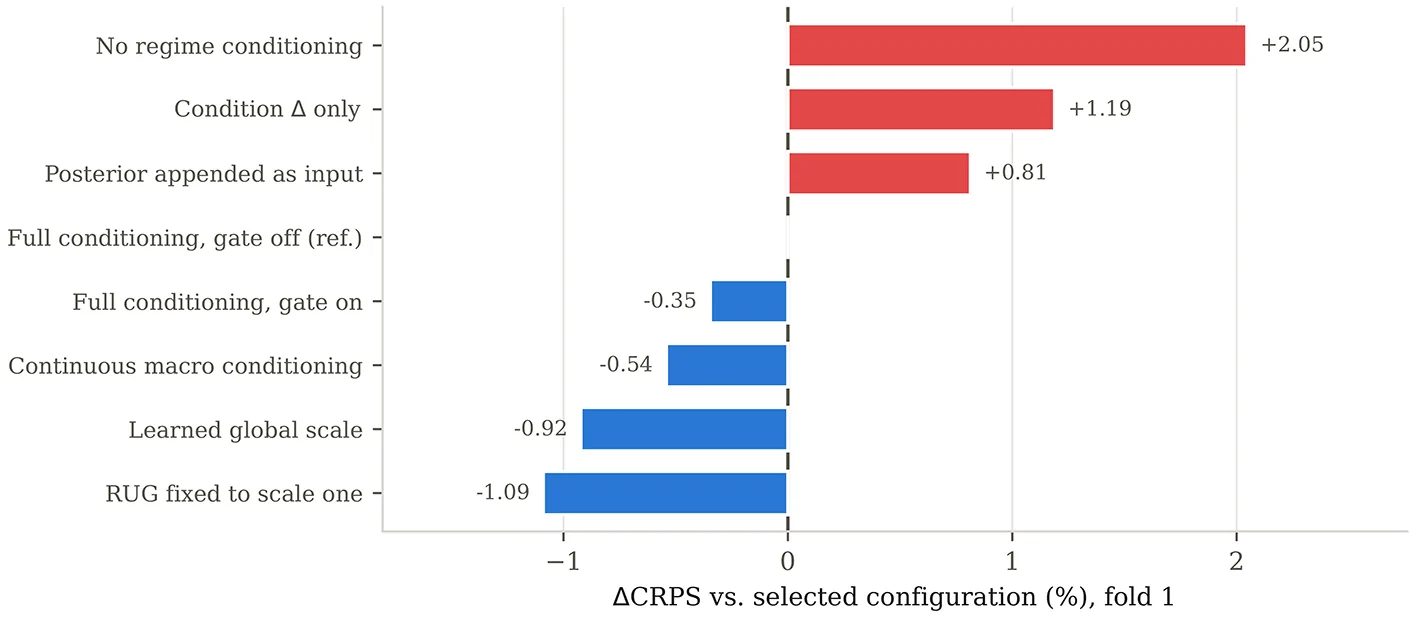
Matched component ablations on fold 1 (test year 2022), the most volatile window in the design. Bars show ΔCRPS relative to the selected gate-off configuration under an identical fold, seed, data pipeline and training schedule; red indicates degradation and blue improvement. Removing regime conditioning entirely is the most damaging single change (+2.05%). Component effects of this magnitude are reported as design trade-offs rather than identified causal effects.

Three findings stand out from the ablation analysis:

1. **Regime conditioning has the largest adverse removal effect in the volatile-fold batch, but the effect is not stable across folds**. Within the matched batch, removing the regime pathway entirely is the largest single degradation: CRPS rises by 2.05% relative to the selected configuration, and by 5.9%, the proper interval score. Measured the other way round—against the no-regime control as the baseline—full conditioning is 2.00% better, appending the posterior as an ordinary input 1.21% better, and conditioning Δ alone 0.84% better. Note that Δ-only is *worse* than append-only on CRPS, so the conditioning modes do not form a monotone accuracy ladder. The quantity that is monotone is interval coverage, which falls step by step as regime information is withdrawn: 62.1% (gate on), 60.9% (gate off), 58.2% (appended), 55.7% (Δ only), 54.8% (none), against a nominal 80%. All variants under-cover badly at fold 1; the conditioned ones under-cover less. This ordering is specific to fold 1. In the fold 0 batch the same no-regime control is 0.80% *better* than the selected configuration (Supplementary Table S22), so removing regime conditioning is the largest adverse change in the volatile window but not a stable effect across folds, and the five-fold origin-level comparison of Section 4.2 does not separate the no-regime control from RC-Mamba.
2. **Gate ablation supports Empirical Observation 1**. Disabling the SiLU gate reduces the block parameter count, but the matched ablations do not support the stronger claim that it also improves calibration: they marginally favor the gate-enabled variant (by 0.35% CRPS). The gate is therefore a parameter-efficiency choice whose accuracy direction is not established at this effect size. That is weaker than a redundancy result, but it remains consistent with regime conditioning through **B**_*t*_ and **C**_*t*_ subsuming the information-gating role of the SiLU activation (Section 3.3).
3. **Projection-level conditioning outperforms simple posterior appending in both reported ablation folds**. The contrast between full conditioning and appending the same posterior as an ordinary input isolates the paper's actual architectural claim, because both variants see identical regime information and differ only in where it enters. Conditioning the projections wins by 0.81% at fold 1 and by 1.88% at fold 0—one of only three contrasts in the batch that keeps its sign across both folds, and the one the narrowed novelty claim of Section 1 rests on. Four of the seven contrasts do change sign between folds, including the no-regime control (Supplementary Table S22 reports both batches side by side), which is why we present component effects of this magnitude as design trade-offs rather than identified causal effects. Note also that the Student-*t* emission choice is *not* ablated here: the matched batch varies the conditioning pathway and the uncertainty gate only, and the HMM specification is probed separately in Section 4.10.

### 4.8 Training budget and convergence

Every neural model is granted the same ceiling of 11,450 optimizer updates, with the realized budget determined by validation-based early stopping rather than by an asymmetric cap. Table 2 reports the realized counts. No baseline is stopped by the ceiling: DeepAR converges between 1,600 and 5,400 updates and VanillaTFT between 1,300 and 7,100, while RC-Mamba uses between 5,450 and 7,750 across its four stages. Any budget asymmetry between RC-Mamba and the baselines is therefore removed by construction rather than defended.

Figure 3 shows validation trajectories for RC-Mamba, VanillaTFT and DeepAR on all five folds, with the selected checkpoint marked. Each baseline reaches and then holds its best validation loss well inside the common ceiling, confirming that the comparison is not budget-limited.

To quantify the marginal return of the four-stage protocol, we additionally train RC-Mamba with Stage 2 only, a frozen HMM and a 500-step budget across all five folds. This reduced configuration is a deliberately curtailed variant of RC-Mamba used to price the schedule; it is not the budget given to any comparator, all of which share the 11,450-update ceiling of Section 3.10.13. Table 8 reports the outcome: the reduced protocol scores 0.00632 against 0.00532. Stated relative to the reduced protocol, the full schedule reduces CRPS by 15.8 %; stated relative to the full schedule, the reduced protocol has 18.7 % higher CRPS. The two figures are the same difference under the two denominators, not competing estimates.

**Table 8:** Training-budget sensitivity for RC-Mamba.

Configuration	CRPS	PICP_80_	Winkler_80_
Full four-stage protocol	0.00532	78.11	0.03640
Stage 2 only, 500 steps, frozen HMM	0.00632	83.14	0.04435

The 500-step variant runs Stage 2 only with the HMM frozen, isolating the marginal return of the full four-stage protocol. The reduced protocol has 18.7 % higher CRPS than the full schedule; equivalently, the full schedule reduces CRPS by 15.8 % relative to the reduced protocol.

### 4.9 Computational efficiency

Table 9 profiles RC-Mamba, VanillaTFT, DeepAR and S-Mamba on one NVIDIA RTX 3060 Laptop GPU using the same fold-0 loader, batch size and input shapes. RC-Mamba is more compact than VanillaTFT (697 K against 816 K parameters) but is not faster: it requires roughly 2.8× the epoch time and 3.2× the per-sample inference latency. The linear-time scan of the selective SSM does not translate into a wall-clock advantage at this sequence length, because the sequential scan is poorly suited to the parallelism of the device. We therefore claim parameter compactness relative to the transformer baseline, but not computational efficiency. Supplementary Table S9 gives the per-block asymptotic complexity, and Supplementary Section S4 the full profiling protocol.

**Table 9:** Efficiency comparison on a single NVIDIA RTX 3060 Laptop GPU using the same fold-0 loader, batch size and input shapes for every model.

Model	Params (K)	Epoch (s)	Infer. (ms/sample)	Peak CUDA (MiB)	FLOPs (G)
RC-Mamba	697.4	224.4	1.158	208.2	2.189
VanillaTFT	816.3	80.0	0.361	231.3	2.347
DeepAR	415.1	76.3	0.345	101.1	0.001
S-Mamba	153.7	168.1	0.450	146.7	0.133

Epoch time is the median profiled training-step time multiplied by the real loader length, excluding data loading. FLOPs cover only operators supported by the PyTorch profiler and are therefore a lower bound. RC-Mamba is more compact than VanillaTFT but is not faster at training or inference.

### 4.10 HMM specification sensitivity

Table 10 varies the state count and the observable subset. Validation likelihood favors three states, while BIC and the known-stress contrast can favor four; adding credit and term-spread observables improves separation of known stress episodes but weakens the VIX-threshold contrast. The three-state, three-observable specification is retained as a parsimonious and economically interpretable representation, not as evidence that markets possess exactly three regimes. The matched continuous-conditioning control in Table 7 addresses the complementary question of whether discretisation is necessary at all: on fold 1 it slightly outperforms the discrete conditioner, so discretisation is presented as an interpretability choice rather than an accuracy advantage.

**Table 10:** HMM specification sensitivity across state counts and observable subsets.

Configuration	*K*	Obs.	Val. NLL	BIC	Stress contrast
k2_vix	2	1	6.949	23,687	0.546
k3_vix	3	1	7.008	17,378	0.702
k4_vix	4	1	6.998	15,682	0.718
k2_vix_hy	2	2	14.979	69,381	0.501
k3_vix_hy	3	2	14.987	53,503	0.733
k4_vix_hy	4	2	14.991	51,436	0.800
k2_vix_hy_term	2	3	19.182	101,898	0.487
k3_vix_hy_term	3	3	18.988	79,030	0.762
k4_vix_hy_term	4	3	19.018	77,448	0.816

Validation NLL is per observation; the stress contrast is the difference in high-state probability between known stress episodes and the remaining sample. Three states with the VIX, high-yield spread and term spread are retained as a parsimonious financial representation, not as evidence that markets possess exactly three regimes.

### 4.11 Economic significance

The Diebold–Mariano and bootstrap evidence above establishes that RC-Mamba's distributional forecasts are statistically significantly more accurate than the baselines. This subsection asks the complementary question: does the distributional improvement translate into a measurable economic improvement at the portfolio level? We answer with a long-only, cross-sectional strategy evaluated over 1,255 out-of-sample trading days spanning 2021–2025, computed from the same calendar-aligned forecasts as the rest of the paper and executed with a one-day lag.

#### 4.11.1 Reconciling the sample sizes

Two denominators recur and should not be conflated. 599, 400 is the per-(asset, fold, day, horizon) count of the five-fold forecast grid, which is reduced to 1, 110 synchronized forecast origins for all statistical testing (Section 4.2) because the individual cells are strongly dependent. 1, 255 is the number of out-of-sample trading days in the economic backtest. The backtest is computed from the same calendar-aligned test forecasts as the statistical analysis rather than from a separate protocol on a longer price history, so the two analyses describe the same period and the same predictions.

#### 4.11.2 Signal and portfolio rule

Let ${\hat{q}}_{50,t}^{\left(i\right)}\left(h\right)$ denote the median log-return forecast for asset *i* at horizon *h* from the origin on day *t*. The signal is the cumulative median forecast over the full horizon (Equation 64),


$$\begin{array}{l} \text{signa}{\text{l}}_{t}^{\left(i\right)}=\overset{H}{\underset{h=1}{\sum }}{\hat{q}}_{50,t}^{\left(i\right)}\left(h\right),\;H=30. \end{array}$$ (64)

This is a sum of marginal medians rather than the median of the cumulative return distribution, which would require a forecast joint copula across horizons that the model does not produce; we use it as a ranking statistic only. Each trading day the available tickers are ranked by $\text{signa}{\text{l}}_{t}^{\left(i\right)}$ and the strategy goes long the top ⌈*N* × 50%⌉ in equal weight, executed with a one-day lag. The benchmark is a daily equal-weight buy-and-hold of all 18 ETFs over the same dates. Results are reported gross of cost and at 1, 5 and 10 basis points of turnover (Table 11).

**Table 11:** Long-only top-50 % strategy under transaction-cost sensitivity, computed from the revised calendar-aligned forecasts with a 1-day execution lag over 1,255 trading days.

Cost (bp)	Ann. ret. (%)	Sharpe	Max DD (%)	Alpha (%)	95 % CI	*p*
0	8.89	0.666	–24.3	1.18	[−2.29, +4.64]	0.506
1	8.83	0.662	–24.3	1.14	[−2.33, +4.60]	0.521
5	8.60	0.647	–24.6	0.98	[−2.49, +4.44]	0.581
10	8.32	0.629	–25.0	0.78	[−2.69, +4.24]	0.660

The equal-weight benchmark over the same universe returns 6.56 % annualized with a Sharpe ratio of 0.612; annualized turnover is 5.18. Alpha is the intercept of the strategy return regressed on the benchmark return. The alpha is not statistically distinguishable from zero at any cost level, so the economic evidence is suggestive rather than conclusive.

#### 4.11.3 Headline results

Table 11 reports the portfolio-level metrics. Relative to the equal-weight buy-and-hold, the RC-Mamba strategy returns 8.89 % annualized against 6.56 %, with a Sharpe ratio of 0.666 against 0.612 and a maximum drawdown of −24.3 %. These differences are, however, *not statistically significant*: the annualized alpha is 1.18 % with a 95 % confidence interval of [−2.29%, +4.64%] and *p* = 0.506, and it remains insignificant under transaction costs of 1–10 basis points (*p* = 0.660 at 10 bp). Annualized turnover is 5.18. We therefore regard the economic evidence as suggestive rather than conclusive, and we do not claim that RC-Mamba generates significant risk-adjusted alpha.

#### 4.11.4 Statistical test on the alpha

The alpha is the intercept of the daily strategy return regressed on the daily benchmark return, with a Newey–West HAC covariance at 30 lags and lag products constrained within folds. The estimated beta is 1.19, so the strategy is more exposed to the common factor than the benchmark it is measured against, and roughly half of the 2.3-percentage-point return gap is compensation for that exposure rather than selection skill. The residual annualized alpha is 1.18% with *z* = 0.664 and *p* = 0.506: the null of zero alpha is not rejected, and would not be rejected at any of the four cost levels. In the language of the efficient markets hypothesis, RC-Mamba does not produce a statistically significant risk-adjusted alpha on this sample.

#### 4.11.5 Caveats

Realistic trading costs (1–5 bps for liquid US ETFs, 10–30 bps for emerging-market and high-yield bond ETFs) sit at or above the range tabulated here, and annualized turnover of 5.18 makes the strategy cost-sensitive. The strategy's maximum drawdown (−24.3%) is slightly *worse* than the benchmark's (−23.5%), so we do not claim improved drawdown control. The equal-weight top-50 % rule is not cross-validated, and the ranking statistic uses only the median forecast, discarding the interval information that is the model's actual strength. The backtest is a direction-of-effect check on a 1,255-day sample, not a tradable strategy, and it does not by itself support an economic claim for regime conditioning.

### 4.12 Interpretability analysis

We apply the regime-conditioned interpretability framework of Section 3.8 to examine model behavior through three complementary lenses: (i) intrinsic VSN selection weights (Equation 56), (ii) *post-hoc* SHAP attribution conditioned on HMM regimes (Equation 53), and (iii) per-asset attribution profiles (Equation 55).

All attributions come from the grouped permutation SHAP analysis of Section 3.8.2, computed over 128 evaluation samples drawn across all five final RC-Mamba checkpoints, all 18 ETFs and all three decoded regimes, with 64 antithetic permutations per sample. Because a feature carries all 24 of its lags as a single player, the analysis yields 128 × 30 = 3, 840 attributions in total. The baseline is zero in the training-fold robust-MAD coordinates, that is the training-period median. Local accuracy holds to 3.14 × 10^−9^ in the worst sample. All 30 features receive non-trivial attribution, spanning 1.3 × 10^−5^ to 1.5 × 10^−4^ in mean |ϕ_*j*_|; none is discarded. The regime split is unbalanced by construction—51 Calm, 40 Transition and 37 Stress samples—because the draw balances coverage of the three decoded states rather than matching their occupancy.

#### 4.12.1 Dual-lens variable importance

Supplementary Table S21 presents the top-10 features ranked by two independent criteria: ${\bar{v}}_{j}$ (Equation 56), which captures how much the model *selects* each input at the variable-selection gate, and ${\bar{\phi }}_{j}$ (Equation 51), which measures each feature's marginal *contribution to the prediction*.

Neither lens is concentrated. The VSN spreads its selection weight broadly: the largest single share is 9.3% (breakeven inflation rate), and no feature exceeds a tenth of the gate. SHAP attribution is similarly flat, with the leading feature (EMVOVERALLEMV, the overall equity-market-volatility news index) at 1.52 × 10^−4^, only 1.20× the second-ranked (RBUSBIS at 1.27 × 10^−4^). Neither the input gate nor the output attribution is dominated by one covariate, so we do not claim a single driving feature.

The two rankings nonetheless disagree substantially, which is the result that matters architecturally. Six of the ten features are shared, but their order is very different, and the largest disagreements run in both directions: FEDFUNDS is 6th on SHAP but only 18th on VSN weight, while breakeven inflation is 1st on VSN weight and outside the SHAP top 10. The VSN operates *upstream* of the RC-Mamba block, controlling what enters the temporal backbone, whereas SHAP measures *end-to-end* sensitivity of the output. A feature can receive low selection weight at the gate and still exert large marginal influence through nonlinear interactions inside the recurrence, and vice versa. A linear pass-through would make the two lenses proportional; they are not, which is direct evidence that the backbone performs substantial nonlinear transformation of its inputs.

Figure 8 visualizes this dual-lens comparison for the ten features that rank highest on SHAP attribution; the Stress/Calm amplification ratios are reported numerically below.

**Figure 8:**
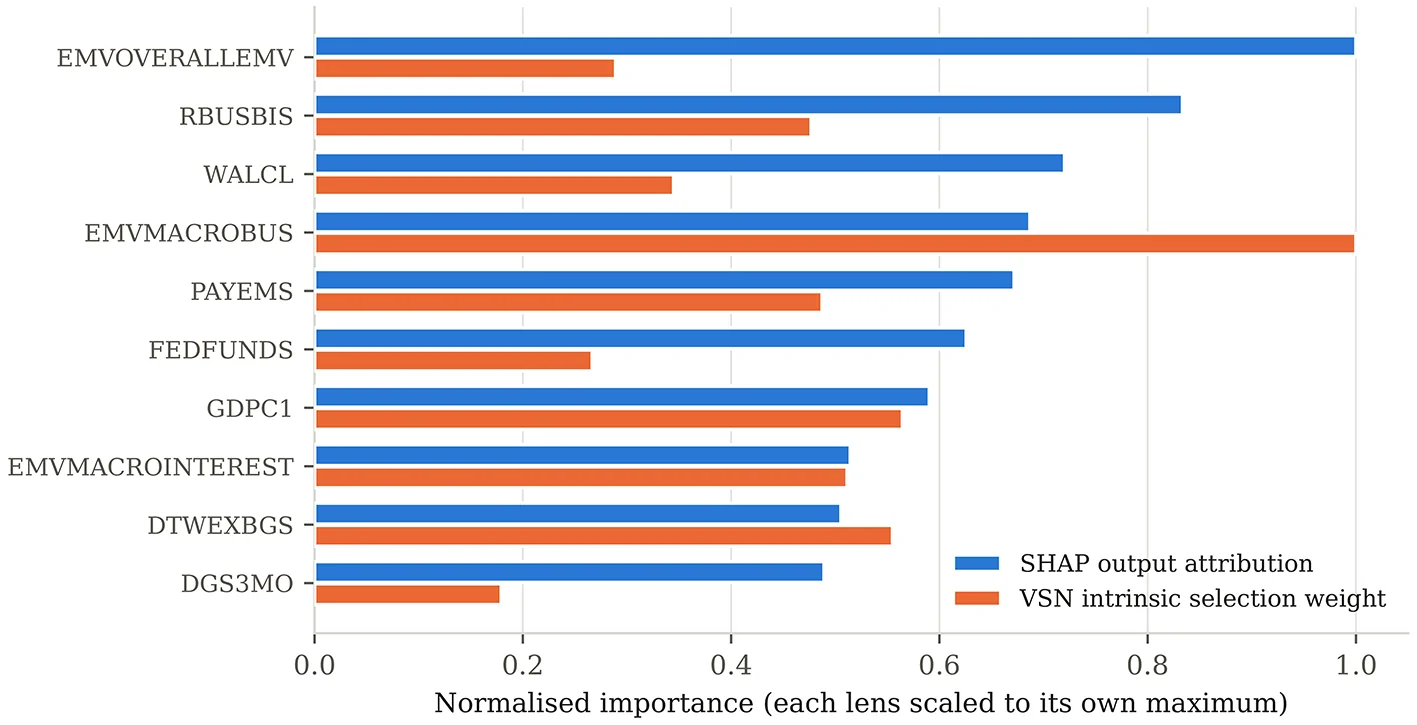
Dual-lens variable importance from post-hoc grouped permutation SHAP over 128 test samples spanning all five final RC-Mamba checkpoints, all 18 ETFs and all three canonical regimes. Bars show the ten features with the largest SHAP output attribution; for each feature the blue bar is the SHAP output attribution and the orange bar the intrinsic VSN selection weight, with each lens normalized to its own maximum. Neither lens is concentrated: the leading SHAP feature exceeds the second by only 1.20×, and across the full 30-feature set the largest single VSN selection share is 9.3 %. The two orderings disagree, so the features the network selects intrinsically are not the features with the largest effect on the predictive distribution, and intrinsic gate weights alone are an unreliable interpretability signal.

The orange bars in Figure 8 give the VSN selection weights. Selection is diffuse across the full 30-feature set: the top ten by selection weight carry 56 % of the gate between them and the remaining twenty share the rest. The leading entries are slow-moving macro and market-structure factors — breakeven inflation (9.3 %), macro business-uncertainty EMV (8.9 %) and the high–low range (6.4 %) — consistent with a gate that prefers signals stable across regimes. Only the second of these appears among the ten features plotted, which are ranked by SHAP attribution.

The blue bars in Figure 8 give the SHAP attributions, which order the same feature set differently. Economic-uncertainty indices lead (EMVOVERALLEMV 1.52 × 10^−4^), followed by BIS credit-to-GDP (1.27 × 10^−4^) and the Fed balance sheet (1.10 × 10^−4^). FEDFUNDS ranks 6th at 9.53 × 10^−5^ despite ranking 18th on VSN weight, which is the clearest single instance of the recurrence amplifying an input the gate does not prioritize.

We also quantify the regime differential, as the ratio of mean |ϕ_*j*_| under Stress to that under Calm; these ratios are reported below rather than plotted. The amplification is moderate — the largest over the 30-feature set is 2.9×, well short of an order of magnitude. Five features are amplified: VIXCLS (2.87×), macro business-uncertainty EMV (2.43×), realized volatility Std_dev (2.00×), overall EMV (1.94×) and macro interest-rate uncertainty (1.67×). Five are attenuated: Volume (0.61×), the daily high (0.68×), the 10-day moving average (0.71×), long-term government yields (0.78×) and DGS10 (0.80×). The pattern is economically coherent: under stress the model shifts weight toward volatility and uncertainty measures and away from level and trend features. The model therefore does not suppress the VIX and its relatives when they matter most; it leans on them.

#### 4.12.2 Regime-conditioned SHAP attribution

While the Stress/Calm ratios reported above summarize the two extreme regimes, the full three-regime decomposition reveals that the Transition regime (State 1) is *not* an intermediate blend of Calm and Stress but exhibits its own distinctive attribution signature. Table 6 presents the per-regime top-5 SHAP features and Figure 9 shows the full three-regime comparison for the top-10 globally important features. Supplementary Table S7 lists the same per-regime top-5 ranking in tabular form.

**Figure 9:**
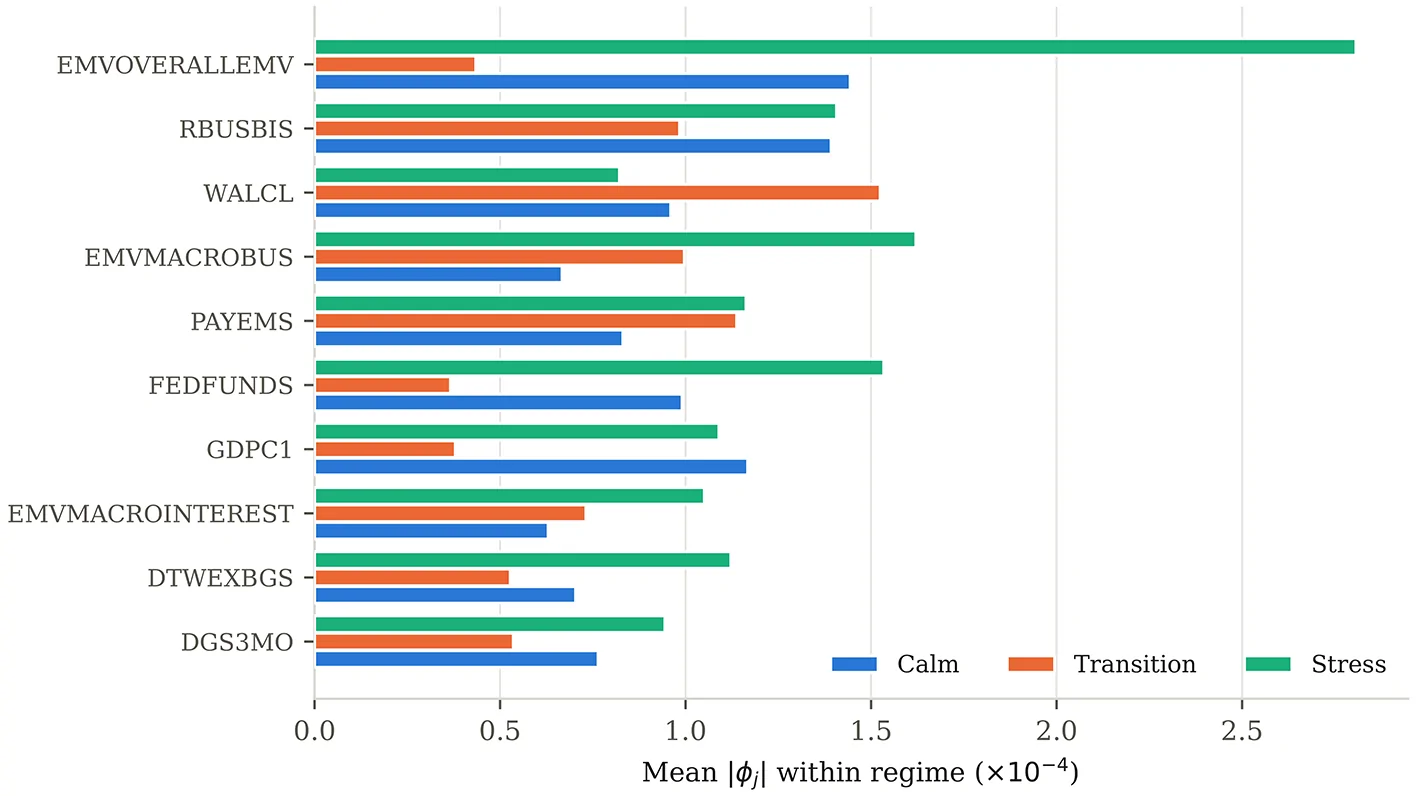
Three-regime SHAP decomposition for the top-10 globally important features. Each group shows three bars: Calm (blue), Transition (orange) and Stress (green). No regime is dominated by a single covariate—the leading feature accounts for 8.5%, 10.0%, and 13.0% of the within-regime total in Calm, Transition and Stress respectively. The Fed balance sheet (WALCL) peaks under Transition (1.52 × 10^−4^), where the policy rate (FEDFUNDS) is at its lowest (3.66 × 10^−5^); FEDFUNDS peaks under Stress (1.53 × 10^−4^). That inversion is what distinguishes regime conditioning from a uniform rescaling of one importance profile. The Transition column rests on 40 evaluation samples drawn from a state that accounts for 2.5% of forecast origins, and is reported as indicative.

Transition is not an interpolation between Calm and Stress. Its leading feature is the Fed balance sheet, WALCL, at 1.52 × 10^−4^—higher than WALCL attains in either Calm (9.60 × 10^−5^) or Stress (8.22 × 10^−5^) — followed by payrolls and macro business uncertainty. FEDFUNDS, by contrast, is at its *lowest* in Transition (3.66 × 10^−5^, against 9.91 × 10^−5^ in Calm and 1.53 × 10^−4^ in Stress). The state therefore reads as balance-sheet and labor-market driven rather than policy-rate driven.

No regime is dominated by a single feature. The leading feature accounts for 8.5% of total attribution in Calm, 10.0% in Transition and 13.0% in Stress, so the shift across regimes is a reordering of a broad factor set rather than a collapse onto one covariate. Stress shows the most concentration and the most distinctive profile: overall economic uncertainty (2.81 × 10^−4^), macro business uncertainty (1.62 × 10^−4^) and FEDFUNDS (1.53 × 10^−4^) lead, each roughly double its Calm value.

Two cautions apply. Transition rests on 40 of the 128 sampled evaluation points but only 2.5% of decoded forecast origins (Section 4.5), so its profile is estimated on a state the model rarely commits to; and mean posterior confidence in Transition is 0.55, against 0.87–0.88 for the other two states. We therefore report the Transition profile as suggestive.

The implication for the architecture is that conditioning (Δ, **B**, **C**) on γ_*t*_ does not merely rescale a single importance profile: the ordering of the factor set changes across states, and one feature (WALCL) peaks in the state where another (FEDFUNDS) troughs, which a uniform rescaling cannot produce. The effect is real but moderate—the largest regime ratio over the feature set is under 3× —and we present it as evidence that the conditioning pathway is doing something regime-specific, not as evidence that it improves forecast accuracy, which Section 4.7 does not support.

#### 4.12.3 Asset-level attribution patterns

The per-ticker analysis (Equation 55) groups samples by asset identity; Supplementary Figure S8 gives the full heatmap over all 18 ETFs and the eight most discriminative features, and Supplementary Table S8 lists the top-three attributed features for each ETF. The dominant axis of variation is scale, not composition: per-ticker attributions span roughly an order of magnitude, from the bond and credit ETFs (leading values 3.8–7.3 × 10^−5^) to the equity and emerging-market ETFs (2.6–4.7 × 10^−4^), an ordering that follows each asset's return volatility, as expected for attributions measured on the predicted-return scale. Composition does not separate cleanly by asset class once that scaling is discounted, and it is not uniform even within a block: among the four US equity ETFs, IVOO and VIOO are led by economic uncertainty and FEDFUNDS, SPY by the Fed balance sheet and VOO by the intraday high–low range, although VOO and IVOO track closely related indices. With 5–10 evaluation points per ticker, we report these profiles as descriptive and do not claim that unsupervised asset-class structure has been recovered.

#### 4.12.4 Interpretability synthesis

One result survives all three lenses, and it is a narrow one. Selection diverges from attribution: the VSN's ordering and the SHAP ordering share six of ten features but rank them very differently, with FEDFUNDS 6th on output attribution and 18th at the input gate. A linear pass-through would make the two proportional, so the divergence is direct evidence that the regime-conditioned recurrence performs substantial nonlinear transformation rather than forwarding its inputs. The regime decomposition adds that the transformation is state-dependent: the factor ordering changes across Calm, Transition and Stress, and WALCL peaks in the state where FEDFUNDS troughs, which uniform rescaling cannot produce.

Two stronger readings are not supported by the analysis. Neither lens is concentrated on a single covariate, and the per-ticker profiles cluster by volatility scale rather than by asset class on a sample of 5–10 evaluation points per ticker, so we do not claim that asset-class structure is recovered without supervision. The interpretability evidence therefore supports the claim that regime conditioning changes *how* the model processes information, and is silent on whether that change improves forecasts—which is the question Section 4.7 addresses directly, and answers less favorably.

## 5 Discussion

RC-Mamba achieves statistically significant improvements in probabilistic forecast accuracy and calibration over all six retained comparators (Sections 4).

### 5.1 Answers to the research questions

**RQ1** is answered affirmatively at the level of the complete system: RC-Mamba attains the lowest CRPS and the best Winkler_80_ of the seven models, and every pairwise comparison survives Holm correction over the retained family (Table 4). The attribution is narrower than the ranking, however. The one component contrast that keeps its sign across both matched batches is projection-level conditioning against appending the same posterior as an ordinary input; the no-regime control changes sign between folds and is not separated from RC-Mamba over the five-fold origin panel. The evidence therefore supports the conditioning *interface* rather than a quantified causal contribution of the regime pathway as a whole.

**RQ2** is answered affirmatively for persistence and separation: the fold-local fits recover three states that separate on all three observables with self-transition probabilities of 0.874 to 0.905, and all 25 observed trading days of the April 2025 stress episode decode to the Stress state without supervision (Section 4.6).

**RQ3** is answered in the negative. Posterior-entropy scale modulation does not improve calibration beyond posterior-independent scale control: in both matched batches a learned global scale that ignores the posterior entirely is at least as good as the gate (−0.66% at fold 0 and −0.92% at fold 1 in CRPS), and fixing the gate output to unity is better in the volatile fold and worse in the calm one. The mechanism is retained in the architecture description because it is part of the evaluated system, but the matched experiment does not identify a gain from it. Below we interpret the mechanisms behind these gains, contextualize the architecture within financial forecasting research, and identify limitations.

### 5.2 Why regime conditioning works

The central design thesis of RC-Mamba is that injecting macro-structural information *directly* into the SSM recurrence—rather than treating it as an auxiliary input to an attention layer—lets the model adapt its temporal processing to the current market regime. The ablation results (Section 4.7) support a narrow form of this thesis. Full conditioning (where γ_*t*_ modulates Δ, **B**, and **C**; Equation 18) beats appending the identical posterior as an ordinary input by 0.81% at fold 1 and 1.88% at fold 0—the same regime information, entering at a different point—and beats conditioning Δ alone by 1.19%. Against the regime-agnostic Vanilla TFT, the complete system leads by 4.7% on five-fold mean CRPS. What the ablations do *not* show is that the regime pathway is what produces the five-fold advantage: a matched no-regime backbone is statistically indistinguishable from RC-Mamba over the same origins (Supplementary material), so the conditioning interface is supported as an architectural choice without being identified as the source of the benchmark result.

Mechanistically, the three conditioned parameters serve distinct roles. The discretisation step Δ_*t*_ controls the *temporal resolution* of the SSM: under Stress, which the HMM decodes with mean posterior confidence 0.88, the model can learn a shorter effective memory and react faster to changing conditions. The input mixing matrix **B**_*t*_ determines *what enters the hidden state*, and the readout matrix **C**_*t*_ controls *what is emitted*. The interpretability analysis (Section 4.12.2) is consistent with both being regime-dependent: the factor ordering differs across states, and the Fed balance sheet peaks under Transition while the policy rate peaks under Stress. We stress that this evidence is associational. Attributions are measured at the model output and cannot be assigned to **B**_*t*_ rather than **C**_*t*_, so the mechanism narrative below is an interpretation of a regime-dependent pattern, not a measurement of the individual projections.

This three-way modulation exceeds what attention-based architectures can achieve. In the vanilla TFT, self-attention applies a single set of query-key-value projections regardless of regime; any adaptation must emerge implicitly. In RC-Mamba, γ_*t*_ is a *first-class parameter* of the recurrence, so every forward step through the sequence explicitly adapts to the current market state.

The sticky Dirichlet prior (Section 3.2) further supports this mechanism by encouraging regime persistence (self-transition probabilities of 0.874 to 0.905 across the five fold-local fits), which prevents the HMM from producing noisy, high-frequency regime switches that would destabilize the SSM conditioning. The implied mean duration of 8 to 11 business days is shorter than the multi-month regimes reported for lower-frequency macro models (Bulla et al., 2011), but it is long relative to the *T* = 24-day lookback, which is the scale that matters for the conditioning pathway.

### 5.3 The SiLU gate

In the standard Mamba architecture (Gu and Dao, 2023), the SiLU gate acts as a learnable information bottleneck: **y**_gated_ = **y**_inner_ ⊙ SiLU(**z**), modulating which dimensions of the SSM output reach downstream layers. The argument for redundancy was that the regime-conditioned input matrix **B**_*t*_ already controls what enters the hidden state and the conditioned readout **C**_*t*_ controls what is emitted, so a regime-agnostic multiplicative gate adds a second, less expressive routing pathway.

That argument is not falsified by the data, but neither is it confirmed: under a matched fold, seed, data pipeline and training schedule, the gate-enabled variant is marginally *better* on every reported metric—CRPS 0.00764 against 0.00767 (−0.35%), PICP_80_ 62.1% against 60.9%, Winkler_80_ 0.05243 against 0.05311, and mean calibration error 0.0792 against 0.0829 (Table 7). The same sign appears in the fold-0 batch (−0.55%). The differences are smaller than the fold-to-fold variation of every other component contrast, so we read them as evidence that the gate is not a decisive design choice at this scale rather than as a reversal in favor of the gate.

We nevertheless report the gate-off configuration throughout, because it is the configuration that was pre-selected before the ablations were run. The gate is therefore presented as a parameter-efficiency choice whose accuracy direction is not established (Empirical Observation 1), and we make no practical recommendation that internal gating “may be safely removed” from conditioned-SSM designs.

### 5.4 N-BEATS failure under walk-forward evaluation

N-BEATS (Oreshkin et al., 2020) under-performed sharply on folds 1–2 (CRPS 0.2223 and 0.2633 against 0.00505 in its best fold; Supplementary Table S20) under an identical data pipeline and hyperparameter protocol (Section 3.10.6). The cause is architectural: as a pure basis-expansion model with no exogenous-input capacity, N-BEATS relies solely on lagged targets and cannot observe the macro features (FEDFUNDS, WALCL, VIX, …) that signal the regime transitions concentrated in those test windows, so it extrapolates from in-sample basis coefficients and diverges. On a diversified, regime-shifting multi-asset universe, models without exogenous covariates are fragile, and macroeconomic inputs are a precondition for walk-forward reliability rather than a marginal refinement.

### 5.5 Calibration and the regime uncertainty gate

RC-Mamba attains the best interval forecast under the proper scoring rule: the lowest Winkler_80_ (Table 5) and the best calibration–sharpness trade-off. DeepAR's PICP_80_ = 0.889 over-covers relative to RC-Mamba's 0.781, but only through wider intervals (PINAW_80_ 0.033 vs. 0.024); because the Winkler score jointly penalizes coverage shortfall and width, it ranks RC-Mamba (*W*_80_ = 0.0364) ahead of DeepAR (0.0418) by 12.9% (Gneiting and Raftery, 2007). The regime pathway as a whole contributes to this by widening intervals relative to a matched no-regime backbone, which under-covers by 5.4 points; the matched ablations do not, however, identify the Regime Uncertainty Gate as the source, since a posterior-independent scale performs at least as well (Section 4.7). The LSTM illustrates the opposite failure mode: its PICP_80_ of 0.783 is nominally the closest to target of any model, yet it attains that coverage with intervals four times wider (PINAW_80_ 0.104 against 0.024) and a Winkler_80_ more than three times worse. Coverage alone is not a calibration criterion, which is precisely why the proper interval score is the one we rank on.

### 5.6 Interpretability as architectural validation

The interpretability analysis (Section 4.12) validates one architectural property and fails to validate two others. What it does establish is that VSN selection weights and SHAP output attributions rank the same feature set very differently—FEDFUNDS is 6th on output attribution and 18th at the input gate, breakeven inflation the reverse—which is direct evidence that the regime-conditioned recurrence performs substantial nonlinear feature transformation, since a linear pass-through would make the two lenses proportional. The three-regime decomposition adds that the transformation is state-dependent rather than a single profile rescaled: the Fed balance sheet peaks under Transition, where the policy rate troughs, and no rescaling can produce that inversion.

Two stronger readings the analysis might invite are not supported by it. Neither lens is dominated by a single covariate—the largest VSN share is 9.3% and the leading SHAP feature exceeds the second by only 1.20× —so a “one feature drives the output” account does not hold. And the per-ticker profiles cluster by return volatility rather than by asset class, on 5–10 evaluation samples per ticker, so they are not evidence that the hierarchical FiLM embedding recovers asset-class structure without supervision. The FiLM layer remains a component of the architecture; the interpretability analysis simply does not argue for it.

### 5.7 Connection to the efficient markets hypothesis

RC-Mamba's claims are consistent with the efficient markets hypothesis (EMH). The model does not predict deterministic price moves or risk-free alpha—daily directional accuracy (50.7 %) is barely above a coin flip and economically marginal before costs. Its gains are *distributional*: better-calibrated, sharper intervals with regime-adaptive uncertainty. These exploit well-documented, EMH-compatible structure—volatility clustering, regime-dependent risk premia, and distributional non-stationarity (time-varying tail thickness and skew)—none of which violates semi-strong efficiency. The practical value is therefore in risk management (portfolio construction, risk budgeting, regulatory capital), where distributional accuracy matters more than directional prediction.

### 5.8 When RC-Mamba underperforms

For balance, it is worth cataloging the cases in which RC-Mamba does not dominate the baselines. Four patterns emerge from the per-fold, per-asset, and per-horizon evidence.

#### 5.8.1 Assets where the advantage does not hold

Pooling per-ticker CRPS across folds, RC-Mamba is ahead of the Vanilla TFT on 16 of 18 ETFs and behind on two—IVOO and VIOO, the mid- and small-cap US equity trackers—and the same two assets are the exceptions against DeepAR. Both are US equity funds rather than international trackers, so the loss cannot be attributed to the US-centric feature set of Section 5.9. We have no mechanism to offer for the pattern, and record it as an open point. The one fold in which RC-Mamba is not first overall is fold 1, where the Vanilla TFT leads by 0.63%.

#### 5.8.2 Modest directional accuracy

Directional hit rate ranges from 47.8% to 52.7% across the five folds and averages 50.7%, and HMM-DeepAR is nominally ahead of RC-Mamba on this metric (51.3%). RC-Mamba is therefore unsuitable as a stand-alone sign-of-return predictor; its value lies in the distributional forecast rather than in directional accuracy, and the backtest of Section 4.11 does not produce significant alpha either.

#### 5.8.3 The reported configuration is not the best in the matched batch

Three of the eight variants in Table 7 score below the selected gate-off configuration on fold 1: a fixed unit scale (−1.09%), a posterior-independent learned global scale (−0.92%) and direct continuous macro conditioning (−0.54%), with the gate-enabled variant a fourth (−0.35%). We report the pre-selected configuration rather than the ablation winner, for the reason given in Section 5.3, but the honest reading is that several simpler alternatives are at least competitive with the architecture as specified, and two of them dispense with the Regime Uncertainty Gate altogether.

#### 5.8.4 Coverage is unstable across folds

PICP_80_ ranges from 0.609 in fold 1 to 0.924 in fold 3, so the five-fold mean of 0.781 conceals substantial fold-to-fold swing in both directions: the model under-covers sharply in the 2022 stress window and over-covers in 2024. No model in the panel is stable on this metric—the tightest is DeepAR at 0.838–0.923, and it over-covers in every fold—but the swing means that a coverage guarantee cannot be read off the aggregate figure. Conformalisation per fold would be the natural remedy and is not attempted here.

### 5.9 Limitations

We identify five principal limitations of the current work:

1. **Computational overhead**. RC-Mamba is slower than the Vanilla TFT at both training and inference. Under identical loaders, batch size and input shapes (Section 4.9), it requires 2.8× the epoch time (224.4 s against 80.0 s) and 3.2× the per-sample inference latency (1.158 ms against 0.361 ms). The training-side cost is compounded by the four-stage protocol (Section 3.9), whose Stage 1 and Stage 3 add ~1,050 gradient steps beyond the main loop. The inference penalty is the more consequential of the two, because it cannot be amortized by retraining less often; it arises because the sequential selective scan maps poorly onto GPU parallelism at this sequence length, so the architecture's linear-time asymptotics do not yield a wall-clock advantage. Replacing the sequential scan with the parallel scan of Mamba-2 (Dao and Gu, 2024) is the obvious remedy and is untested here. RC-Mamba is more compact than the TFT (697 K against 816 K parameters) and uses less peak GPU memory (208.2 against 231.3 MiB), so we claim compactness but not efficiency.
2. **Discrete regime states**. The 3-state HMM imposes a discrete regime structure on market dynamics. While the Student-*t* emissions and sticky Dirichlet prior produce persistent state assignments, the decode is strongly unbalanced—Stress and Calm account for 97.5% of forecast origins and Transition is decoded with mean posterior confidence of only 0.55 (Section 4.5), so in practice the fitted model behaves closer to a two-state system with a low-confidence boundary. A matched control that injects the three continuous macro observables directly, without discretisation, slightly outperforms the discrete conditioner on fold 1 (Table 7). The underlying assumption of three discrete states may miss continuous regime gradients or transient micro-regimes. A continuous latent dynamics model—for example, a neural stochastic differential equation or a normalizing flow over latent regime space—could capture finer regime structure at the cost of additional model complexity and training instability.
3. **US-centric macro feature selection**. Of the 30 historical features used by the model, the macroeconomic covariates are sourced predominantly from US data series (FEDFUNDS, WALCL, DGS2/3MO/10, DFII10, VIXCLS, CPI, GDP, payrolls). The only feature with explicit multi-market coverage is DTWEXBGS (trade-weighted USD index). While US monetary policy exerts global influence—consistent with FEDFUNDS dominating SHAP attribution even for international ETFs (Section 4.12.3)—the model lacks local monetary policy and economic indicators (e.g., ECB rates, Bank of England base rate, SHIBOR, local CPI) that could improve forecasts for non-US assets. The weak SHAP signal observed for FXI (China) and the reliance on DTWEXBGS for VGK (Europe) suggest that local macro features would provide complementary information.
4. **Daily frequency only**. All experiments use daily closing prices. Intraday dynamics (microstructure effects, opening/closing auctions, overnight gaps) are not captured. Extension to higher frequencies would require rethinking the HMM observable design (VIX and credit spreads are inherently daily) and addressing the computational cost of longer sequences in the Mamba recurrence.
5. **No explicit cross-asset dependency modeling**. RC-Mamba processes each asset independently, sharing only the global macro features and the sector/ticker FiLM embedding. It does not model cross-asset correlations, contagion effects, or lead-lag relationships between assets. The per-ticker SHAP analysis (Section 4.12.3) shows per-asset factor profiles that differ, but they track return volatility more cleanly than asset class and rest on a small per-ticker sample, so they are not evidence of learned cross-asset structure. Graph neural network overlays or cross-attention between asset streams could address this gap.

These limitations define natural extensions, discussed in Section 5.10.

### 5.10 Conclusions

RC-Mamba demonstrates that injecting HMM regime posteriors directly into a Mamba SSM's recurrence parameters—Δ, **B**, and **C**—yields measurable gains in probabilistic forecast accuracy, calibration, and interpretability over regime-agnostic alternatives. On 18 ETFs across five asset classes under five calendar-aligned walk-forward folds with a 30-business-day embargo, the model achieves a mean CRPS of 0.00532 with statistically significant improvements over all six comparators (*p*_Holm_ = 0.006, origin-level moving-block bootstrap).

#### 5.10.1 What the experiments confirm

Matched single-fold ablations support one architectural choice specifically: conditioning the selective projections on the posterior beats appending that posterior as an ordinary input, in both ablation batches. Removing regime conditioning altogether is the most damaging single change in the fold-1 batch (+2.05% CRPS). Removing the SiLU gate does not improve calibration—the matched contrast marginally favors the gate-enabled variant, and the gate-off configuration is retained only because it was pre-selected (Section 4.7). The Student-*t* HMM with sticky Dirichlet prior recovers three economically interpretable regimes with self-transition probabilities of 0.874 to 0.905 across the five fold-local fits, and decodes all 25 observed April 2025 trading days to the Stress state. The model attains the best combined calibration among the reported comparators (Winkler = 0.0364), although matched ablations do not identify the Regime Uncertainty Gate as the source of that advantage: a posterior-independent learned global scale performs at least as well in both ablation batches. The dual-lens interpretability framework reveals that regime conditioning produces qualitatively different—not merely scaled—factor structures across market states.

#### 5.10.2 Practical implications

The model targets settings where distributional accuracy outweighs point-forecast direction: risk budgeting, portfolio construction, and regulatory capital estimation. Prediction intervals adapt to regime confidence, and regime-decomposed explanations support human-in-the-loop oversight. The costs are real and not training-only: relative to the Vanilla TFT, RC-Mamba requires 2.8× the epoch time and 3.2× the per-sample inference latency, against 15% fewer parameters (Section 4.9).

#### 5.10.3 Future work

The limitations identified in Section 5.9 point to five extensions:

1. **Mamba-2 parallel scan**. Adopting the parallel scan of Mamba-2 (Dao and Gu, 2024) could reduce training-time overhead while preserving the regime-conditioning mechanism.
2. **Continuous regime dynamics**. Replacing the discrete HMM with a continuous latent model (e.g., a neural SDE or normalizing flow) would capture finer regime gradients at the cost of added complexity.
3. **Multi-market macro observables**. Adding local monetary policy indicators (ECB rates, SHIBOR) and regional economic series could improve forecasts for non-US assets.
4. **Intraday extension**. Higher-frequency data requires redesigning the HMM observables (VIX and credit spreads are inherently daily) and managing longer sequences in the Mamba recurrence.
5. **Cross-asset dependency modeling**. Graph neural network overlays or cross-attention between asset streams would enable explicit modeling of correlations, contagion, and lead-lag effects.

These directions aim to extend regime conditioning toward a broader framework for multi-asset, multi-frequency probabilistic forecasting. More broadly, conditioning a selective-SSM recurrence on latent-regime posteriors is an architectural mechanism whose cross-domain transfer we do not test here and leave to future work. In principle any non-stationary sequence-modeling task with estimable latent regimes—for example, clinical monitoring, industrial fault detection, or climate time series—could adopt the same construction by replacing the macro-financial observables with domain-appropriate regime indicators.

## Data Availability

Publicly available datasets were analyzed in this study. This data can be found at: https://fred.stlouisfed.org/ and https://finance.yahoo.com/.

## References

[B1] Ahamed M. A. Cheng Q. (2024). “TimeMachine: a time series is worth 4 mambas for long-term forecasting,” in European conference on artificial intelligence (ECAI) (Santiago de Compostela). doi: 10.3233/FAIA240677 39866204 PMC11767608

[B2] Ang A. Bekaert G. (2002). International asset allocation with regime shifts. Rev. Financ. Stud. 15, 1137–1187. doi: 10.1093/rfs/15.4.1137

[B3] Bollerslev T. (1986). Generalized autoregressive conditional heteroskedasticity. J. Econom. 31, 307–327. doi: 10.1016/0304-4076(86)90063-1

[B4] Bulla J. Mergner S. Bulla I. Sesboüé A. Chesneau C. (2011). Markov-switching asset allocation: do profitable strategies exist? J. Asset Manag. 12, 310–321. doi: 10.1057/jam.2010.27

[B5] Dao T. Gu A. (2024). “Transformers are SSMs: generalized models and efficient algorithms through structured state space duality,” in International conference on machine learning (ICML) (Vienna).

[B6] Diebold F. X. Mariano R. S. (1995). Comparing predictive accuracy. J. Bus. Econ. Stat. 13, 253–263. doi: 10.1080/07350015.1995.10524599

[B7] Engle R. F. (1982). Autoregressive conditional heteroscedasticity with estimates of the variance of United Kingdom inflation. Econometrica 50, 987–1007. doi: 10.2307/1912773

[B8] Gneiting T. Raftery A. E. (2007). Strictly proper scoring rules, prediction, and estimation. J. Am. Stat. Assoc. 102, 359–378. doi: 10.1198/016214506000001437

[B9] Gray S. F. (1996). Modeling the conditional distribution of interest rates as a regime-switching process. J. Financ. Econ. 42, 27–62. doi: 10.1016/0304-405X(96)00875-6

[B10] Gu A. Dao T. (2023). Mamba: linear-time sequence modeling with selective state spaces. arXiv [Preprint]. arXiv:2312.00752. doi: 10.48550/arXiv.2312.00752

[B11] Gu A. Goel K. Ré C. (2022). “Efficiently modeling long sequences with structured state spaces,” in International conference on learning representations (ICLR) (Virtual).

[B12] Gu S. Kelly B. Xiu D. (2020). Empirical asset pricing via machine learning. Rev. Financ. Stud. 33, 2223–2273. doi: 10.1093/rfs/hhaa009

[B13] Guidolin M. Timmermann A. (2007). Asset allocation under multivariate regime switching. J. Econ. Dyn. Control 31, 3503–3544. doi: 10.1016/j.jedc.2006.12.004

[B14] Hamilton J. D. (1989). A new approach to the economic analysis of nonstationary time series and the business cycle. Econometrica 57, 357–384. doi: 10.2307/1912559

[B15] Hamilton J. D. Susmel R. (1994). Autoregressive conditional heteroskedasticity and changes in regime. J. Econom. 64, 307–333. doi: 10.1016/0304-4076(94)90067-1

[B16] Hochreiter S. Schmidhuber J. (1997). Long short-term memory. Neural Comput. 9, 1735–1780. doi: 10.1162/neco.1997.9.8.1735 9377276

[B17] Kim C.-J. (1994). Dynamic linear models with Markov-switching. J. Econom. 60, 1–22. doi: 10.1016/0304-4076(94)90036-1

[B18] Lim B. Arık S. Ö. Loeff N. Pfister T. (2021). Temporal fusion transformers for interpretable multi-horizon time series forecasting. Int. J. Forecast. 37, 1748–1764. doi: 10.1016/j.ijforecast.2021.03.012

[B19] Liu Y. Hu T. Zhang H. Wu H. Wang S. Ma L. . (2024). “iTransformer: inverted transformers are effective for time series forecasting,” in International conference on learning representations (ICLR) (Vienna).

[B20] López de Prado M. (2018). Advances in Financial Machine Learning. Hoboken, NJ: Wiley.

[B21] Loshchilov I. Hutter F. (2019). “Decoupled weight decay regularization,” in International conference on learning representations (ICLR) (New Orleans, LA).

[B22] Lundberg S. M. Lee S.-I. (2017). “A unified approach to interpreting model predictions,” in Advances in neural information processing systems (NeurIPS) (Long Beach, CA), 4765–4774.

[B23] Matheson J. E. Winkler R. L. (1976). Scoring rules for continuous probability distributions. Manage. Sci. 22, 1087–1096. doi: 10.1287/mnsc.22.10.1087

[B24] Nie Y. Nguyen N. H. Sinthong P. Kalagnanam J. (2023). “A time series is worth 64 words: long-term forecasting with transformers,” in International conference on learning representations (ICLR) (Kigali).

[B25] Oreshkin B. N. Carpov D. Chapados N. Bengio Y. (2020). “N-BEATS: neural basis expansion analysis for interpretable time series forecasting,” in International Conference on Learning Representations (ICLR) (Virtual).

[B26] Patro B. N. Agneeswaran V. S. (2024). SiMBA: simplified Mamba-based architecture for vision and multivariate time series. arXiv [Preprint]. arXiv:2403.15360. doi: 10.48550/arXiv.2403.15360

[B27] Rangapuram S. S. Seeger M. W. Gasthaus J. Stella L. Wang Y. Januschowski T. (2018). “Deep state space models for time series forecasting,” in Advances in neural information processing systems (NeurIPS) (Montréal, QC), 7796–7805.

[B28] Rasul K. Ashok A. Williams A. R. Khorasani A. Adamopoulos G. Bhagwatkar R. . (2023). Lag-Llama: towards foundation models for probabilistic time series forecasting. arXiv [Preprint]. arXiv:2310.08278. doi: 10.48550/arXiv.2310.08278

[B29] Rasul K. Seward C. Schuster I. Vollgraf R. (2021). “Autoregressive denoising diffusion models for multivariate probabilistic time series forecasting,” in International conference on machine learning (ICML) (Virtual), 8857–8868.

[B30] Ribeiro M. T. Singh S. Guestrin C. (2016). “‘Why should I trust you?: explaining the predictions of any classifier,” in Proceedings of the 22nd ACM SIGKDD international conference on knowledge discovery and data mining (KDD) (San Francisco, CA), 1135–1144. doi: 10.1145/2939672.2939778

[B31] Romano Y. Patterson E. Candès E. J. (2019). “Conformalized quantile regression,” in Advances in neural information processing systems (NeurIPS) (Vancouver, BC), 3543–3553.

[B32] Rousseeuw P. J. Croux C. (1993). Alternatives to the median absolute deviation. J. Am. Stat. Assoc. 88, 1273–1283. doi: 10.1080/01621459.1993.10476408

[B33] Rydén T. Teräsvirta T. Åsbrink S. (1998). Stylized facts of daily return series and the Hidden Markov model. J. Appl. Econ. 13, 217–244. doi: 10.1002/(SICI)1099-1255(199805/06)13:3<217::AID-JAE476>3.0.CO;2-V

[B34] Salinas D. Flunkert V. Gasthaus J. Januschowski T. (2020). DeepAR: probabilistic forecasting with autoregressive recurrent networks. Int. J. Forecast. 36, 1181–1191. doi: 10.1016/j.ijforecast.2019.07.001

[B35] Soliman W. Chen Z. Johnson C. Wong S. (2023). ETF markets' prediction and assets management platform using probabilistic autoregressive recurrent networks. Eurasia Proc. Sci. Technol. Eng. Math. 23, 485–494. doi: 10.55549/epstem.1372067

[B36] Soliman W. Chen Z. Johnson C. Wong S. (2025). Improving ETF prediction through sentiment analysis: a DeepAR and FinBERT approach with controlled seed sampling. Intell. Syst. Account. Financ. Manag. 32:e70004. doi: 10.1002/isaf.70004

[B37] Vaswani A. Shazeer N. Parmar N. Uszkoreit J. Jones L. Gomez A. N. . (2017). “Attention is all you need,” in Advances in neural information processing systems (NeurIPS) (Long Beach, CA), 5998–6008.

[B38] Wang S. Wu H. Shi X. Hu T. Luo H. Ma L. . (2024). “TimeMixer: decomposable multiscale mixing for time series forecasting,” in International conference on learning representations (ICLR) (Vienna).

[B39] Wang Z. Kong F. Feng S. Wang M. Yang X. Zhao H. . (2025). Is Mamba effective for time series forecasting? Neurocomputing 619:129178. doi: 10.1016/j.neucom.2024.129178

[B40] Winkler R. L. (1972). A decision-theoretic approach to interval estimation. J. Am. Stat. Assoc. 67, 187–191. doi: 10.1080/01621459.1972.10481224

[B41] Wu H. Hu T. Liu Y. Zhou H. Wang J. Long M. (2023). “TimesNet: temporal 2D-variation modeling for general time series analysis,” in International conference on learning representations (ICLR) (Kigali).

[B42] Wu H. Xu J. Wang J. Long M. (2021). “Autoformer: decomposition transformers with auto-correlation for long-term series forecasting,” in Advances in neural information processing systems (NeurIPS) (Virtual), 22419–22430.

[B43] Zhou H. Zhang S. Peng J. Zhang S. Li J. Xiong H. . (2021). Informer: beyond efficient transformer for long sequence time-series forecasting. Proc AAAI Conf Artif Intell. 35, 11106–11115. doi: 10.1609/aaai.v35i12.17325

