# Supplementary material for: RC-Mamba: regime-conditioned selective state-space models for probabilistic time-series forecasting
Source: Front Artif Intell. 2026 Sep 23;9:1903497. doi: 10.3389/frai.2026.1903497 (PMC13642593; doi:10.3389/frai.2026.1903497)
Supplement: Supplementary file 1 [file Supplementary_file_1.pdf]

Supplementary Material

RC-Mamba: Regime-Conditioned Selective State-Space Models
for Probabilistic Time-Series Forecasting

Appendices

S1 Per-Fold Detailed Results

Tables S1–S5 report per-fold metrics for all seven models under the revised calendar-aligned walk-forward protocol, with a 30-business-day embargo and common preprocessing and training budget. Hyperparameters were tuned once on Fold 0 and reused across all folds.

Table S1: Fold 0 results (test year 2021).

Model	CRPS	MAE	RMSE	Dir %	$\mathbf{PICP_{80}}$ (%)	$\mathbf{Winkler_{80}}$
RC-Mamba	0.00432	0.00588	0.00860	52.5	83.6	0.02887
VanillaTFT	0.00509	0.00619	0.00874	46.4	94.3	0.03924
DeepAR	0.00485	0.00595	0.00865	52.2	92.3	0.03676
S-Mamba	0.00530	0.00684	0.00931	47.9	92.6	0.03853
HMM-DeepAR	0.00659	0.00915	0.01146	46.5	93.4	0.04553
LSTM	0.00467	0.00629	0.00885	47.8	79.1	0.03203
N-BEATS	0.00505	0.00631	0.00914	46.7	52.9	0.03969

Table S2: Fold 1 results (test year 2022).

Model	CRPS	MAE	RMSE	Dir %	$\mathbf{PICP_{80}}$ (%)	$\mathbf{Winkler_{80}}$
RC-Mamba	0.00767	0.01031	0.01455	47.8	60.9	0.05311
VanillaTFT	0.00762	0.01033	0.01458	46.2	71.0	0.05231
DeepAR	0.00781	0.01044	0.01465	46.2	83.8	0.05430
S-Mamba	0.00780	0.01046	0.01474	50.6	75.9	0.05432
HMM-DeepAR	0.00904	0.01044	0.01486	53.1	44.6	0.07475
LSTM	0.00977	0.01079	0.01513	50.7	66.5	0.08074
N-BEATS	0.22231	0.23612	0.36607	48.7	14.8	2.09857

Table S3: Fold 2 results (test year 2023).

Model	CRPS	MAE	RMSE	Dir %	$\mathbf{PICP_{80}}$ (%)	$\mathbf{Winkler_{80}}$
RC-Mamba	0.00485	0.00670	0.00913	49.5	72.8	0.03144
VanillaTFT	0.00515	0.00671	0.00914	49.7	91.8	0.03650
DeepAR	0.00594	0.00797	0.01166	49.3	89.0	0.04035
S-Mamba	0.00610	0.00827	0.01075	49.3	89.5	0.04161
HMM-DeepAR	0.00621	0.00807	0.01128	49.3	95.0	0.04516
LSTM	0.03844	0.01835	0.02552	48.8	95.4	0.41890
N-BEATS	0.26331	0.01978	0.03316	49.1	84.4	3.09717

Table S4: Fold 3 results (test year 2024).

**Model**	**CRPS**	**MAE**	**RMSE**	**Dir %**	$\mathbf{PICP_{80}}$ **(%)**	$\mathbf{Winkler_{80}}$
RC-Mamba	0.00489	0.00630	0.00948	51.2	92.4	0.03565
VanillaTFT	0.00510	0.00630	0.00947	52.6	92.8	0.03870
DeepAR	0.00498	0.00632	0.00949	50.7	90.8	0.03706
S-Mamba	0.00588	0.00781	0.01073	46.7	91.6	0.04154
HMM-DeepAR	0.00550	0.00721	0.01021	53.2	91.9	0.04037
LSTM	0.00499	0.00669	0.00975	45.1	81.8	0.03448
N-BEATS	0.03907	0.01936	0.08781	47.9	21.4	0.42505

Table S5: Fold 4 results (test year 2025).

**Model**	**CRPS**	**MAE**	**RMSE**	**Dir %**	$\mathbf{PICP_{80}}$ **(%)**	$\mathbf{Winkler_{80}}$
RC-Mamba	0.00488	0.00663	0.01065	52.7	80.7	0.03295
VanillaTFT	0.00496	0.00666	0.01069	50.8	84.4	0.03436
DeepAR	0.00562	0.00738	0.01113	44.1	88.6	0.04056
S-Mamba	0.00631	0.00743	0.01122	51.6	95.7	0.05097
HMM-DeepAR	0.00530	0.00673	0.01071	54.5	89.2	0.03945
LSTM	0.00526	0.00678	0.01073	54.2	68.6	0.03939
N-BEATS	0.00870	0.00671	0.01078	53.6	77.9	0.08168

S2 HMM Regime Diagnostics

Under the revised protocol the 3-state Student-t HMM is refitted independently on each fold's training partition, so five distinct fits exist and none observes data at or beyond its fold's embargo boundary. The diagnostics below are for the final fold (test year 2025) unless stated otherwise. The HMM uses three observables: VIXCLS, BAMLH0A0HYM2, and DGS10 − DGS2, standardised on the fitting fold's training window; emission means are therefore reported in standardised units and are not directly comparable across folds. Fold boundaries are given in Table S17.

Transition matrix. The row-stochastic matrix for the final fold exhibits sticky behaviour with self-transition probabilities of $\sim$0.905:

$$\mathbf{A}^{(4)} = \begin{pmatrix} 0.905 & 0.047 & 0.047 \\ 0.047 & 0.906 & 0.047 \\ 0.047 & 0.047 & 0.905 \end{pmatrix}.$$

Stickiness increases monotonically with the length of the training window: the mean self-transition probability is 0.874, 0.880, 0.885, 0.886 and 0.905 for folds 0 to 4 respectively, giving implied mean regime durations $1/(1 - a_{kk})$ of 8.0 to 10.6 business days. Figure 6(b) of the main text plots the across-fold average, whose diagonal is 0.886.

State characteristics. Table S6 summarises the macro profile of each regime.

Table S6: HMM state statistics for the final fold (test year 2025). Occupancy is the share of that fold's 221 forecast origins decoded to each state, and emission means are in the standardised coordinates in which the fold-local HMM is fitted. Expected duration is $1/(1 - a_{kk})$ in business days. The final fold decodes no origin to Transition; pooled over all five folds the shares are 33.2 % Calm, 2.5 % Transition and 64.3 % Stress, so Transition is a rare decode throughout and not an artefact of this fold.

State	Occupancy (%)	Self-transition	Duration (d)	VIX	HY spread	Term spread
Calm	74.2	0.905	10.6	-1.81	-2.05	-2.02
Transition	0.0	0.906	10.6	-0.23	-0.10	-0.15
Stress	25.8	0.905	10.6	+1.76	+2.08	+1.87

Known-period validation. The April 2025 stress episode (3 Apr–8 May 2025) lies inside the fold-4 test block. The window spans 26 business days, of which 25 are trading days (18 April was a market holiday), and the HMM decodes all 25 to State 2 (Stress), a classification accuracy of 100 % on the observed days. No low-volatility counterpart is validated from 2026: under the calendar-aligned protocol the test origins end on 2025-11-17, so 2026 dates fall outside the evaluated sample.

Regime posterior timeline. Figure S1 shows the causal posterior regime probabilities at every evaluated forecast origin, confirming persistent state assignments with sharp transitions during known market dislocations.

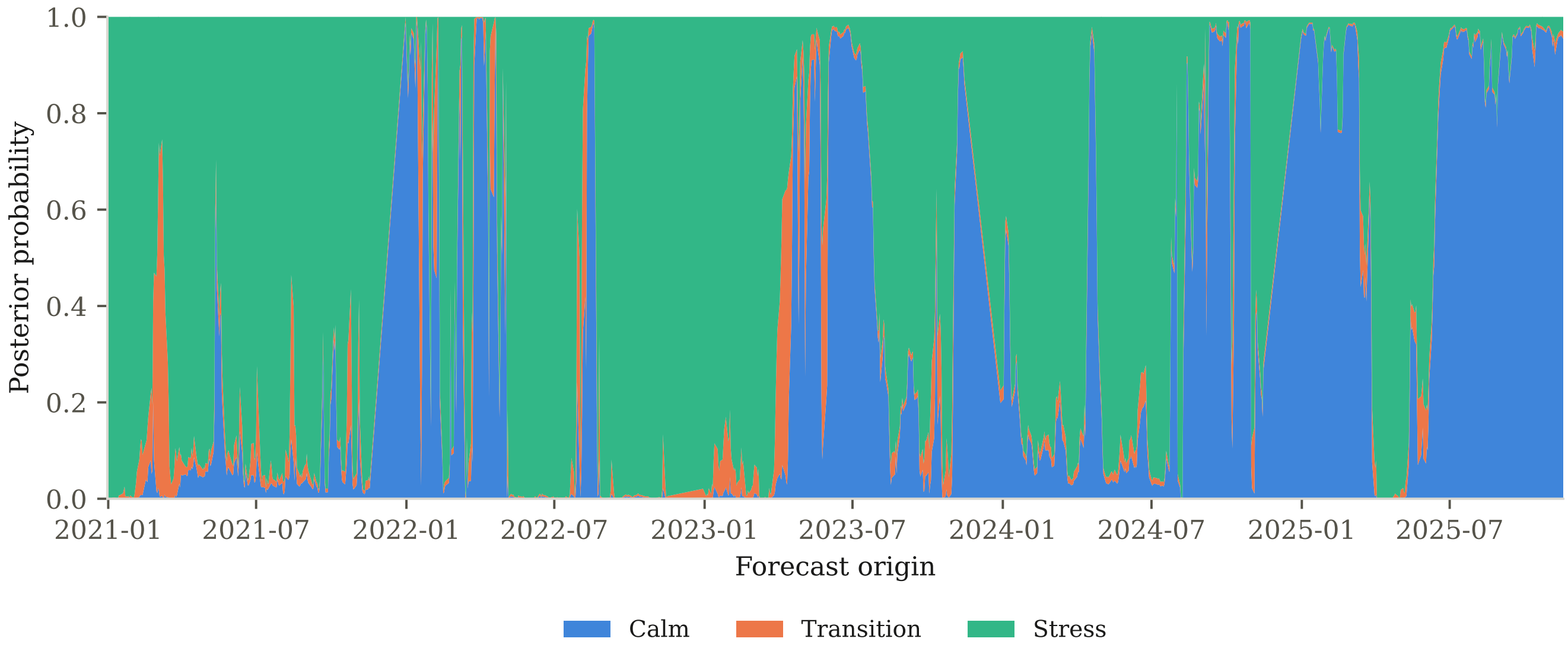

Figure S1: Causal HMM regime posteriors at the 1,110 evaluated forecast origins (2020-12-31 to 2025-11-17), concatenated across the five fold-local fits. Each origin is decoded by the HMM belonging to its own fold, using information available at that date only. The April 2025 tariff episode is visible as the sustained Stress block near the right-hand edge. Earlier history is used for fitting but is not plotted, because no forecast is evaluated there.

S3 SHAP Feature Attribution by Ticker and Regime

Attributions come from the grouped permutation SHAP analysis of the main text: 128 samples drawn across all five final checkpoints, all 18 ETFs and all three decoded regimes, with 64 antithetic permutations per sample and each feature carrying all 24 of its lags as a single player. We report per-regime top features (Table S7) and per-ticker top-3 features (Table S8). Per-ticker profiles rest on 5–10 samples each and are descriptive.

Table S7: Top-5 SHAP features per regime from the revised post-hoc permutation analysis, ranked by mean $|\phi_j|$ within each state.

**Regime**	**Top-5 features by mean** $|\phi_j|$
Calm	`EMVOVERALLEMV`, `RBUSBIS`, `GDPC1`, `FEDFUNDS`, `WALCL`
Transition	`WALCL`, `PAYEMS`, `EMVMACROBUS`, `RBUSBIS`, `VIXCLS`
Stress	`EMVOVERALLEMV`, `EMVMACROBUS`, `FEDFUNDS`, `RBUSBIS`, `PAYEMS`

Table S8: Top-3 SHAP features per ETF from the revised post-hoc permutation analysis, ranked by mean $|\phi_j|$ within each asset.

**Ticker**	**Top-3 features by mean** $|\phi_j|$
BND	`FEDFUNDS`, `EMVOVERALLEMV`, `IRLTLT01USM156N`
CEMB	`EMVMACROBUS`, `EMVOVERALLEMV`, `RBUSBIS`
EMXC	`Volume`, `WALCL`, `EMVOVERALLEMV`
EWG	`RBUSBIS`, `PAYEMS`, `EMVMACROINTEREST`
EWH	`RBUSBIS`, `WALCL`, `PAYEMS`
EWQ	`PAYEMS`, `EMVOVERALLEMV`, `WALCL`
EWU	`EMVOVERALLEMV`, `RBUSBIS`, `WALCL`
FXI	`EMVOVERALLEMV`, `RBUSBIS`, `DGS3MO`
GLD	`DGS3MO`, `EMVMACROINTEREST`, `RBUSBIS`
GOVT	`EMVOVERALLEMV`, `EMVMACROBUS`, `CPALTT01USM661S`
IGOV	`FEDFUNDS`, `EMVOVERALLEMV`, `DGS3MO`
IVOO	`EMVOVERALLEMV`, `FEDFUNDS`, `CPALTT01USM661S`
JNK	`EMVOVERALLEMV`, `EMVMACROINTEREST`, `DGS3MO`
SPY	`WALCL`, `RBUSBIS`, `EMVOVERALLEMV`
VGK	`EMVMACROBUS`, `EMVOVERALLEMV`, `RBUSBIS`
VIOO	`FEDFUNDS`, `EMVOVERALLEMV`, `DGS3MO`
VOO	`H-L`, `WALCL`, `VIXCLS`
VWOB	`WALCL`, `DFII10`, `RBUSBIS`

S4 Computational Complexity and Efficiency

This appendix reports the asymptotic computational complexity of the RC-Mamba block and the empirical training-time overhead of the full architecture relative to a Vanilla TFT of equivalent capacity. The two are reported separately because they answer different questions: asymptotic complexity characterises how the architecture scales with sequence length, while the empirical overhead reflects the cost paid on the experimental configuration of Section 3.10 on the hardware described in Section 3.10.16.

S4.1 Asymptotic Complexity

The dominant cost in the RC-Mamba block is the sequential SSM scan of Equations (25)–(26). Because the SSM is diagonal, each step performs only $O(d_{\text{state}})$ element-wise operations, and the scan over the combined sequence of length $L = T + H$ is $O(L\, d_{\text{state}})$ in time. The depth-wise causal convolution of Equation (17) adds an $O(L\, d_{\text{inner}}\, k)$ term with $k = 3$, also linear in L. The regime-conditioned SSM parameter projections of Equations (18)–(21) contribute $O(L\, d_{\text{state}}\, (d_{\text{inner}} + K))$ per block. With the selected-configuration constants $d_{\text{state}} = 16$, $d_{\text{inner}} = 32$, $K = 3$, the per-block per-step cost is strictly linear in L and small in absolute terms. The Student-t HMM is $O(TK^2)$ per training step for the forward filter and $O(HK^2)$ for the future propagation; with $K = 3$, both are negligible relative to the deep-network cost.

This contrasts with the multi-head self-attention layer of the Vanilla TFT, whose cost scales as $O(L^2\, d_{\text{model}})$ in time and $O(L^2)$ in memory for the attention matrix — both quadratic in the sequence length. At the values of L used in this paper ($L = T + H = 54$), the quadratic term in the TFT is small enough that empirical wall-clock time is dominated by other components, and the

asymptotic advantage of RC-Mamba is not yet operative. The benefit of linear sequence scaling would become decisive only at look-back lengths an order of magnitude larger than those used here, which we flag as a future-work direction in Section 5.10.

S4.2 Empirical Training and Inference Cost

Cost is measured by synchronised profiling rather than by end-to-end job timing, so that every model is compared under the same loader, batch size and input shapes (50 profiled repetitions after 10 warm-up steps, on a single NVIDIA GeForce RTX 3060 Laptop GPU with 6 GB VRAM). On that basis RC-Mamba requires 224.4 s per epoch-equivalent pass against 80.0 s for the Vanilla TFT baseline of Section 3.10.6, a factor of 2.8×, and 1.158 ms per sample at inference against 0.361 ms, a factor of 3.2×. Peak CUDA allocation is 208.2 MiB for RC-Mamba against 231.3 MiB for the Vanilla TFT, so the memory comparison favours RC-Mamba by 10.0 %, and RC-Mamba is the more compact model (697 K against 816 K parameters).

The training-time overhead is concentrated in three places. First, the four-stage training protocol of Section 3.9 performs 11,450 gradient steps per fold against the substantially shorter single-stage schedule used by the TFT baseline. Second, the sequential SSM scan does not benefit from the same level of GPU parallelisation as the matrix-multiply-heavy attention block of the TFT, even though both are linear in the per-step cost. Third, the Stage 4 CRPS calibration phase performs $S = 200$ Monte-Carlo samples per training example, which the TFT does not incur. These costs are not intrinsic to the asymptotic sequence-length scaling of the architecture, although they are real costs in the present implementation; adopting the parallel scan of Mamba-2 (Dao and Gu, 2024) could reduce part of the training-time gap while preserving the regime-conditioning mechanism, although we have not yet benchmarked a Mamba-2 implementation in this work.

Inference is *not* comparable to the TFT. Under the same profiling contract RC-Mamba needs 1.158 ms per sample against 0.361 ms for the Vanilla TFT, so the overhead is not a training-only cost and cannot be amortised by retraining less often. The gap has the same origin as the training gap — the sequential selective scan does not use the device's parallelism — and it is the stronger argument for a parallel-scan implementation. A single forward pass with the inference-time Monte-Carlo budget of $S = 1{,}000$ samples still completes in well under a second on the documented hardware, so the model remains deployable in streaming settings, but we do not claim inference parity with the transformer baseline.

Table S9: Asymptotic time complexity per block, per time step. $L = T + H$ is the combined sequence length, K the HMM regime count. RC-Mamba is linear in L; the TFT attention layer is quadratic.

**Component**	**Time**	**Memory**
RC-Mamba SSM scan	$O(L\, d_{\text{state}})$	$O(d_{\text{state}})$
RC-Mamba causal conv.	$O(L\, d_{\text{inner}}\, k)$	$O(d_{\text{inner}})$
RC-Mamba SSM proj.	$O(L\, d_{\text{state}}\, (d_{\text{inner}} + K))$	$O(d_{\text{state}}\, d_{\text{inner}})$
HMM forward filter	$O(TK^2)$	$O(K)$
TFT self-attention	$O(L^2\, d_{\text{model}})$	$O(L^2)$

S5 Additional Methodological Detail

This section holds standard-methods material referenced from the manuscript: formal definitions of the evaluation metrics, the Gated Residual Network and Variable Selection Network formulations, the input-feature definitions, and the implementation and reproducibility record. Sources are named by author and year; the corresponding entries appear in the manuscript's reference list.

S5.1 Evaluation Metric Definitions

All metrics are computed per asset and per fold, then aggregated as mean ± standard deviation across the five walk-forward folds.

Continuous ranked probability score. CRPS (Gneiting and Raftery, 2007) is a strictly proper scoring rule that penalises calibration error and lack of sharpness simultaneously, which is why we adopt it as the primary metric:

$$\mathrm{CRPS}(F, y) = \int_{-\infty}^{\infty} \Big(F(z) - \mathbb{1}[z \geq y] \Big)^2 \, \mathrm{d}z, \tag{S1}$$

where F is the predictive CDF and y the realised value. For the Student-t predictive distribution there is no closed form, so CRPS is estimated by Monte Carlo with S=1,000 draws at evaluation time using the energy representation $\mathbb{E}|X - y| - \frac{1}{2}\mathbb{E}|X - X'|$ for independent draws X, X' from the predictive law. Lower is better.

Point accuracy. Three point metrics are reported, all computed from the median forecast $\hat{y}^{0.50}$: root mean squared error (RMSE), which penalises large errors; mean absolute error (MAE), a robust measure of central-tendency accuracy; and directional accuracy, the percentage of steps at which the sign of the predicted return matches the sign of the realised return, where values above 50 % indicate better-than-random direction calling.

Interval calibration. PICP_α (prediction interval coverage probability) is the empirical fraction of observations falling inside the nominal α % interval; a well-calibrated model attains $\mathrm{PICP}_{80} \approx 0.80$. PINAW_α (prediction interval normalised average width) is the mean interval width divided by the range of observed values, where lower is better conditional on achieving target coverage. Because coverage and width trade off against each other, neither is a calibration criterion on its own, and we rank on the Winkler interval score (Winkler, 1972), which penalises both:

$$W_\alpha = \frac{1}{n} \sum_{i=1}^{n} \begin{cases} (U_i - L_i) + \frac{2}{\alpha}(L_i - y_i) & \text{if } y_i < L_i, \\ (U_i - L_i) & \text{if } L_i \leq y_i \leq U_i, \\ (U_i - L_i) + \frac{2}{\alpha}(y_i - U_i) & \text{if } y_i > U_i, \end{cases} \tag{S2}$$

with L_i and U_i the lower and upper bounds of the α % interval. We report W_{80}. As an additional check, normalised conformal calibration (Romano et al., 2019) is applied per ticker and per fold at target coverage levels $\{50, 80, 90\}$ % to assess calibration beyond the model's native intervals.

S5.2 Gated Residual Network and Variable Selection Network

Both modules are adopted without modification from Lim et al. (2021) and are reproduced here for completeness; panels (a) and (b) of Supplementary Figure S3 illustrate them.

Gated Residual Network. Given an input $\mathbf{x} \in \mathbb{R}^{d_{\text{in}}}$ and an optional context vector $\mathbf{c}$:

$$\boldsymbol{\eta}_1 = \mathbf{W}_1\mathbf{x} + \mathbf{b}_1 + \mathbb{1}[\mathbf{c} \neq \varnothing]\, \mathbf{W}_c\, \mathbf{c}, \tag{S3}$$

$$\boldsymbol{\eta}_2 = \mathrm{ELU}(\boldsymbol{\eta}_1), \tag{S4}$$

$$\boldsymbol{\eta}_3 = \mathbf{W}_2\boldsymbol{\eta}_2 + \mathbf{b}_2, \quad \boldsymbol{\eta}_3 \in \mathbb{R}^{2d_{\text{out}}}, \tag{S5}$$

followed by a gated linear unit (GLU) and a residual connection:

$$[\mathbf{a},\, \mathbf{b}] = \mathrm{split}\Big(\mathrm{Dropout}(\boldsymbol{\eta}_3)\Big), \tag{S6}$$

$$\mathrm{GLU}(\boldsymbol{\eta}_3) = \mathbf{a} \odot \sigma(\mathbf{b}), \tag{S7}$$

$$\mathrm{GRN}(\mathbf{x}, \mathbf{c}) = \mathrm{LayerNorm}\Big(\mathrm{GLU}(\boldsymbol{\eta}_3) + \mathrm{skip}(\mathbf{x})\Big), \tag{S8}$$

where $\mathrm{skip}(\mathbf{x}) = \mathbf{W}_s\mathbf{x}$ when $d_{\text{in}} \neq d_{\text{out}}$ and the identity otherwise. Context conditioning $\mathbf{W}_c\mathbf{c}$ is bias-free and is applied additively before the activation.

Variable Selection Network. For N input variables, each independently projected to d_{var} dimensions, the VSN selects among them with learned softmax weights:

$$\boldsymbol{\xi}_j = \mathrm{GRN}_j(\mathbf{x}_j,\ \mathbf{c}), \quad j = 1, \ldots, N, \tag{S9}$$

$$\mathbf{v} = \mathrm{softmax}\Big(\mathrm{GRN}_{\text{sel}}\Big([\mathbf{x}_1; \ldots; \mathbf{x}_N],\ \mathbf{c}\Big)\Big) \in \Delta^{N-1}, \tag{S10}$$

$$\mathrm{VSN}(\mathbf{x}, \mathbf{c}) = \sum_{j=1}^{N} v_j\, \boldsymbol{\xi}_j \in \mathbb{R}^{d_{\text{model}}}. \tag{S11}$$

The weights $\mathbf{v}$ are directly interpretable as variable-importance scores, and are the quantity reported as intrinsic VSN importance in the manuscript. For the historical VSN N=30 and the sequence length is T; for the future VSN N=3 and the sequence length is H.

S5.3 Input Feature Definitions

The model ingests $V_h = 30$ historical exogenous features organised into four thematic groups — price and volume statistics, technical indicators, macro-financial series, and sentiment aggregates. Supplementary Table S16 lists every feature with its data source and publication lag; those lags are respected throughout walk-forward evaluation so that no observation enters the model before it was available in practice.

Three calendar features serve as known-future covariates ($V_f = 3$): `day_of_week`, `week_of_year` and `month`. These are deterministically known at forecast time and are supplied as raw, unnormalised integer encodings.

The Hidden Markov Model operates on a separate observation vector $\mathbf{o}_t \in \mathbb{R}^3$:

1. `VIXCLS` — CBOE Volatility Index, capturing implied equity volatility;
2. `BAMLH0A0HYM2` — ICE BofA US High-Yield option-adjusted spread, capturing credit risk;
3. `DGS10−DGS2` — 10-year minus 2-year Treasury yield spread, capturing the slope of the term structure.

These three were chosen to span complementary dimensions of market regime — equity risk, credit stress, and monetary and growth expectations — and all three are published without lag, so they are available at every forecast origin.

S5.4 Implementation, Hardware and Reproducibility

The selected RC-Mamba architecture is specified in full in Supplementary Table S19, and the stage-wise training hyperparameters in Supplementary Table S15. Training uses batch size 64 and a fixed random seed of 42. Stage 2 uses a learning rate of $\eta = 3 \times 10^{-3}$ decayed by cosine annealing with a 50-step linear warmup to a floor of 10^{-6}; joint fine-tuning (Stage 3) uses $\eta \approx 10^{-4}$ and CRPS fine-tuning (Stage 4) uses $\eta \approx 10^{-5}$.

All experiments were run on a single NVIDIA GeForce RTX 3060 Laptop GPU (6 GB VRAM) using PyTorch. The RC-Mamba model, the HMM, the training pipeline and every baseline are implemented in a custom codebase without reliance on an external forecasting library. Measured wall-clock and memory cost is reported in the manuscript's computational-efficiency section and in Section S4.2 below.

For reproducibility, all random number generators (Python, NumPy, PyTorch and CUDA) are seeded with the same fixed value of 42, and deterministic cuDNN and cuBLAS settings are enforced. Model checkpoints are written at the best validation loss of each stage. The complete configuration — architecture, training, evaluation and data-processing parameters — is serialised to YAML, and the walk-forward split indices, feature normalisation statistics and fitted HMM parameters are logged per fold, so every reported result can be reproduced exactly from the released artifacts.

S6 Extended Statistical Robustness

S6.1 Primary and Secondary Inference Agree

The main text reports a moving-block bootstrap over 1,110 synchronized forecast origins as the primary test, because pooling the 599,400 individual prediction cells treats overlapping horizons, adjacent origins and cross-sectionally correlated assets as independent and therefore overstates the effective sample size. Table S10 shows that the conservative primary test and a Diebold–Mariano test with Newey–West HAC variance estimation at lag 29 reach the same conclusion for every retained comparison, so the reported significance does not depend on the choice of procedure.

Table S10: Agreement between the primary origin-level moving-block bootstrap and the secondary HAC-adjusted Diebold–Mariano test. Both columns are Holm-adjusted across the reported family.

**Comparison**	**Bootstrap p_{Holm}**	**HAC-DM p_{Holm}**	**Same conclusion**
RC-Mamba vs. VanillaTFT	0.0060	$< 10^{-4}$	yes
RC-Mamba vs. DeepAR	0.0060	$< 10^{-4}$	yes
RC-Mamba vs. S-Mamba	0.0060	$< 10^{-4}$	yes
RC-Mamba vs. HMM-DeepAR	0.0060	$< 10^{-4}$	yes
RC-Mamba vs. LSTM	0.0060	0.0011	yes
RC-Mamba vs. N-BEATS	0.0060	$< 10^{-4}$	yes

S6.2 Performance by Market State

Because the paper's mechanism is regime conditioning, it is natural to ask whether its benefit concentrates in stressed markets. Table S11 compares RC-Mamba against the matched no-regime control conditional on the market state decoded at the forecast origin, pooling all five folds. The regime pathway does not deliver a state-dependent accuracy gain: the control is marginally ahead in both Calm and Stress origins, and RC-Mamba leads only in Transition origins, which account for 2.5 % of the sample. None of the three differences is significant.

What does replicate across every state is interval coverage. RC-Mamba is closer to the nominal 80 % level in all three regimes, by a near-constant 3.5 percentage points, because the regime pathway produces systematically wider predictive intervals. Pooled over the 1,110 origins the mean 80 % interval width is 0.0235 against 0.0202 for the control, i.e. 16.7 % wider (moving-block bootstrap 95 % CI on the difference [0.0024, 0.0044], $p = 0.001$). Under Winkler$_{80}$, which penalises width and miscoverage jointly, that exchange is approximately neutral: the difference of 0.0005 is not significant ($p = 0.33$). We therefore describe the mechanism as calibration-oriented rather than accuracy-improving, consistent with the matched ablation evidence in the main text.

Table S11: RC-Mamba versus the matched no-regime control, conditional on the market state decoded at the forecast origin. Losses are averaged within each origin over 18 assets and 30 horizons before comparison; percentages are RC-Mamba's relative CRPS advantage. *Width* is the mean 80 % prediction-interval width.

**State at origin**	**Origins**	**Share (%)**	**RC-Mamba**	**No regime**	**Rel. %**	**PICP RC**	**PICP ctrl.**	**Width RC**	**Width ctrl.**
Calm	368	33.2	0.00527	0.00524	−0.63	80.3	76.8	0.0244	0.0203
Transition	28	2.5	0.00543	0.00549	+1.04	73.4	69.8	0.0223	0.0197
Stress	714	64.3	0.00534	0.00529	−0.92	77.2	73.6	0.0231	0.0201
All origins	1,110	100.0	0.00532	0.00528	−0.77	78.1	74.6	0.0235	0.0202

S7 Supplementary Figures and Tables

Table S12: Positioning of RC-Mamba relative to prior work. ● = supported; ○ = absent or partial.

Method	SSM backbone	Regime cond.	Exog. covariates	Prob. output	Multi-asset	Walk-forward
Hamilton (1989)	○	●	○	●	○	○
MS-GARCH (Gray, 1996)	○	●	○	●	○	○
TFT (Lim et al., 2021)	○	○	●	●	●	●
DeepAR (Salinas et al., 2020)	○	○	●	●	●	●
N-BEATS (Oreshkin et al., 2020)	○	○	○	○	●	●
Mamba (Gu and Dao, 2023)	●	○	○	○	○	○
DSSM (Rangapuram et al., 2018)	●	○	●	●	●	○
**RC-Mamba** (ours)	●	●	●	●	●	●

Table S13: Key notation used throughout this paper.

Symbol	Description
B	Batch size
T	Historical look-back length
H	Forecast horizon
K	Number of HMM regimes (K=3)
D	HMM observation dimensionality (D=3)
d_{model}	Hidden dimension (d_{model}=64)
d_{var}	Per-variable projection dimension (d_{var}=64)
d_{state}	SSM state dimension (d_{state}=16)
d_{inner}	Mamba expansion dimension (d_{inner}=2d_{state}=32)
d_s	Static embedding dimension (d_s=16)
$\boldsymbol{\gamma}_t$	Regime posterior: $\gamma_{t,k} = P(z_t{=}k \mid \mathbf{o}_{1:t})$
$\boldsymbol{\gamma}_{\text{hist}}$	Historical posteriors $\in \mathbb{R}^{B\times T\times K}$
$\boldsymbol{\gamma}_{\text{future}}$	Propagated posteriors $\in \mathbb{R}^{B\times H\times K}$
$\mathbf{A}$	HMM transition matrix $\in \mathbb{R}^{K\times K}$
σ_u	RUG scale factor $\in$ [0.5, 2.0]

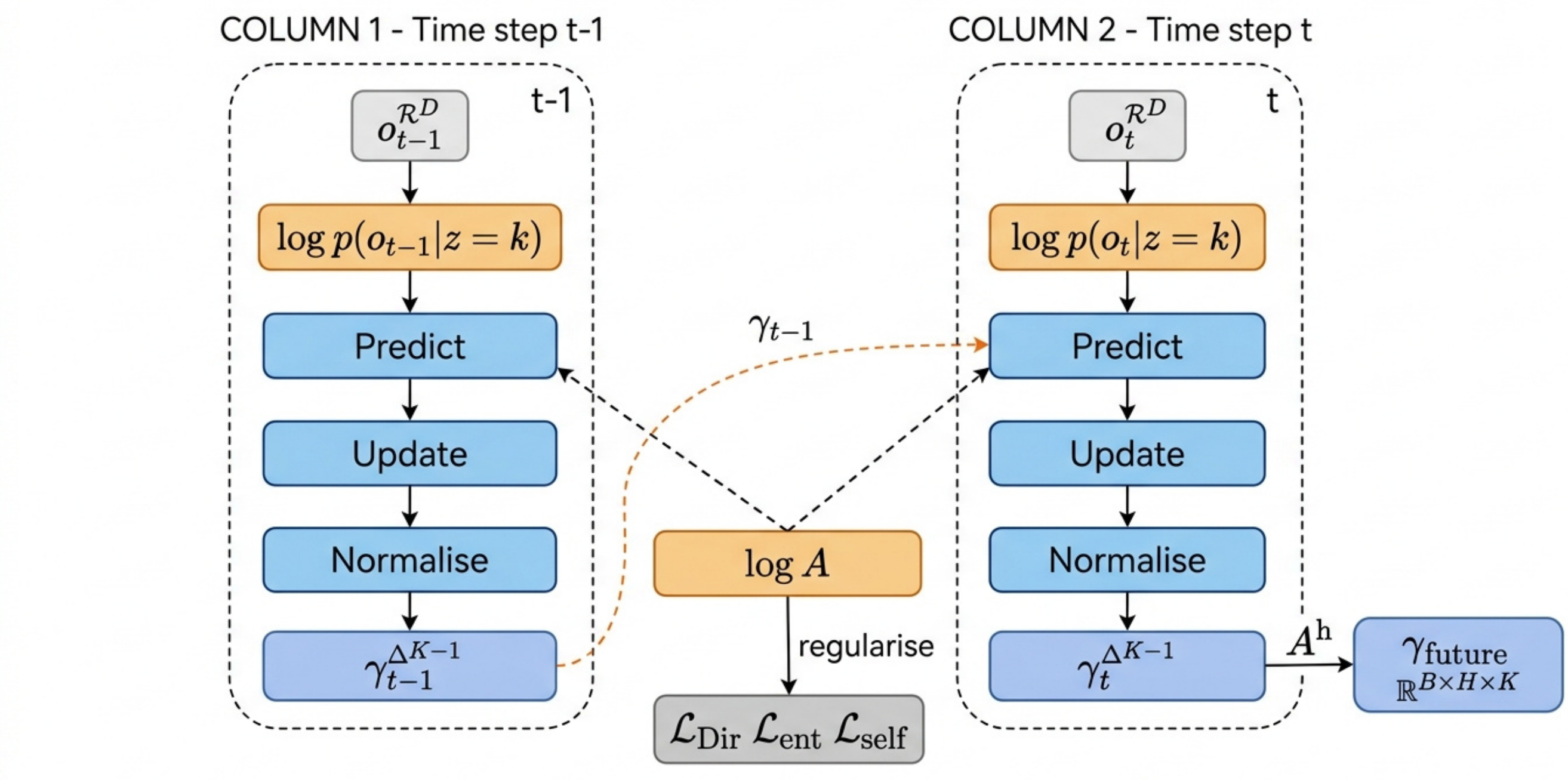

Figure S2: HMM causal forward filtering. Two consecutive time steps ($t-1$, t) illustrate the predict–update–normalise cycle. The shared log-space transition matrix $\log \mathbf{A}$ feeds both filter columns and is jointly regularised by sticky Dirichlet, entropy, and self-transition losses. Future posteriors are propagated via iterated matrix–vector multiplication.

Table S14: RC-Mamba block conditioning modes.

Mode	Δ	B	C	Description
`full`	γ	γ	γ	All SSM params conditioned
`delta_only`	γ	—	—	Only step size is regime-aware
`none`	—	—	—	Regime-agnostic baseline

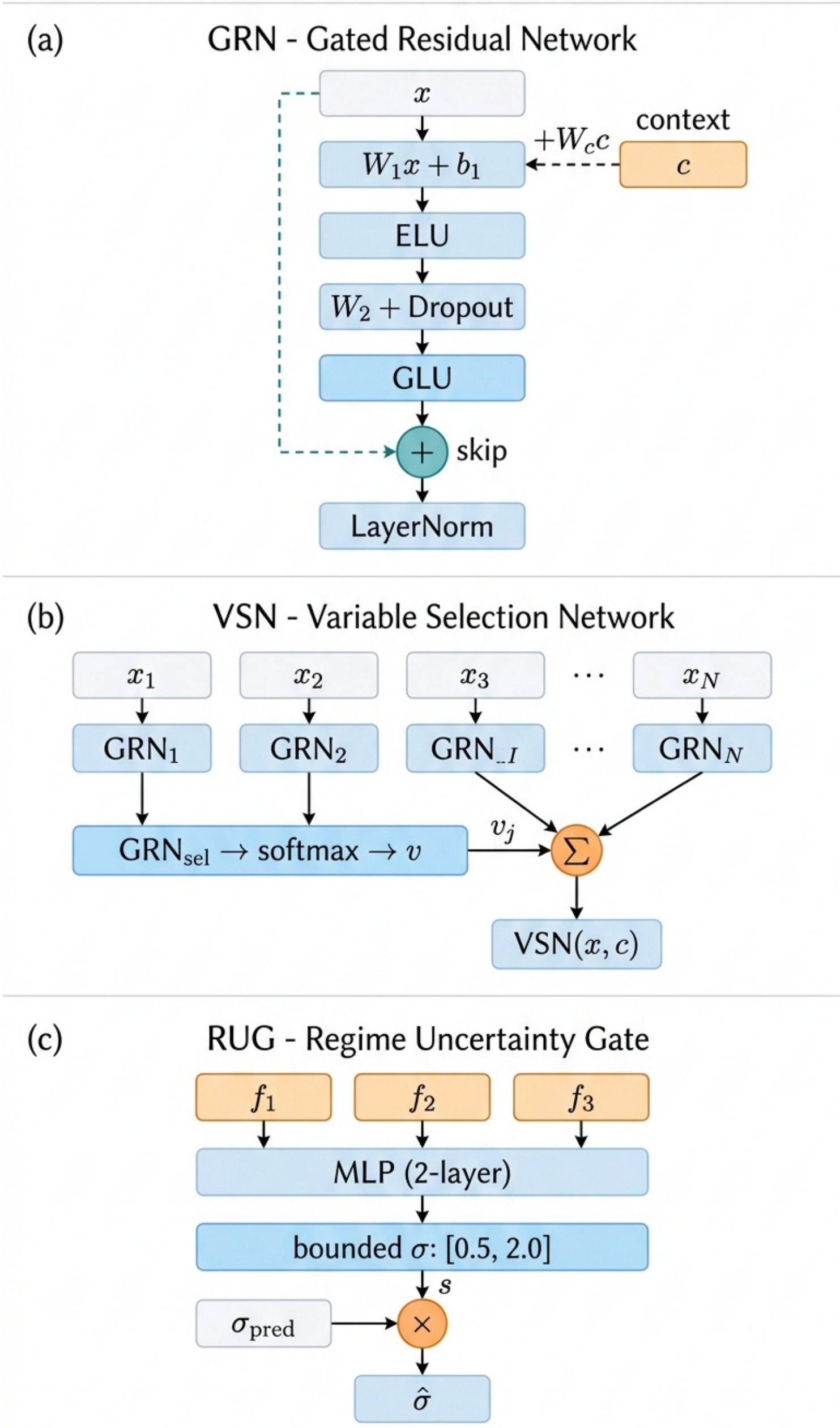

Figure S3: Component detail diagrams. **(a)** Gated Residual Network (GRN) with optional context conditioning. **(b)** Variable Selection Network (VSN) selecting among N input variables via softmax-weighted aggregation. **(c)** Regime Uncertainty Gate (RUG) mapping three posterior diagnostics (f_1: normalised entropy, f_2: peak probability, f_3: transition uncertainty) to a bounded scale modulator $s \in [0.5, 2.0]$.

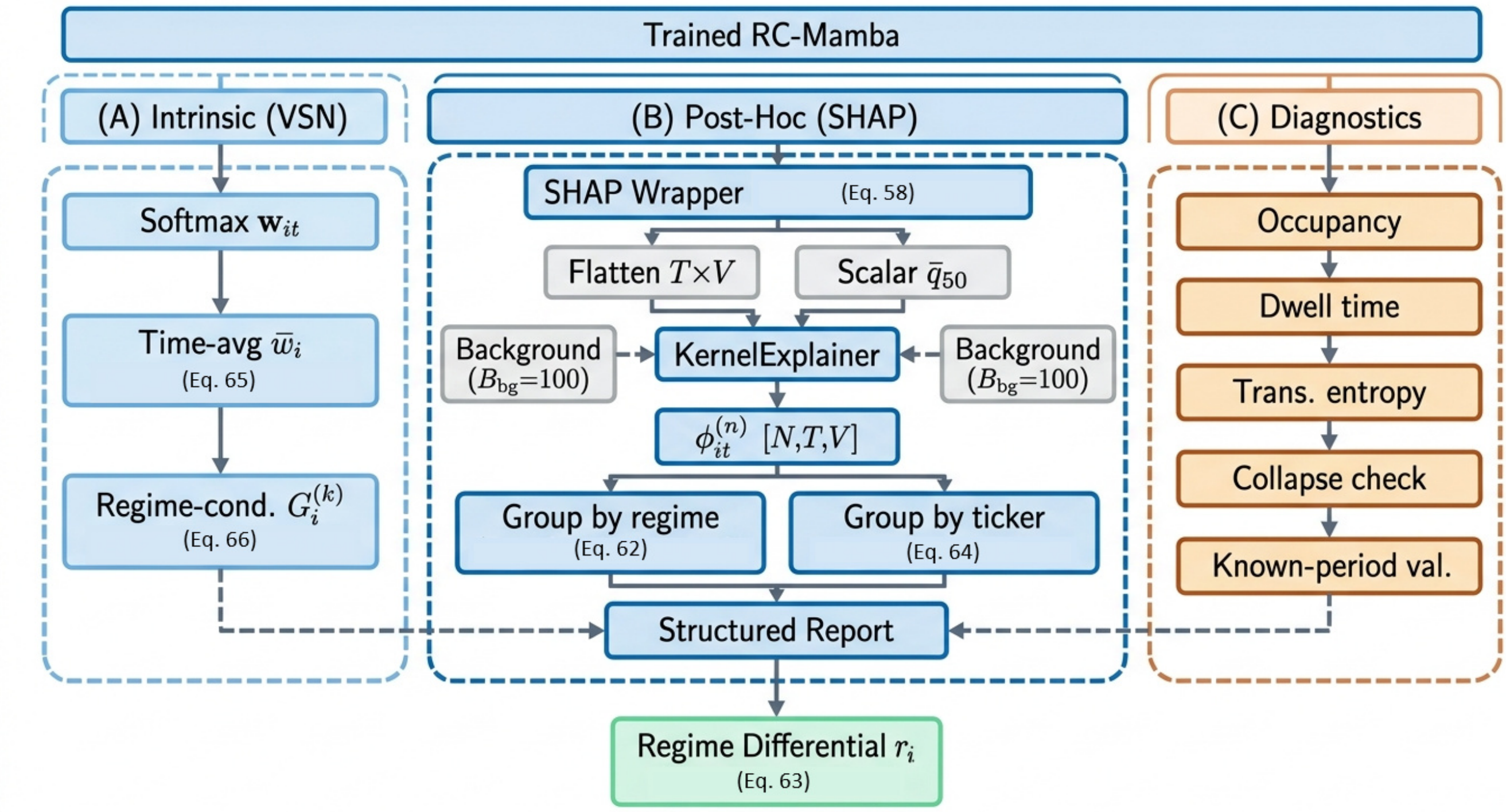

Figure S4: Regime-conditioned interpretability pipeline. Three complementary pillars produce layered explanations: (A) intrinsic VSN attention weights provide global and regime-weighted feature rankings; (B) post-hoc KernelSHAP attributions are grouped by dominant HMM regime and by ticker, yielding regime-differential ratios r_i; (C) HMM diagnostics validate regime quality via occupancy, dwell-time, transition entropy, and known-period overlap tests. All three feeds converge into a structured interpretability report.

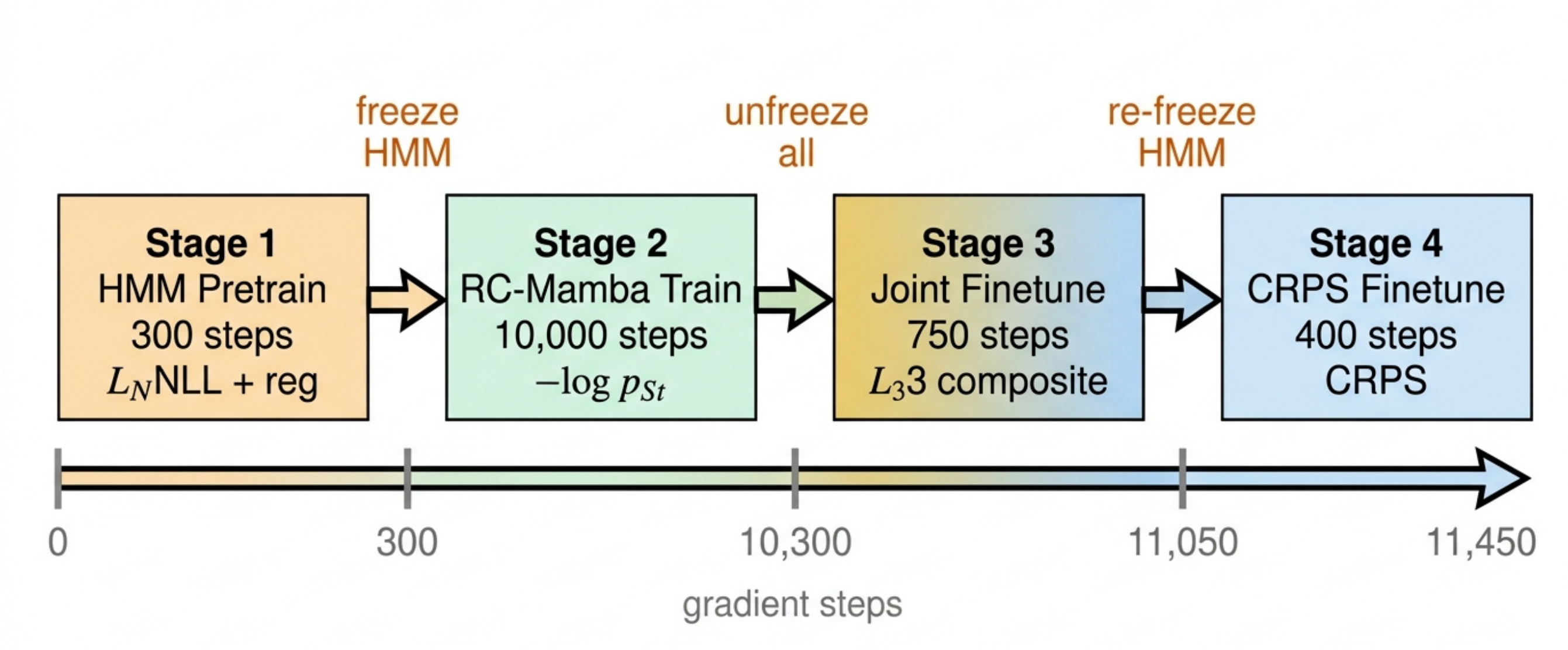

Figure S5: Four-stage training protocol. Stage colours indicate which parameter groups are being trained: HMM-only (orange), forecasting network (green), all parameters jointly (gold), and CRPS calibration (blue). The HMM is frozen in Stages 2 and 4 to prevent destabilisation of the learned regime structure by forecast or calibration gradients.

Table S15: Four-stage training protocol summary.

	**Stage 1**	**Stage 2**	**Stage 3**	**Stage 4**
Steps	300	10,000	750	400
Optimiser	Adam	AdamW	AdamW	AdamW
LR	10^{-3}	10^{-3}	$\approx 10^{-4}$	$\approx 10^{-5}$
Weight decay	0	10^{-4}	10^{-4}	10^{-5}
Grad clip	1.0	1.0	sep.	0.5
Scheduler	—	cosine	—	—
Val interval	—	100	50	50
ES patience	—	10	5	5
HMM state	train	frozen	train	frozen

"sep." = separate gradient clipping for HMM and non-HMM parameter groups.

Table S16: Historical exogenous features ($V_h = 30$) grouped by category, with publication lags applied during walk-forward evaluation.

**Category**	**Feature**	**Lag (days)**	**Source**
Market	High, Low, Open	0	Exchange
	Volume	0	Exchange
	H−L (range)	0	Derived
	O−C (body)	0	Derived
Price-Derived	3-day MA, 10-day MA	0	Derived
	Std_dev (rolling σ)	0	Derived
	Returns (simple)	0	Derived
	Increase_Decrease	0	Derived
	Buy_Sell, Buy_Sell_on_Open	0	Derived
	NSS (news sentiment)	0	Aggregator
Treasury/ Monetary	VIXCLS	0	CBOE/FRED
	DGS3MO, DGS2, DGS10	0	FRED
	DFII10 (TIPS real yield)	0	FRED
	DGS10−DGS2 (term spread)	0	Derived
	Breakeven rate	0	Derived
	FEDFUNDS	5	FRED
	WALCL (Fed balance sheet)	2	FRED
Macro	CPALTT01USM661S (CPI)	30	OECD/FRED
	GDPC1 (real GDP)	60	BEA/FRED
	DTWEXBGS (USD index)	0	FRED
	EMVOVERALLEMV (EPU)	5	PolicyUncertainty
	EMVMACROBUS	5	PolicyUncertainty
	EMVMACROINTEREST	5	PolicyUncertainty
Credit	BAMLH0A0HYM2 (HY spread)	0	ICE BofA/FRED

Table S17: Revised walk-forward fold splits. Partition boundaries are common calendar dates rather than per-ticker positional indices, and a 30-business-day embargo separates training, validation and test partitions by target-end date. All 18 assets are eligible in every fold and the five test blocks are disjoint.

**Fold**	**Test year**	**Test origins**	**Target dates**	**Assets**	**Forecast cells**
0	2021	223	252	18	120,420
1	2022	222	251	18	119,880
2	2023	221	250	18	119,340
3	2024	223	252	18	120,420
4	2025	221	250	18	119,340

Table S18: Top-5 hyperparameter configurations evaluated on fold 0. Configuration #1 is selected as the final configuration and applied across all five folds.

**Rank**	**Val Loss**	$\boldsymbol{\eta}$	**Dropout**	**WD**	$\boldsymbol{\eta_{\text{hmm}}}$	**HMM Clip**	**HMM Mult**
1	-2.9846	3×10^{-3}	0.10	10^{-4}	10^{-3}	1.0	1.0
2	-2.9683	2×10^{-3}	0.10	10^{-4}	10^{-3}	1.0	1.0
3	-2.9158	10^{-3}	0.10	10^{-3}	5×10^{-4}	1.0	0.5
4	-2.8977	10^{-3}	0.10	10^{-4}	10^{-3}	1.0	1.0
5	-2.8891	10^{-3}	0.20	10^{-4}	10^{-3}	1.0	1.0

Table S19: Selected RC-Mamba architecture and training configuration.

**Component**	**Parameter**	**Value**
Architecture	d_{model} (hidden dim)	64
	d_{var} (variable dim)	64
	d_{static} (static embed)	16
	d_{state} (SSM state)	16
	K (HMM regimes)	3
	Conv kernel size	3
	Dropout	0.1
HMM	Emission type	Student-t
	ν_{init} (df init)	1.0
	Variance floor	10^{-4}
	Observations	VIX, HY spread, term spread
RUG	Scale range	$[0.5,\ 2.0]$
	MLP hidden dim	32
	Features	entropy, max prob, transition unc.
Output	Distribution	Student-t ($\nu > 3$)
	MC samples (eval)	1,000
Conditioning	Mode	full (Δ, B, C)
	SiLU gate	disabled
	Ticker adapter (FiLM)	enabled
Occam Discarded	Macro VSN	off (p=0.78)
	Bounded regime gate	off
	Ticker embed regularisation	off

Table S20: Per-fold CRPS and $PICP_{80}$ under the revised protocol. Fold 1 (test year 2022) is the hardest window for each of the five competitive models, at 1.3–1.6 times its mean CRPS over the remaining folds, and is where coverage falls furthest from nominal. LSTM and N-BEATS are the exceptions: both record their worst fold at fold 2.

Model / metric	Fold 0 (2021)	Fold 1 (2022)	Fold 2 (2023)	Fold 3 (2024)	Fold 4 (2025)
RC-Mamba	0.00432	0.00767	0.00485	0.00489	0.00488
$PICP_{80}$	83.6	60.9	72.8	92.4	80.7
VanillaTFT	0.00509	0.00762	0.00515	0.00510	0.00496
$PICP_{80}$	94.3	71.0	91.8	92.8	84.4
DeepAR	0.00485	0.00781	0.00594	0.00498	0.00562
$PICP_{80}$	92.3	83.8	89.0	90.8	88.6
S-Mamba	0.00530	0.00780	0.00610	0.00588	0.00631
$PICP_{80}$	92.6	75.9	89.5	91.6	95.7
HMM-DeepAR	0.00659	0.00904	0.00621	0.00550	0.00530
$PICP_{80}$	93.4	44.6	95.0	91.9	89.2
LSTM	0.00467	0.00977	0.03844	0.00499	0.00526
$PICP_{80}$	79.1	66.5	95.4	81.8	68.6
N-BEATS	0.00505	0.22231	0.26331	0.03907	0.00870
$PICP_{80}$	52.9	14.8	84.4	21.4	77.9

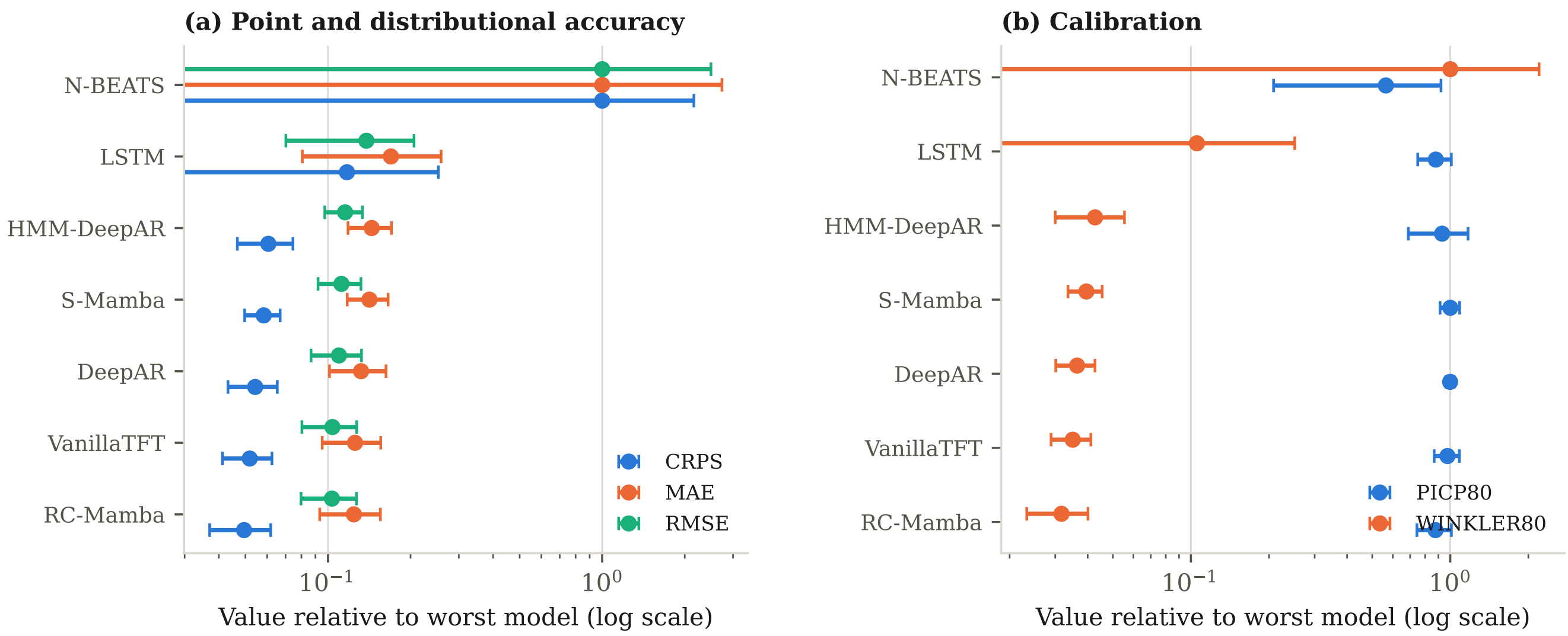

Figure S6: Dot-whisker comparison across five walk-forward folds. **(a)** Point accuracy metrics (CRPS, MAE, RMSE, directional accuracy); **(b)** calibration quality (Winkler score, PINAW, PICP proximity to nominal 80 % coverage). Horizontal bars show ±1 std across folds. N-BEATS excluded due to marked under-performance (see Section 4.3). RC-Mamba (blue) achieves the best trade-off between accuracy and calibration sharpness. Note that a short whisker is not by itself a merit: S-Mamba and the Vanilla TFT have tighter cross-fold dispersion than RC-Mamba at uniformly worse mean CRPS.

Table S21: Dual-lens feature importance from post-hoc grouped permutation SHAP (128 samples, all five final checkpoints, 64 antithetic permutations, maximum local-accuracy error 3.14×10^{-9}). Features are ranked by SHAP attribution; the intrinsic VSN weight is shown alongside. The two lenses disagree on ordering, so intrinsic gate weights alone are an unreliable interpretability signal.

**Rank**	**Feature**	**Mean $\lvert\phi_j\rvert$ ($\times 10^{-4}$)**	**VSN weight (%)**
1	`EMVOVERALLEMV`	1.522	2.58
2	`RBUSBIS`	1.269	4.26
3	`WALCL`	1.097	3.08
4	`EMVMACROBUS`	1.046	8.93
5	`PAYEMS`	1.023	4.35
6	`FEDFUNDS`	0.953	2.38
7	`GDPC1`	0.899	5.04
8	`EMVMACROINTEREST`	0.783	4.57
9	`DTWEXBGS`	0.770	4.96
10	`DGS3MO`	0.745	1.60

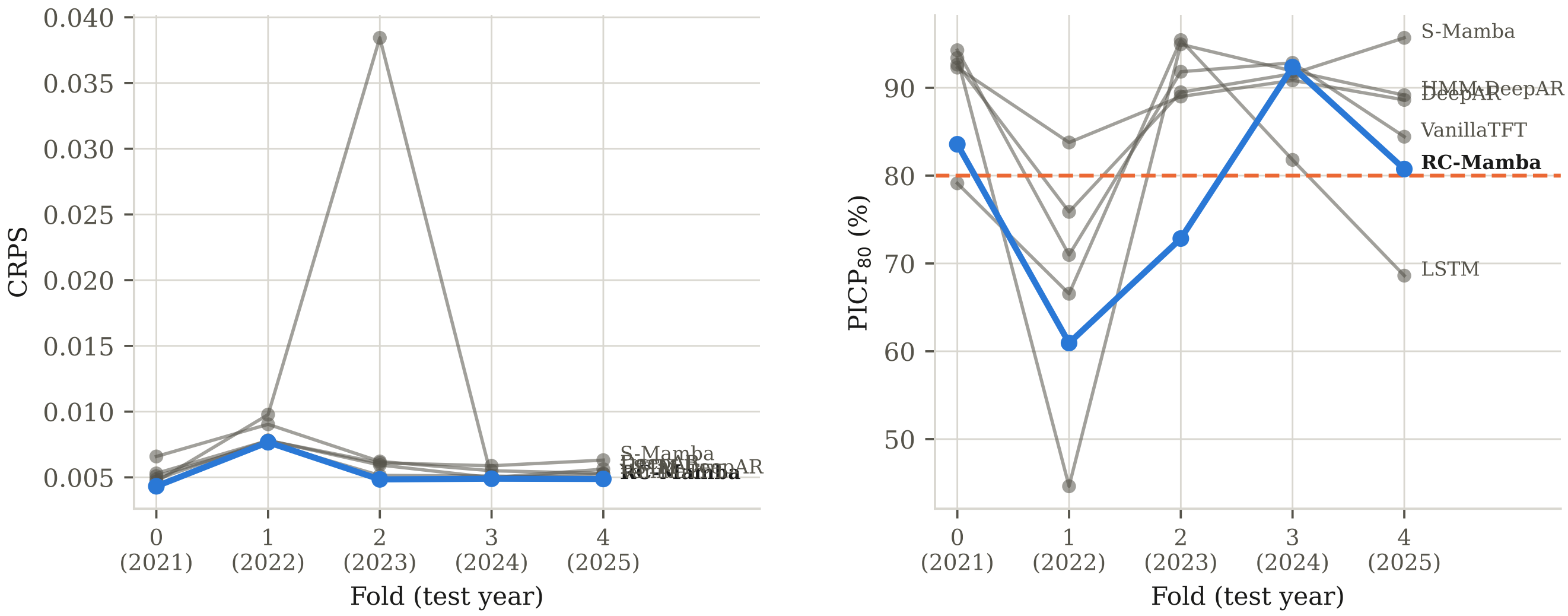

Figure S7: Per-fold trajectories (N-BEATS excluded for readability, its fold-1 and fold-2 CRPS being two orders of magnitude above the panel). **(a)** CRPS across folds: RC-Mamba is lowest in four of the five folds and second in fold 1, where the Vanilla TFT leads by 0.7 %. The LSTM spikes to 0.0384 in fold 2. **(b)** PICP_{80}: coverage is unstable for every model. RC-Mamba spans 60.9 % (fold 1) to 92.4 % (fold 3); HMM-DeepAR falls to 44.6 % in fold 1 and the LSTM over-covers at 95.4 % in fold 2. The dashed orange line marks the nominal 80 % coverage target.

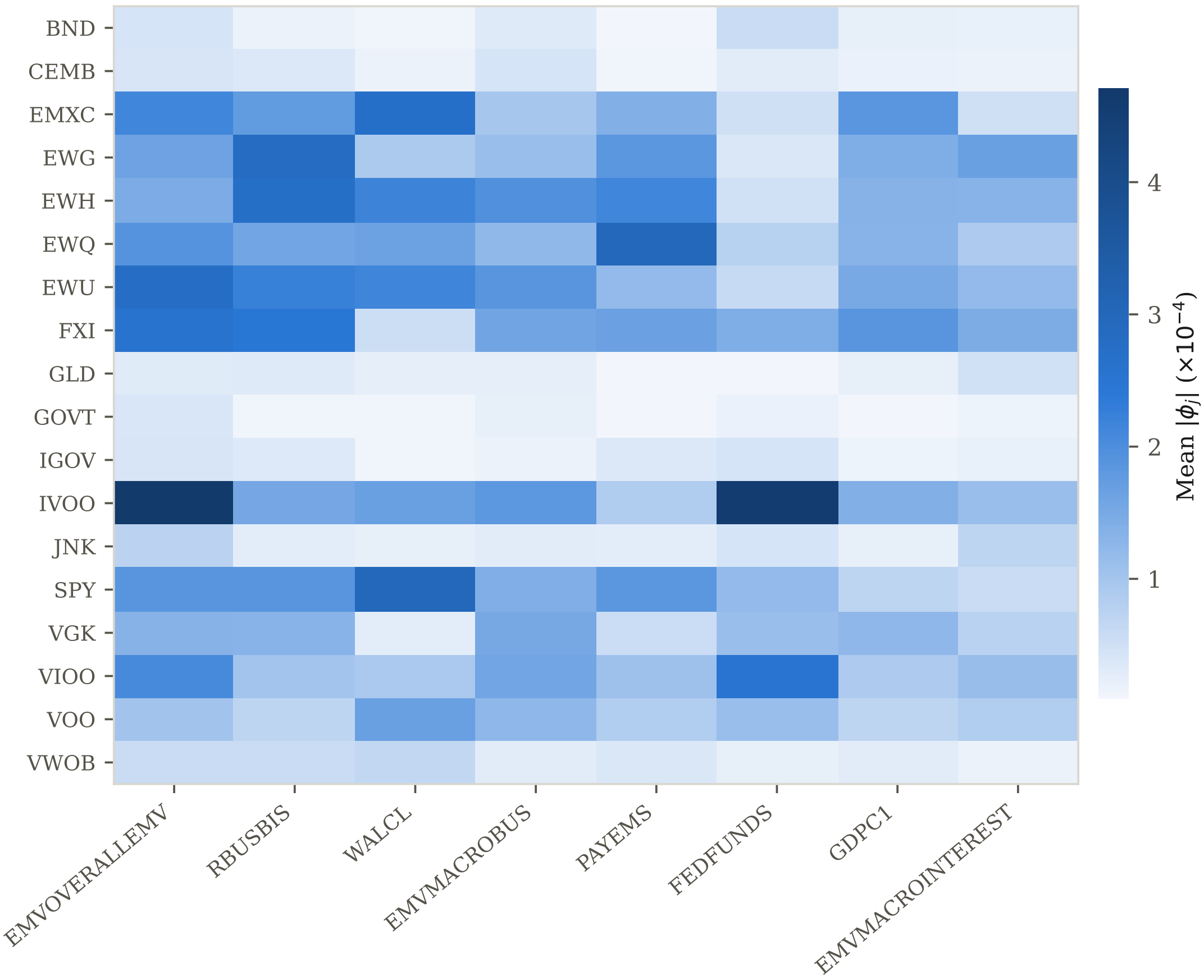

Figure S8: Asset-level SHAP attribution across the 18 ETFs for the eight most influential macro-financial features, from the post-hoc permutation SHAP analysis of the revised RC-Mamba checkpoints. Attribution patterns differ systematically across asset classes.

Table S22: Component ablations on both matched batches. Each batch uses a single fold with a common seed, data pipeline and training schedule; Δ is relative to the selected gate-off configuration within the same batch. Four of seven contrasts change sign between folds, so the component effects (0.3–2 %) are smaller than the fold-to-fold variation and no single-fold ablation identifies them. This is why the corresponding claims in the main text are stated as design trade-offs.

	**Fold 0 (2021)**		**Fold 1 (2022)**		
**Variant**	**CRPS**	**Δ %**	**CRPS**	**Δ %**	**Replication**
RUG fixed to scale one	0.00438	+1.38	0.00759	−1.09	SIGN FLIPS
Learned global scale	0.00429	−0.66	0.00760	−0.92	replicates (better)
Continuous macro conditioning	0.00441	+2.08	0.00763	−0.54	SIGN FLIPS
Full conditioning, gate on	0.00430	−0.55	0.00764	−0.35	replicates (better)
Full conditioning, gate off (ref.)	0.00432	—	0.00767	—	reference
Posterior appended as input	0.00440	+1.88	0.00773	+0.81	replicates (worse)
Condition Δ only	0.00430	−0.40	0.00776	+1.19	SIGN FLIPS
No regime conditioning	0.00429	−0.80	0.00783	+2.05	SIGN FLIPS
